# Jurassic climate mode governed by ocean gateway

**DOI:** 10.1038/ncomms10015

**Published:** 2015-12-11

**Authors:** Christoph Korte, Stephen P. Hesselbo, Clemens V. Ullmann, Gerd Dietl, Micha Ruhl, Günter Schweigert, Nicolas Thibault

**Affiliations:** 1 Department of Geosciences and Natural Resource Management, University of Copenhagen, Øster Voldgade 10, 1350 Copenhagen-K, Denmark; 2 Camborne School of Mines and Environment and Sustainability Institute, University of Exeter, Penryn Campus, Treliever Road, Penryn, Cornwall TR10 9FE, UK; 3 Staatliches Museum für Naturkunde Stuttgart, Rosenstein 1, 70191 Stuttgart, Germany; 4 Department of Earth Sciences, University of Oxford, South Parks Road, Oxford OX1 3AN, UK

## Abstract

The Jurassic (∼201–145 Myr ago) was long considered a warm ‘greenhouse' period; more recently cool, even ‘icehouse' episodes have been postulated. However, the mechanisms governing transition between so-called Warm Modes and Cool Modes are poorly known. Here we present a new large high-quality oxygen-isotope dataset from an interval that includes previously suggested mode transitions. Our results show an especially abrupt earliest Middle Jurassic (∼174 Ma) mid-latitude cooling of seawater by as much as 10 °C in the north–south Laurasian Seaway, a marine passage that connected the equatorial Tethys Ocean to the Boreal Sea. Coincidence in timing with large-scale regional lithospheric updoming of the North Sea region is striking, and we hypothesize that northward oceanic heat transport was impeded by uplift, triggering Cool Mode conditions more widely. This extreme climate-mode transition provides a counter-example to other Mesozoic transitions linked to quantitative change in atmospheric greenhouse gas content.

## Abstract (web summary)

Dynamics of the Laurasian Seaway are thought to have had wide effects on oceanography and climate in the mid-Mesozoic. Here, the authors show evidence for seawater temperature change, ascribed to tectonic uplift that impeded poleward oceanic heat transport and triggered a cool climate mode in the earliest Middle Jurassic.

## Graphical abstract

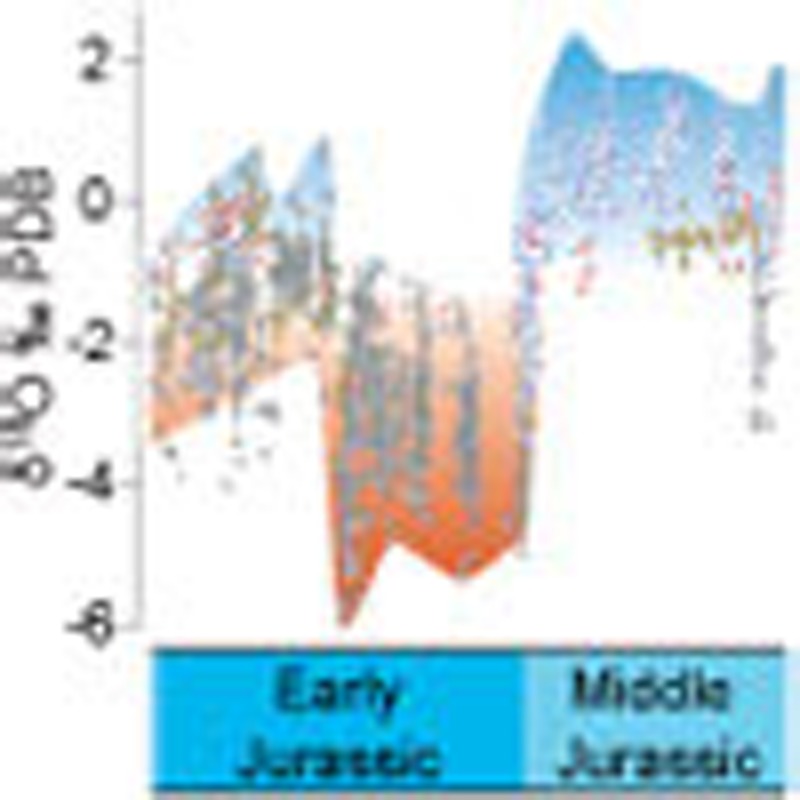

Cold and warm climate ‘modes' in the Jurassic have been suggested^1^ on the basis of palaeobiogeographic and oxygen isotopic evidence^2^,^3^. In support of cold modes, several authors have noted the occurrence of glendonite (predominantly cold-water calcite pseudomorphs) and ice-rafted debris in circum-Arctic basins^4^,^5^,^6^, although whether significant continental ice sheets developed during the Jurassic is debatable^7^. In contrast, the Toarcian Oceanic Anoxic Event (T-OAE) in the Early Jurassic (∼182 Ma) stands out as a very warm episode^8^,^9^. The origins of such warm interludes are well investigated^10^,^11^, but the character and origins of the cold periods in the Jurassic are not understood.

The area of western Europe was situated between palaeolatitudes 30–45° N (ref. 12). Much of the region was traversed by epicontinental seas forming the so-called ‘Laurasian' Seaway that connected the low-palaeolatitude Tethys Ocean in the south to the high-palaeolatitude Boreal Sea via the Viking Corridor in the north (Fig. 1), and some authors have suggested that seaway dynamics had wide effects on palaeoceanography and climate^13^.

Here, we present new data comprising oxygen-isotope ratios from well-preserved late Pliensbachian to Bajocian (191–168 Ma) calcite macrofossils of the Laurasian Seaway area, which we use to reconstruct past seawater temperatures. The new data set is unprecedented in terms of degree of detail and stratigraphic precision for this interval of time. Diagenetically resistant low-Mg-calcite fossils (belemnites, bivalves and brachiopods) were screened for post-depositional alteration and only those samples lacking physical or chemical signs of alteration were regarded as preserving the original carbonate δ^18^O (Methods section), following the detailed explanations recently fully reviewed in ref. 14. We infer an abrupt earliest Middle Jurassic (∼174 Ma), mid-latitude, cooling of seawater by as much as 10 °C, and we conclude that tectonic influence on seaway connectivity had far-reaching effects on palaeoclimate.

## Results

### Oxygen isotopes

Large oxygen-isotope fluctuations occur over the studied interval (Fig. 2 and Supplementary Data 1), highlighting the Late Pliensbachian Cool Event^15^,^16^, the warm episode during the T-OAE (refs 9, 17), and the Middle Jurassic cold interval^18^,^19^ earlier described as a long-term trend to cooler temperatures^7^. Here we show for the first time that this latter positive δ^18^O shift is very sharp and occurs precisely in the earliest Aalenian just after the Early to Middle Jurassic boundary (Toarcian–Aalenian; Fig. 2).

In the Hebrides Basin the shift to heavier values is >2.5‰ (Fig. 3). Both positive and negative isotopic trends in the data set are the same for the different basins and provinces with the largest amplitudes recorded in the Cleveland and Hebrides basins, smaller amplitudes in the Swabo-Franconian Basin, and smallest amplitudes in the Lusitanian and Basque-Cantabrian basins (Fig. 2). The new data set also shows that the negative oxygen-isotope excursion during the T-OAE is transient and superimposed on an overall warming trend through the entire Toarcian in the Cleveland Basin. The Toarcian data from the Hebrides are especially significant as they confirm equivalently warm temperatures in both the Hebrides and Cleveland basins during the Toarcian. Thus the early Aalenian presents one of the most pronounced δ^18^O inclines observed for Phanerozoic data sets. The resulting heavy values persist during the Aalenian and at least the early Bajocian (that is, for >∼5 Myr), with some significant short-term returns to warm conditions (for example, in the *humphresianum* zone: biosubzone no. 72; Fig. 2 and Supplementary Table 1).

### Carbon isotopes

Carbon-isotope data (Supplementary Data 1) generated from the same sample set do not show a marked change at the Toarcian–Aalenian transition, but instead show a long-term decline in values across the same time span as oxygen isotope become substantially heavier (Supplementary Figs 1 and 2).

## Discussion

Taxa analysed for this study were selected on the basis of lack of observed vital effects on oxygen-isotope ratios in biogenic calcite of modern representatives^16^,^20^. Changes in the oxygen isotopic composition of calcite shells might also partly reflect changes in seawater δ^18^O or pH^2^. However, given the ubiquitous normal marine salinities evident from the studied fossil assemblages, neither factor can have had a significant influence on shell δ^18^O. Only for the T-OAE, characterized in the seaway by up to 10 m of laminated black shale, has it been suggested that Laurasian Seaway salinity deviated from the long-term mean, in this case by influx of freshwater^21^,^22^. The 2.5‰ δ^18^O increase in the early Aalenian might alternatively be explained by evaporative concentration of the heavy isotope of oxygen, but that would imply hypersalinity when fossil and sedimentological evidence demonstrate stenohaline conditions through the whole late Toarcian to early Aalenian interval^23^,^24^,^25^.

We therefore conclude that the observed strong shift towards heavy δ^18^O values in the early Aalenian principally reflects seawater temperature change, indicating about 10 °C of cooling over a period of about 0.5 Myr, and lowest temperatures of ∼4 °C using the standard assumption^26^ of −1‰ (Standard Mean Ocean Water) for seawater δ^18^O (Fig. 2). Some calcite macrofossil taxa stopped calcification in cool seasons, suggesting that the new data set might not record the coldest cold-season water temperatures, and that warm season temperatures in these climatic regions are overrepresented^27^. Such a mechanism may explain also the lower variability of δ^18^O in the Hebrides Basin relative to the Cleveland Basin (Fig. 3, Supplementary Fig. 1, and ref. 28). Lighter oxygen-isotope values and more variability are evident from the northerly Hebrides and Cleveland basins relative to the records from the Lusitanian and Basque-Cantabrian basins; this likely results from shallower water habitats in northerly basins (∼100 versus ∼200 m)^13^,^29^ and a latitudinal salinity effect of about ∼1 per mil (cf. ref. 30).

The same late Pliensbachian to Bajocian palaeoclimatic fluctuations have also been suggested for the Arctic region, based principally on mineralogical, palaeontological and sedimentological data. Significant cooling has been inferred for the late Pliensbachian and the earliest Middle Jurassic (including the Aalenian and Bajocian) based on glendonite occurrence, ice-rafted debris, and low marine diversity, as well as palynology; in contrast, a temperature maximum in the early Toarcian is evidenced by palynological data indicative of northward expansion of terrestrial floras^4^,^31^,^32^,^33^.

Over the studied interval there is no clear correlation between the oxygen isotope and carbon-isotope records (Fig. 3; Supplementary Fig. 2). Therefore an explanation for the palaeotemperature changes based on inferred marine organic carbon burial is not supported by the data. In addition, positive carbon-isotope excursions for latest Toarcian to Bajocian successions of other regions in the Laurasian Seaway^18^,^19^,^34^,^35^,^36^,^37^ are different in timing and magnitude^19^, and have been related to more local enhanced biological activity of eutrophic phytoplankton and radiolarians which strengthened the biological pump^36^.

**Figure 1:**
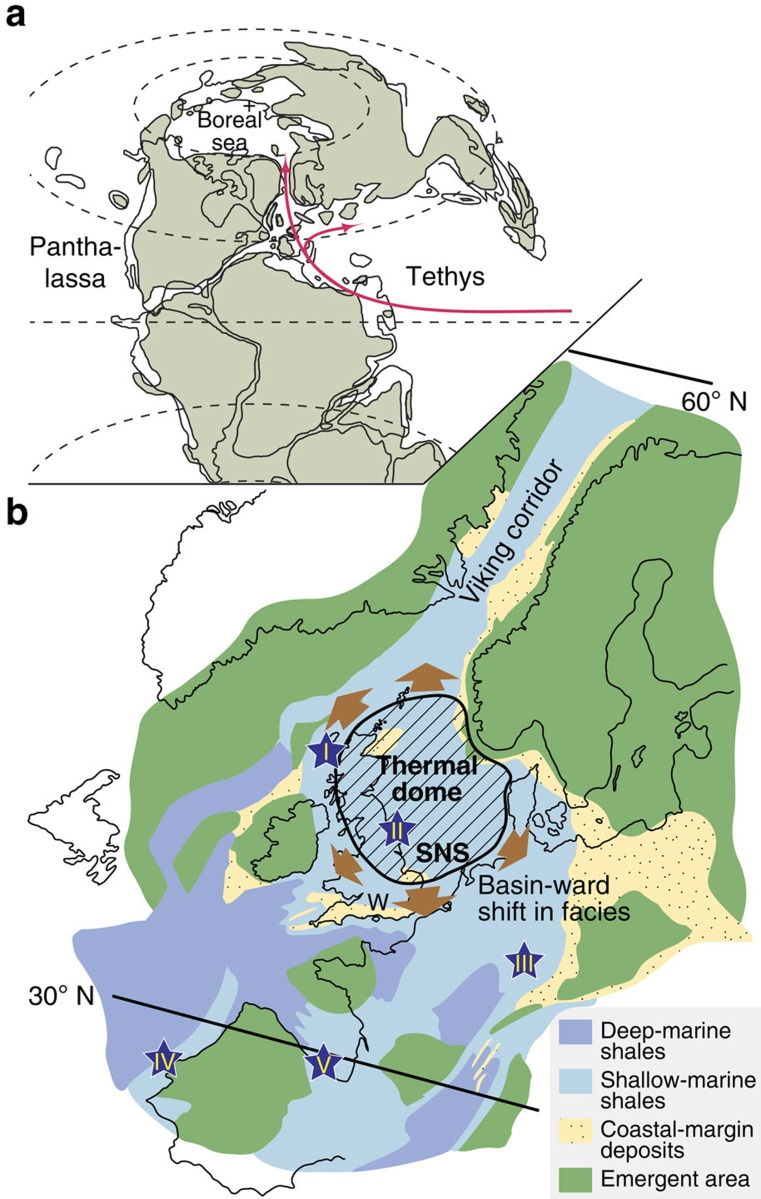
Late Early Jurassic palaeogeography. (**a**) Map shows the connection between the equatorial Tethys Ocean and the Boreal Sea via the Laurasian Seaway; the latter including the Viking Corridor which was several hundred kilometres wide^43^. Red arrows mark generalized palaeocurrents (see text, ref. 44, and references therein). (**b**) Detail of Laurasian Seaway palaeogeography with the region affected by North Sea Dome as determined by the generalized outer limit of the Toarcian subcrop^40^. Brown arrows represent the siliciclastic sediment supply/transport in relation to domal uplift^39^,^40^. Sample locations are numbered and identified by stars (Hebrides Basin (I; Scotland), Cleveland Basin (II; England), Swabo-Franconian Basin (III; Germany) and Lusitanian (IV; Portugal)/Basque-Cantabrian basins (V; Spain)). SNS, Southern North Sea Basin, W, Wessex Basin. Figures modified from refs 12, 45, 44 and references therein.

**Figure 2:**
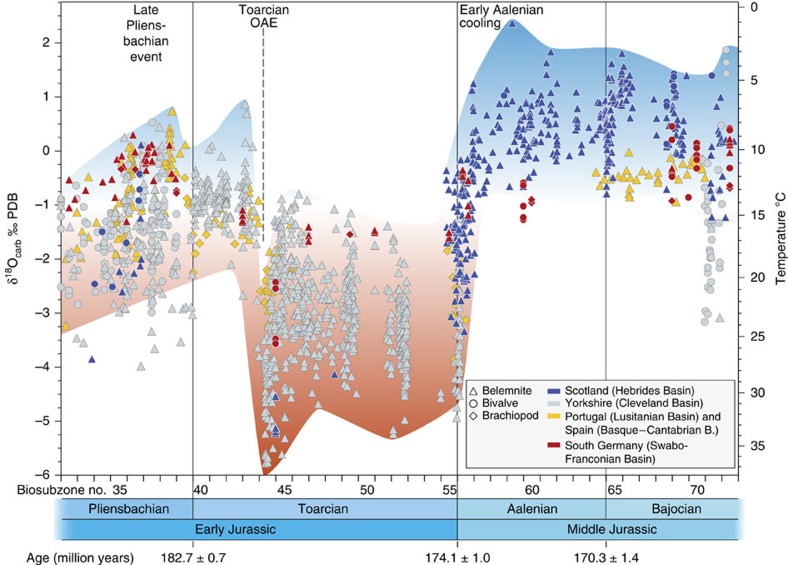
Pliensbachian to Bajocian oxygen-isotope record plotted against subzone numbers. Data obtained from pristine and biostratigraphically well-constrained marine calcite fossils from selected European basins (Methods section, Supplementary Data 1, Supplementary Table 1). Shading behind the oxygen-isotope data highlight warm (red) and cool (blue) palaeotemperatures. Plot symbol shape indicates macrofossil type and colour indicates locality. The plot highlights the abrupt and large magnitude shift towards cold seawater temperatures in the earliest Middle Jurassic which, in Hebrides Basin, represents at least 10 °C (Fig. 3). The Late Pliensbachian Cold Event and the Toarcian Oceanic Anoxic (hot) Event are also represented. Superimposed higher frequency climate changes are present during the complete time span for all localities. Data ranges for specific times reflect expected (annual) temperature changes^16^,^27^ and habitat effects^28^.

We hypothesize that the uplift of the North Sea Dome, caused by a rising asthenospheric plume^38^,^39^,^40^, led to obstruction of a northward flowing current through the Viking Corridor and thus strongly reduced heat transport to the Arctic regions (Fig. 1). At the same time, cold Arctic waters were able to exert influence on marine palaeotemperatures at palaeolatitudes as low as 45° reaching at least the Hebrides Basin (Figs 1 and 2). A comparable scenario is the Cenozoic domal uplift of the Greenland–Scotland Ridge which regulated the flow of warm water into the North Atlantic^41^,^42^. In the Jurassic North Sea area regional uplift affected a zone of ∼1,250 km in diameter^38^,^39^,^40^. Timing of the uplift has been identified by reference to the early Aalenian unconformity, widely observed in seismic reflection and borehole data^39^,^40^. In addition, latest Toarcian and earliest Aalenian shoreline regression is indicated by sedimentary facies changes in the Cleveland Basin (Yorkshire), Wessex Basin (Dorset) and other North Sea perimetric basins, further indicating regional tectonic uplift^38^,^39^,^40^. Pronounced provincialism in ammonite faunas observed for both the Laurasian Seaway and Arctic regions^43^ initiated in the early Aalenian confirms a structural barrier between low and high latitudes^33^,^43^.

North Sea Dome uplift would have fundamentally modified palaeocean current patterns. The general Early Jurassic ocean circulation of the NW Tethys and adjacent shelf is thought to have been characterized by a gyre acting down to water depths of several hundred metres, perhaps modified by monsoonal processes (ref. 44 and references therein). The Jurassic palaeogeographic/palaeoceanic setting was similar to the modern North Atlantic Gulf Stream or North Pacific Kuroshio current with a general northward surface flow between ∼ 35 and 60°N (Fig. 1), although clearly it would be inappropriate to take the parallels between modern open ocean currents and their Jurassic seaway counterparts too far.

Early Jurassic ammonite distributions in the Laurasian Seaway show progressive homogenization of faunas from the latest Pliensbachian to early Toarcian, and re-establishment of distinct faunal provinces in the later Toarcian^45^, supporting earlier conclusions based on a wider range of invertebrate fossils evidencing northward faunal spread in the early Toarcian^32^,^46^,^47^,^48^,^49^,^50^. A strong northward flow in the Viking Corridor is also suggested for the later Middle Jurassic by invertebrate fossil distributions for times when the strait is known to have been open^43^. In contrast, southward current flow was suggested for the Toarcian based on model calculations^13^; a finding difficult to reconcile with both the stenohaline fossil distributions and associated oxygen-isotope data^9^,^28^,^46^,^48^,^50^ (Supplementary Fig. 1), all showing relatively warm waters in the Toarcian Laurasian Seaway. Directionality apart, the models are of particular value in that they emphasise the potential significance of the seaway for heat transport.

The mechanism suggested here for the transition from Toarcian Warm Mode to Aalenian-Bajocian Cool Mode may also have been responsible for the transition from an earlier Pliensbachian Warm Mode to a later Pliensbachian Cool Mode. The possibility of an early onset of North Sea doming is suggested by the occurrence of regressive facies in the late Pliensbachian of the North Sea region, similar to those of the late Toarcian^51^,^52^; namely the Drake Formation (Southern North Sea Basin), Scalpa Sand Formation (Hebrides Basin), Staithes Sand (Cleveland Basin), Downcliff Sand and Thorncombe Sand (Wessex Basin). A global sea-level rise of likely tectonic origin, with a weakly quantified magnitude in the order of up to 100 m, has been well documented for the early Toarcian^53^; thus any seaway bathymetric (that is, regional) effect from an early, Pliensbachian, North Sea domal uplift is likely to have been counteracted by eustasy, at least until the late Toarcian.

The Early to Middle Jurassic scenario elaborated here shows clearly that major modifications in Mesozoic oceanic current patterns have significant influence on large magnitude, abrupt climate change and potentially govern transformations between Warm/Cool climate modes. Although greenhouse gases may also have had strong influence on Mesozoic events, the T-OAE being a prime example, this is not necessarily the case for other highly significant transitions. The North Sea Dome triggered perhaps the largest palaeoceanographically induced climate change in the Jurassic, at least on a super-regional scale.

## Methods

### Sampling

Belemnites, ostreoids, pectinids, pinnids and brachiopods were collected in Yorkshire, NE England (Robin Hood's Bay–Hawsker Bottoms, Saltwick Nab, Ravenscar–BleaWyke, Staithes–Brackenberry Wyke and Hundale Point) and in the Inner Hebrides, Scotland (Bearerraig on the Isle of Skye and Druim an Aonaich on the Isle of Raasay; Supplementary Data 1 and Supplementary Table 2). Samples from the South German Swabo-Franconian Basin originate mainly from the Staatliches Museum für Naturkunde Stuttgart. A few additional samples from this basin were taken in the Aubach valley near Aselfingen in the Wutach area. The samples cover in total the stratigraphic interval from the *davoei* (late early Pliensbachian) to the *humphriesianum* (late early Bajocian) ammonite biozones (Supplementary Table 1). Previously published data (Fig. 2 and Supplementary Data 1) were added to the new data for several localities (Bearreraig^54^; Robin Hood's Bay, Castle Chamber, Staithes and Brackenberry Wyke^16^; Hawsker Bottoms, Saltwick Bay, Ravenscar and Blea Wyke^28^,^55^,^56^,^57^), and for coevally deposited successions of Scotland (Inverarish Burn and Beinn na Leac^18^), South Germany (Roadcut, D (ref. 54)), Portugal (Lusitanian Basin, Peniche^29^ and Cabo Mondego^54^) and Spain (Basque-Cantabrian Basin, Camino^58^,^59^, Santiurde de Reinosa^58^,^59^, San Andrés^58^,^59^ and Fuentelsaz^60^, the latter including the Global Boundary Stratotype Section and Point (GSSP) for the Aalenian Stage). Only literature data from stratigraphically well-defined and diagenetically well-screened samples were used for the plots and discussions to maintain the comparability to our new stratigraphically well-defined, well-screened and carefully selected samples, representing best preserved material available.

### Stratigraphy

**Figure 3:**
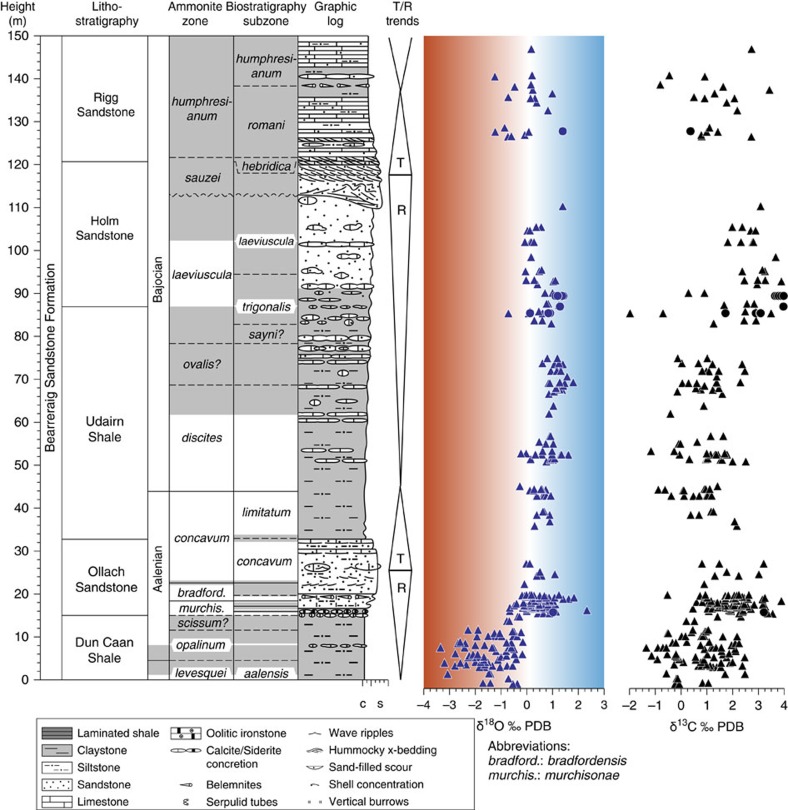
Oxygen and carbon-isotope data from the Bearreraig section in Scotland. Lithology, stratigraphy and transgressive/regressive (T/R) phases are from refs 25, 54, 76. Intervals lacking well-preserved ammonite fossils are indicated by grey shading and so denote limited age uncertainty. Shading in the graphic log denotes darker lithology. Transgressive/regressive phases denoted T/R. Shading behind the oxygen-isotope data highlight warm (red) and cool (blue) palaeotemperatures. The oxygen-isotope values increase abruptly in the *murchisonae* zone and at that time the unconformity was at maximum extent^35^,^36^. Note that this increase in the oxygen-isotope values occurs with a clear facies change from deeper to shallower water. There is a hint of other small palaeotemperature changes in conjunction with T/R cycles, but the magnitude of the T/R cycles and the palaeotemperature changes is small in comparison to the change across the Early/Middle Jurassic transition with perhaps the exception of the *humprhresianum* zone.

All sections sampled are biostratigraphically very well-constrained by ammonite biozonation, in many cases being amongst the most intensively investigated in the world, and the detailed subdivision is given in refs 25, 61, 62, 63, 64, 65. In the Bearreraig section (Fig. 3, Supplementary Data 1) no biostratigraphically significant taxa occur between 62 and 85.8 m. It was therefore necessary to approximate the base of the *ovalis* and *laeviuscula* zones and the *sayni*, *trigonalis* and *laeviuscula* subzones (Supplementary Data 1). Higher in the section we approximated the base of the *sauzei* zone (Fig. 3, Supplementary Data 1) at the highest level of the major erosion surface about 8–10 m below the top of Holm Sandstone. Zonal and subzonal boundaries from the *murchisonae* zone are based on heights below a prominent belemnite-rich bed (=marker bed O16, cf. ref. 25). We use the height of 19.65 m for the base of the *gigantea* subzone and 70.00 m for the base of the *ovalis* subzone (ref. 25).

### Selection

Belemnites, bivalves and brachiopods were chosen as substrates for analysis. The fossils could usually not be determined to finer taxonomic levels because generally only small fragments were available for collection (Supplementary Data 1); delineation of large-magnitude oxygen-isotope variations, however, is possible even with taxonomically undetermined specimens^2^,^16^.

### Screening and geochemical measurement

Despite their resistance to diagenesis, fossil specimens were carefully screened to identify potential post-depositional alteration which might have reset the primary geochemical signal^2^,^14^,^66^. Bivalve and brachiopod shells were prepared, optically inspected and hand-picked using a binocular microscope and needle^16^,^67^. For belemnites, fresh surfaces of broken pieces were inspected under the binocular microscope, and powders were drilled from the best material with a handheld microdrill^16^,^66^. Representative shell splinters of processed bivalves and brachiopods, and fragments of most of the belemnite rostra were additionally screened for preservation of the ultrastructure using a FEI Quanta 250 scanning electron microscope (Geological Museum in Copenhagen). Only shells with smooth surfaces (for example, lamellar or fibrous) and no signs of re-crystallization were classified as pristine^67^,^68^. Aliquots of 300–700 μg (Copenhagen) and 150–300 μg (Innsbruck) were reacted in sealed glass vials by adding anhydrous phosphoric acid after removal of atmospheric contaminants with He. Resultant carbon dioxide was analysed for oxygen- and carbon-isotope ratios at the Department of Geosciences and Natural Resource Management, University of Copenhagen (Iso Prime triple collector Isotope Ratio Mass Spectrometer) and at the Institut für Geologie und Paläontologie, University of Innsbruck (ThermoFinnigan DeltaplusXL mass spectrometer; Supplementary Data 1). The reproducibility of the measurements determined by the s.d. of in-house reference materials was 0.18‰ for δ^18^O and 0.08‰ for δ^13^C (2 s.d., *n*=649; ref. 66) in Copenhagen, and better than 0.2‰ (2 s.d.) for δ^18^O and δ^13^C in Innsbruck. Temperatures calculated from the oxygen-isotope values (Fig. 2) are based on ref. 69 assuming a δ^18^O value of −1‰ SMOW for ambient water. The Mn/Ca and Sr/Ca ratios for all processed calcite fossils (Supplementary Data 1) were quantified using the Perkin Elmer Optima 7000 DV ICP-OES at the University of Copenhagen using aliquots from the H_3_PO_4_ treatment remaining after the stable isotope analyses^66^. Reproducibility for Mn/Ca and Sr/Ca ratios, assessed by using different reference materials, was better than 2.8% (2 s.d.) and 2.5% (2 s.d.), respectively (for further details see ref. 66). Mn enrichment and Sr depletion in biogenic calcite were used to identify alteration in samples^2^,^67^. Mn and Sr concentrations in seawater vary temporarily, spatially and, in addition, Sr incorporation in biogenic calcite is variable between taxa and controlled by environmental factors^16^,^57^,^66^,^70^,^71^,^72^,^73^,^74^,^75^. Such potential primary Mn/Ca enrichments and primary variability of Sr/Ca ratios in calcite fossils^2^,^16^,^57^ were taken into account (Supplementary Data 1, and ref. 14).

## Additional information

**How to cite this article:** Korte, C. *et al.* Jurassic climate mode governed by ocean gateway. *Nat. Commun.* 6:10015 doi: 10.1038/ncomms10015 (2015).

## Supplementary Material

Supplementary Information Supplementary Figures 1-2, Supplementary Tables 1-2 and Supplementary References

Supplementary Data 1 Oxygen and carbon isotope data, selected element ratios, location, age, and fossil identification. New and previously published data are included.
