# Supplementary material for: Jurassic climate mode governed by ocean gateway
Source: Nat Commun. 2015 Dec 11;6:10015. doi: 10.1038/ncomms10015 (PMC4682040; doi:10.1038/ncomms10015)
Supplement: Supplementary Information — Supplementary Figures 1-2, Supplementary Tables 1-2 and Supplementary References [file ncomms10015-s1.pdf]

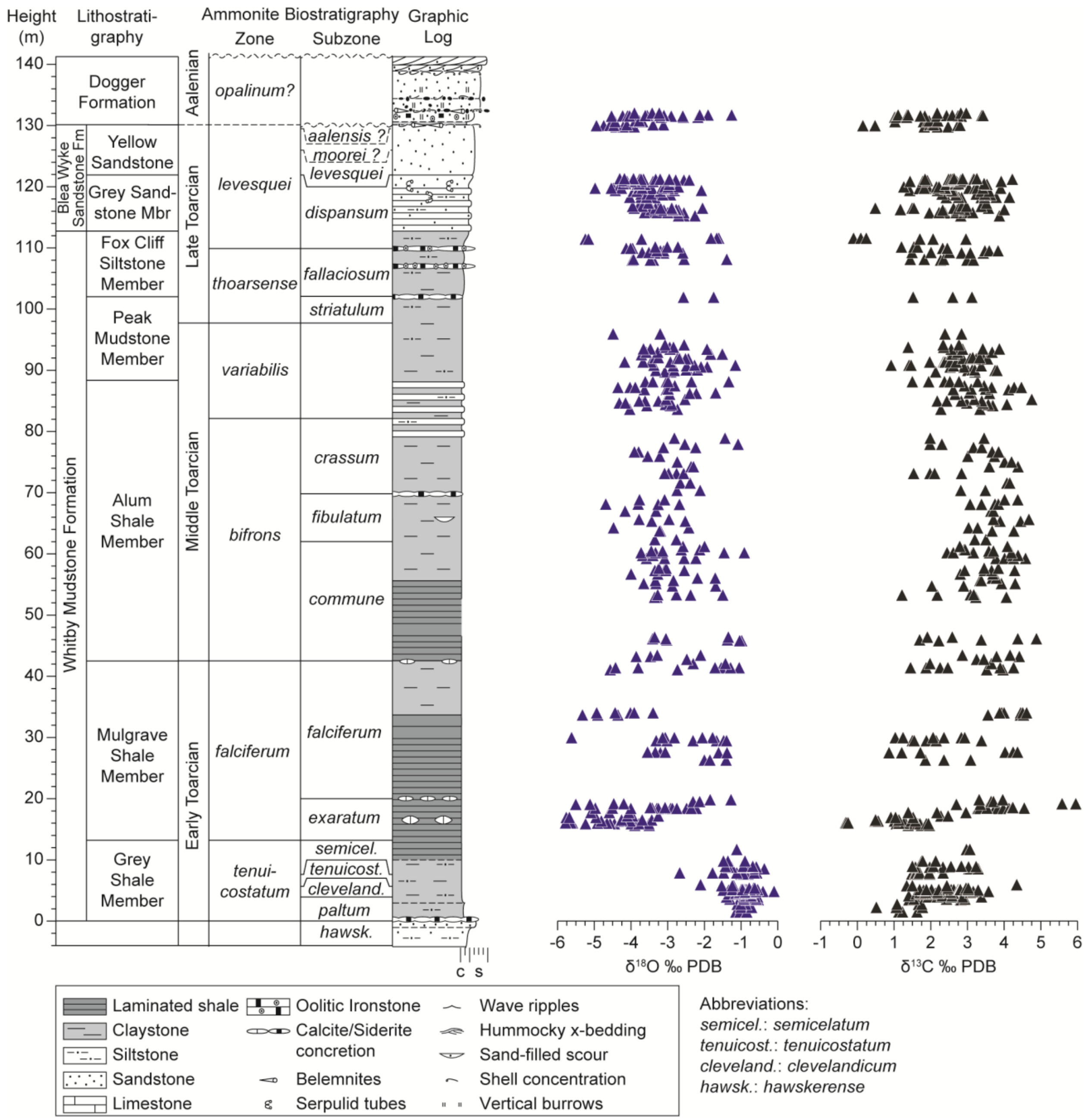

Supplementary Figure 1 | Toarcian to earliest Aalenian lithology, stratigraphy, and oxygen and carbon isotope values from well preserved belemnites from the Yorkshire (NE England) successions at Robin Hood's Bay–Hawsker Bottoms, Saltwick Nab and Ravenscar–Blea Wyke. Note, that the high variability of the oxygen isotope values here is the reflection of seasonal temperature variation[1] and also habitat effects[2,3].

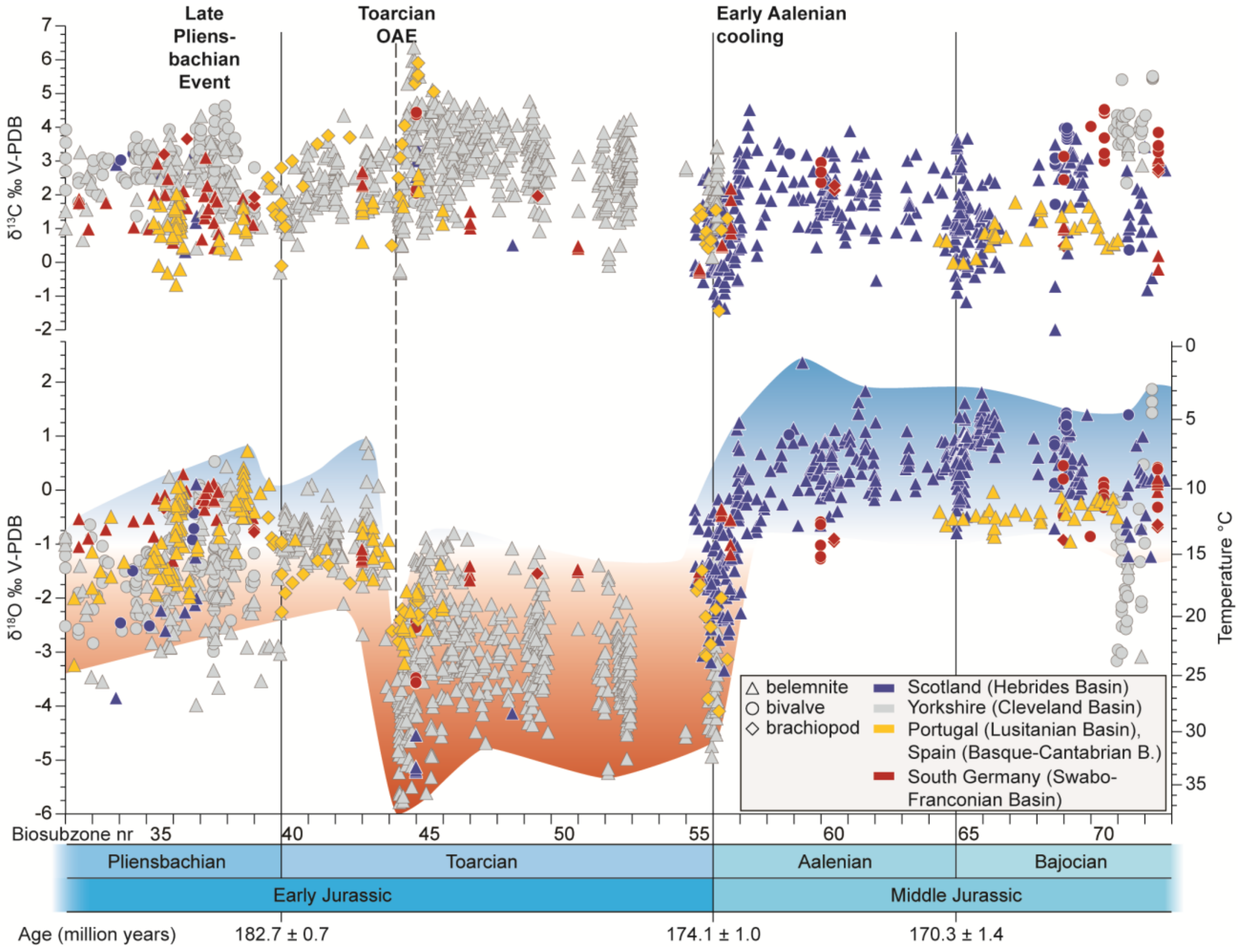

Supplementary Figure 2 │ Pliensbachian to Bajocian carbon isotope record together with oxygen isotopes from the same pristine marine calcite fossils as in Figure 2. The $\delta^{13}C$ data do not follow the abrupt and large magnitude oxygen isotope shift in the earliest Middle Jurassic (see also Figure 3) and show no indication by way of isotopically light values of strong river influx in the Toarcian.

Supplementary Table 1 | Biostratigraphic zonation of regarded time span between the late early Pliensbachian and the late early Bajocian with used biosubzone numbers. Relative positions of samples within the biozones are used and these reflect the highest biostratigraphic accuracy possible for each biozonation of the cited references.

Series	Stage	Biozone nr	Biozone	Biosubzone nr	Biosubzone
Early Jurassic	Pliensbachian	12	*davoei*	32	*maculatum*
Early Jurassic	Pliensbachian	12	*davoei*	33	*capricornus*
Early Jurassic	Pliensbachian	12	*davoei*	34	*figulinum*
Early Jurassic	Pliensbachian	13	*margaritatus*	35	*stokesi*
Early Jurassic	Pliensbachian	13	*margaritatus*	36	*subnodosus*
Early Jurassic	Pliensbachian	13	*margaritatus*	37	*gibbosus*
Early Jurassic	Pliensbachian	14	*spinatum*	38	*apyrenum*
Early Jurassic	Pliensbachian	14	*spinatum*	39	*hawskerense*
Early Jurassic	Toarcian	15	*tenuicostatum*	40	*paltum*
Early Jurassic	Toarcian	15	*tenuicostatum*	41	*clevelandicum*
Early Jurassic	Toarcian	15	*tenuicostatum*	42	*tenuicostatum*
Early Jurassic	Toarcian	15	*tenuicostatum*	43	*semicelatum*
Early Jurassic	Toarcian	16	*falciferum (serpentinum)*	44	*exaratum*
Early Jurassic	Toarcian	16	*falciferum (serpentinum)*	45	*falciferum*
Early Jurassic	Toarcian	17	*bifrons*	46	*commune*
Early Jurassic	Toarcian	17	*bifrons*	47	*fibulatum*
Early Jurassic	Toarcian	17	*bifrons*	48	*crassum*
Early Jurassic	Toarcian	18	*variabilis*	49	
Early Jurassic	Toarcian	19	*thouarsense*	50	*striatulum*
Early Jurassic	Toarcian	19	*thouarsense*	51	*fallaciosum*
Early Jurassic	Toarcian	20	*levesquei*	52	*dispansum*
Early Jurassic	Toarcian	20	*levesquei*	53	*levesquei*
Early Jurassic	Toarcian	20	*levesquei*	54	*moorei*
Early Jurassic	Toarcian	20	*levesquei*	55	*aalensis*
Middle Jurassic	Aalenian	21	*opalinum*	56	
Middle Jurassic	Aalenian	22	*scissum*	57	
Middle Jurassic	Aalenian	23	*murchisonae*	58	*haugi*
Middle Jurassic	Aalenian	23	*murchisonae*	59	*obtusiformis*
Middle Jurassic	Aalenian	23	*murchisonae*	60	*murchisonae*
Middle Jurassic	Aalenian	24	*bradfordensis*	61	*bradfordensis*
Middle Jurassic	Aalenian	24	*bradfordensis*	62	*gigantea*
Middle Jurassic	Aalenian	25	*concavum*	63	*concavum*
Middle Jurassic	Aalenian	25	*concavum*	64	*limitatum*
Middle Jurassic	Bajocian	26	*discites*	65	
Middle Jurassic	Bajocian	27	*ovalis*	66	
Middle Jurassic	Bajocian	28	*laeviuscula*	67	*sayni*
Middle Jurassic	Bajocian	28	*laeviuscula*	68	*trigonalis*
Middle Jurassic	Bajocian	28	*laeviuscula*	69	*laeviuscula*
Middle Jurassic	Bajocian	29	*sauzei*	70	
Middle Jurassic	Bajocian	30	*humphriesianum*	71	*romani*
Middle Jurassic	Bajocian	30	*humphriesianum*	72	*humphriesianum*

Supplementary Table 2 | Coordinates of localities sampled.

Locality	Latitude	Longitude	Region
Robin Hood's Bay	54°24'53" N	0°30'58" W	Yorkshire, NE England
Hawsker Bottoms	54°27'24" N	0°32'20" W	Yorkshire, NE England
Saltwick Nab	54°29'21" N	0°35'18" W	Yorkshire, NE England
Ravenscar–Blea Wyke	54°24'23" N	0°29'25" W	Yorkshire, NE England
Staithes–Brackenberry Wyke	54°33'29" N	0°46'47" W	Yorkshire, NE England
Hundale Point	54°20'22" N	0°25'30" W	Yorkshire, NE England
Bearerraig	57°29'49" N	6°08'40" W	Isle of Skye, Inner Hebrides, Scotland
Druim an Aonaich	57°23'29" N	6°01'11" W	Isle of Raasey, Inner Hebrides, Scotland
Aubach valley near Aselfingen	47°50'49" N	8°28'48" E	Wutach area, SW Germany

Supplementary References

1. Ullmann, C. V., Wiechert, U. & Korte, C. Oxygen isotope fluctuations in a modern North Sea oyster (*Crassostrea gigas*) compared with annual variations in seawater temperature: implications for palaeoclimate studies. *Chem. Geol.* **277**, 160–166 (2010).
2. Korte, C. & Hesselbo, S. P. Shallow marine carbon and oxygen isotope and elemental records indicate icehouse–greenhouse cycles during the Early Jurassic. *Paleoceanography* **26**, PA4219 (2011).
3. Ullmann, C. V., Thibault, N., Ruhl, M., Hesselbo, S. P. & Korte, C. Effect of a Jurassic oceanic anoxic event on belemnite ecology and evolution. *Proc. Natl Acad. Sci. USA* **111,** 10073–10076 (2014).
4. Ullmann, C. V., Hesselbo, S. P. & Korte, C. Tectonic forcing of Early to Middle Jurassic seawater Sr/Ca. *Geology* **41**, 1211–1214 (2013).
5. Jenkyns, H. C., Jones, C. E., Gröcke, D. R., Hesselbo, S. P. & Parkinson, D. N. Chemostratigraphy of the Jurassic System: applications, limitations and implications for palaeoceanography. *J. Geol. Soc. London* **159**, 351–378 (2002).
6. Price, G. D. Carbon-isotope stratigraphy and temperature change during the Early–Middle Jurassic (Toarcian–Aalenian), Raasay, Scotland, UK. *Palaeogeogr. Palaeoclimatol. Palaeoecol.* **285**, 255–263 (2010).
7. Li, Q., McArthur, J. M. & Atkinson, T. C. Lower Jurassic belemnites as indicators of palaeo-temperature. *Palaeogeogr. Palaeoclimatol. Palaeoecol.* **315–316**, 38–45 (2012).
8. McArthur, J. M., Donovan, D. T., Thirlwall, M. F., Fouke, B. W. & Mattey, D. Strontium isotope profile of the early Toarcian (Jurassic) oceanic anoxic event, the duration of ammonite biozones, and belemnite palaeotemperatures. *Earth Planet. Sci. Lett.* **179**, 269–285 (2000).
9. Suan, G., Mattioli, E., Pittet, B., Mailliot, S. & Lécuyer, C. Evidence for major environmental perturbation prior to and during the Toarcian (Early Jurassic) oceanic anoxic event from the Lusitanian Basin, Portugal. *Paleoceanography* **23**, PA1202, (2008).
10. Cresta, S. *et al.* The Global Boundary Stratotype Section and Point (GSSP) of the Toarcian-Aalenian Boundary (Lower-Middle Jurassic). *Episodes* **24(3)**, 166–175 (2001).
11. Rosales, I., Quesada, S. & Robles, S. Paleotemperature variations of Early Jurassic seawater recorded in geochemical trends of belemnites from the Basque-Cantabrian basin, northern Spain. *Palaeogeogr. Palaeoclimatol. Palaeoecol.* **203**, 253–275 (2004).
12. Rosales, I., Quesada, S. & Robles, S. Primary and diagenetic isotopic signals in fossils and hemipelagic carbonates: the Lower Jurassic of northern Spain. *Sedimentology* **48**, 1149–1169 (2001).
