# Supplementary material for: Jurassic climate mode governed by ocean gateway
Source: Nat Commun. 2015 Dec 11;6:10015. doi: 10.1038/ncomms10015 (PMC4682040; doi:10.1038/ncomms10015)
Supplement: Supplementary Data 1 — Oxygen and carbon isotope data, selected element ratios, location, age, and fossil identification. New and previously published data are included. [file ncomms10015-s2.pdf]

Supplementary Dataset | Oxygen and carbon isotope data, selected element ratios, location, age, and fossil identification. New and previously published data are included.

**sample** stratigraphic marker locality region	**stage**	**zone**	**subzone**	**section heights**	**subzone heights**	**material**	**species**	**remark**	**$\delta^{13}C$ PDB (‰)**	**$\delta^{18}O$ PDB (‰)**	**Iso Lab**	**Mg/Ca mmol/ mol**	**Mn/Ca µmol/ mol**	**Sr/Ca mmol/ mol**	**reference**
Scotland															
our data (including new and literature data)															
Druim an Aonaich (Raasay)															
base *capricornus* sz	Pli	*davoei*	*capricornus*	3830	33,000			approximated							this study
D-Aon 40	Pli	*davoei*	*capricornus*	4045	33,878	belemnite			2,90	-3,85	CPH	15,1	205	2,23	this study
base *figulinum* sz	Pli	*davoei*	*figulinum*	4075	34,000										this study
D-Aon 41	Pli	*davoei*	*figulinum*	4080	34,045	pectinid			3,03	-2,46	CPH	2,3	1241	1,12	this study
D-Aon 42	Pli	*davoei*	*figulinum*	4130	34,500	bivalve unident.			3,22	-1,50	CPH	3,7	100	0,66	this study
base *stokesi* sz	Pli	*margaritatus*	*stokesi*	4185	35,000										this study
D-Aon 47	Pli	*margaritatus*	*stokesi*	4290	35,117	oyster			3,26	-2,52	CPH	4,0	122	0,59	this study
D-Aon 63	Pli	*margaritatus*	*stokesi*	4670	35,542	belemnite			2,61	-2,23	CPH	9,7	24	1,90	this study
D-Aon 72	Pli	*margaritatus*	*stokesi*	4820	35,709	belemnite			2,44	-2,60	CPH	12,8	2	2,10	this study
D-Aon 70	Pli	*margaritatus*	*stokesi*	5030	35,944	belemnite		altered	0,16	-1,09	CPH	9,1	88	1,50	this study
D-Aon 71	Pli	*margaritatus*	*stokesi*	5050	35,966	pectinid			2,42	-1,70	CPH	3,2	177	0,91	this study
base *subnodosus* sz	Pli	*margaritatus*	*subnodosus*	5080	36,000			approximated							this study
D-Aon 49	Pli	*margaritatus*	*subnodosus*	6010	36,433	belemnite			0,32	-2,26	CPH	13,9	24	1,80	this study
D-Aon 77	Pli	*margaritatus*	*subnodosus*	6615	36,714	bivalve unident.			2,57	-0,92	CPH	10,3	381	1,16	this study
D-Aon 80	Pli	*margaritatus*	*subnodosus*	6730	36,767	bivalve unident.			2,87	-0,43	CPH	5,8	683	1,10	this study
D-Aon 88	Pli	*margaritatus*	*subnodosus*	6730	36,767	oyster			3,12	-0,71	CPH	2,9	231	0,63	this study
D-Aon 91	Pli	*margaritatus*	*subnodosus*	6815	36,807	belemnite			2,64	-2,12	CPH	10,2	34	1,72	this study
D-Aon 82	Pli	*margaritatus*	*subnodosus*	6850	36,823	belemnite			1,18	-1,24	CPH	11,6	73	1,51	this study
D-Aon 83	Pli	*margaritatus*	*subnodosus*	6915	36,853	belemnite			3,39	0,11	CPH	14,0	13	2,23	this study
D-Aon 79	Pli	*margaritatus*	*subnodosus*	6945	36,867	belemnite			1,38	-2,00	CPH	12,2	103	1,42	this study
base *gibbosus* sz	Pli			7230	37,000			approximated							this study
Bearreraig (Isle of Sky)															
base *aalensis* sz	Toa	*levesquei*	*aalensis*	-375	55,000										this study
Bea 92	Toa	*levesquei*	*aalensis*	-90	55,345	belemnite			-0,23	-0,36	CPH		10	1,49	this study (element data Ullmann et al. 2013[4])
Bea 92	Toa	*levesquei*	*aalensis*	-90	55,345	belemnite			-0,07	-0,53	CPH		8	1,72	this study (element data Ullmann et al. 2013[4])
Bea 94	Toa	*levesquei*	*aalensis*	-70	55,370	belemnite			1,06	-1,69	CPH		17	1,73	this study (element data Ullmann et al. 2013[4])
Bea 94	Toa	*levesquei*	*aalensis*	-70	55,370	belemnite			0,83	-1,41	CPH		10	1,83	this study (element data Ullmann et al. 2013[4])
Bea 93	Toa	*levesquei*	*aalensis*	30	55,491	belemnite			-0,13	-0,71	CPH		11	1,78	this study (element data Ullmann et al. 2013[4])
Bea 93	Toa	*levesquei*	*aalensis*	30	55,491	belemnite			-0,18	-0,72	CPH		11	1,72	this study (element data Ullmann et al. 2013[4])
Bea 97	Toa	*levesquei*	*aalensis*	140	55,624	belemnite			1,71	-1,40	CPH		16	1,75	this study (element data Ullmann et al. 2013[4])
Bea 97	Toa	*levesquei*	*aalensis*	140	55,624	belemnite			2,48	-3,06	CPH		9	1,68	this study (element data Ullmann et al. 2013[4])
B1	Toa	*levesquei*	*aalensis*	150	55,636	belemnite			-0,57	-1,46					Jenkyns et al. 2002[5]
Bea 104	Toa	*levesquei*	*aalensis*	300	55,818	belemnite			1,54	-1,83	CPH		13	1,98	this study (element data Ullmann et al. 2013[4])
Bea 104	Toa	*levesquei*	*aalensis*	300	55,818	belemnite			1,52	-1,03	CPH		10	2,04	this study (element data Ullmann et al. 2013[4])
Bea 105	Toa	*levesquei*	*aalensis*	300	55,818	belemnite			1,24	-1,81	CPH		10	1,78	this study (element data Ullmann et al. 2013[4])
Bea 105	Toa	*levesquei*	*aalensis*	300	55,818	belemnite			1,39	-1,63	CPH		8	1,77	this study (element data Ullmann et al. 2013[4])
Bea 100	Toa	*levesquei*	*aalensis*	330	55,855	belemnite			2,38	-2,78	CPH		8	1,80	this study (element data Ullmann et al. 2013[4])
Bea 100	Toa	*levesquei*	*aalensis*	330	55,855	belemnite			1,13	-1,33	CPH		9	1,83	this study (element data Ullmann et al. 2013[4])
Bea 102	Toa	*levesquei*	*aalensis*	330	55,855	belemnite			0,67	-0,75	CPH		9	1,64	this study (element data Ullmann et al. 2013[4])
Bea 102	Toa	*levesquei*	*aalensis*	330	55,855	belemnite			0,79	-0,54	CPH		10	1,64	this study (element data Ullmann et al. 2013[4])
Bea 109	Toa	*levesquei*	*aalensis*	355	55,885	belemnite			0,99	-2,35	CPH		17	1,68	this study (element data Ullmann et al. 2013[4])
Bea 109	Toa	*levesquei*	*aalensis*	355	55,885	belemnite			-0,27	-1,62	CPH		10	1,56	this study (element data Ullmann et al. 2013[4])
Bea 110	Toa	*levesquei*	*aalensis*	400	55,939	belemnite			1,55	-3,18	CPH		13	1,65	this study (element data Ullmann et al. 2013[4])
Bea 110	Toa	*levesquei*	*aalensis*	400	55,939	belemnite			1,89	-2,57	CPH		14	1,79	this study (element data Ullmann et al. 2013[4])
Bea 113	Toa	*levesquei*	*aalensis*	415	55,958	belemnite			0,09	-1,99	CPH		7	1,69	this study (element data Ullmann et al. 2013[4])
Bea 113	Toa	*levesquei*	*aalensis*	415	55,958	belemnite			2,00	-1,78	CPH		7	1,76	this study (element data Ullmann et al. 2013[4])
Bea 114	Toa	*levesquei*	*aalensis*	415	55,958	belemnite			0,75	-1,51	CPH		7	1,88	this study (element data Ullmann et al. 2013[4])
Bea 114	Toa	*levesquei*	*aalensis*	415	55,958	belemnite			0,77	-1,60	CPH		11	1,86	this study (element data Ullmann et al. 2013[4])
base Aalenian	Aal	*opalinum*	-	450	56,000			approximated							this study
base *opalinum* z	Aal	*opalinum*	-	450	56,000			approximated							this study
Bea 111	Aal	*opalinum*	-	470	56,029	belemnite			0,57	-1,66	CPH		10	1,91	this study (element data Ullmann et al. 2013[4])
Bea 111	Aal	*opalinum*	-	470	56,029	belemnite			0,89	-1,11	CPH		10	1,95	this study (element data Ullmann et al. 2013[4])
Bea 85	Aal	*opalinum*	-	485	56,050	belemnite			-0,92	-0,42	CPH		11	1,40	this study (element data Ullmann et al. 2013[4])
Bea 85	Aal	*opalinum*	-	485	56,050	belemnite			-0,07	-0,40	CPH		8	1,42	this study (element data Ullmann et al. 2013[4])
B2	Aal	*opalinum*	-	525	56,107	belemnite			1,28	-1,87					Jenkyns et al. 2002[5]
Bea 90	Aal	*opalinum*	-	525	56,107	belemnite			2,47	-2,28	CPH		14	1,86	this study (element data Ullmann et al. 2013[4])
Bea 90	Aal	*opalinum*	-	525	56,107	belemnite			1,77	-2,08	CPH		15	1,91	this study (element data Ullmann et al. 2013[4])
Bea 91	Aal	*opalinum*	-	535	56,121	belemnite			0,56	-1,66	CPH		19	1,70	this study (element data Ullmann et al. 2013[4])

Bea 91	Aal	*opalinum*	-	535	56,121	belemnite		-0,17	-0,99	CPH		19	1,60	this study (element data Ullmann et al. 2013[4])
Bea 84	Aal	*opalinum*	-	565	56,164	belemnite		-0,23	-0,78	CPH		8	1,53	this study (element data Ullmann et al. 2013[4])
Bea 84	Aal	*opalinum*	-	565	56,164	belemnite		-1,14	-2,23	CPH		13	1,54	this study (element data Ullmann et al. 2013[4])
Bea 89	Aal	*opalinum*	-	595	56,207	belemnite		1,63	-1,90	CPH		11	2,08	this study (element data Ullmann et al. 2013[4])
Bea 89	Aal	*opalinum*	-	595	56,207	belemnite		1,02	-2,08	CPH		13	2,16	this study (element data Ullmann et al. 2013[4])
Bea 88	Aal	*opalinum*	-	600	56,214	belemnite		-0,54	-0,16	CPH		17	1,74	this study (element data Ullmann et al. 2013[4])
Bea 88	Aal	*opalinum*	-	600	56,214	belemnite		-0,20	-0,47	CPH		16	1,54	this study (element data Ullmann et al. 2013[4])
Bea 82	Aal	*opalinum*	-	615	56,236	belemnite		1,87	-2,77	CPH		40	1,92	this study (element data Ullmann et al. 2013[4])
Bea 82	Aal	*opalinum*	-	615	56,236	belemnite	altered	0,61	-4,10	CPH		30	1,70	this study (element data Ullmann et al. 2013[4])
Bea 77	Aal	*opalinum*	-	670	56,314	belemnite		2,12	-1,19	CPH		8	1,72	this study (element data Ullmann et al. 2013[4])
Bea 77	Aal	*opalinum*	-	670	56,314	belemnite		1,57	-0,79	CPH		8	1,71	this study (element data Ullmann et al. 2013[4])
Bea 78	Aal	*opalinum*	-	695	56,350	belemnite	altered	1,27	-4,13	CPH		15	1,97	this study (element data Ullmann et al. 2013[4])
Bea 78	Aal	*opalinum*	-	695	56,350	belemnite	altered	0,43	-4,06	CPH		24	2,00	this study (element data Ullmann et al. 2013[4])
Bea 80	Aal	*opalinum*	-	720	56,386	belemnite		2,10	-0,49	CPH		12	1,79	this study (element data Ullmann et al. 2013[4])
Bea 80	Aal	*opalinum*	-	720	56,386	belemnite		0,91	-0,53	CPH		12	1,51	this study (element data Ullmann et al. 2013[4])
Bea 72	Aal	*opalinum*	-	750	56,429	belemnite		1,56	-1,34	CPH		9	1,93	this study (element data Ullmann et al. 2013[4])
Bea 72	Aal	*opalinum*	-	750	56,429	belemnite		1,22	-1,28	CPH		12	1,85	this study (element data Ullmann et al. 2013[4])
Bea 73	Aal	*opalinum*	-	750	56,429	belemnite		-0,57	-0,59	CPH		15	1,79	this study (element data Ullmann et al. 2013[4])
Bea 73	Aal	*opalinum*	-	750	56,429	belemnite		-0,70	-0,60	CPH		13	1,85	this study (element data Ullmann et al. 2013[4])
Bea 74	Aal	*opalinum*	-	750	56,429	belemnite		-0,90	-0,69	CPH		16	1,64	this study (element data Ullmann et al. 2013[4])
Bea 74	Aal	*opalinum*	-	750	56,429	belemnite		-1,35	-0,69	CPH		12	1,63	this study (element data Ullmann et al. 2013[4])
Bea 81	Aal	*opalinum*	-	750	56,429	belemnite		1,35	-2,40	CPH		23	1,87	this study (element data Ullmann et al. 2013[4])
Bea 81	Aal	*opalinum*	-	750	56,429	belemnite		1,17	-3,34	CPH		28	1,80	this study (element data Ullmann et al. 2013[4])
B3a	Aal	*opalinum*	-	810	56,514	belemnite		0,76	-1,63					Jenkyns et al. 2002[5]
B3b	Aal	*opalinum*	-	810	56,514	belemnite		1,96	-1,99					Jenkyns et al. 2002[5]
Bea 125	Aal	*opalinum*	-	855	56,579	belemnite		2,10	-2,66	CPH		14	2,01	this study (element data Ullmann et al. 2013[4])
Bea 125	Aal	*opalinum*	-	855	56,579	belemnite		2,25	-2,38	CPH		11	1,86	this study (element data Ullmann et al. 2013[4])
Bea 126	Aal	*opalinum*	-	865	56,593	belemnite		-0,20	-0,78	CPH		14	1,62	this study (element data Ullmann et al. 2013[4])
Bea 126	Aal	*opalinum*	-	865	56,593	belemnite		-0,33	-0,59	CPH		17	1,58	this study (element data Ullmann et al. 2013[4])
Bea 124	Aal	*opalinum*	-	885	56,621	belemnite		0,22	-0,15	CPH		12	1,60	this study (element data Ullmann et al. 2013[4])
Bea 124	Aal	*opalinum*	-	885	56,621	belemnite		1,09	-2,58	CPH		10	1,76	this study (element data Ullmann et al. 2013[4])
Bear 238	Aal	*opalinum*	-	910	56,657	belemnite	altered	3,41	-4,99	CPH	15,3	21	2,45	this study
Bea 120	Aal	*opalinum*	-	1010	56,800	belemnite		1,62	-1,25	CPH		10	1,69	this study (element data Ullmann et al. 2013[4])
Bea 120	Aal	*opalinum*	-	1010	56,800	belemnite		2,17	-1,93	CPH		6	1,81	this study (element data Ullmann et al. 2013[4])
Bea 121	Aal	*opalinum*	-	1015	56,807	belemnite		0,79	-1,10	CPH		25	1,50	this study (element data Ullmann et al. 2013[4])
Bea 121	Aal	*opalinum*	-	1015	56,807	belemnite		0,91	-0,97	CPH		15	1,61	this study (element data Ullmann et al. 2013[4])
Bea 122	Aal	*opalinum*	-	1015	56,807	belemnite		0,17	-0,37	CPH		22	1,64	this study (element data Ullmann et al. 2013[4])
Bea 122	Aal	*opalinum*	-	1015	56,807	belemnite		0,02	-0,53	CPH		22	1,62	this study (element data Ullmann et al. 2013[4])
Bear 237	Aal	*opalinum*	-	1020	56,814	belemnite		0,84	-1,49	CPH	11,5	3	1,83	this study
Bea 117	Aal	*opalinum*	-	1080	56,900	belemnite	altered	0,80	-4,49	CPH		10	1,79	this study (element data Ullmann et al. 2013[4])
Bea 117	Aal	*opalinum*	-	1080	56,900	belemnite	altered	0,13	-5,16	CPH		13	1,63	this study (element data Ullmann et al. 2013[4])
Bear 234	Aal	*opalinum*	-	1090	56,914	belemnite		-0,52	-1,25	CPH	10,3	22	1,66	this study
Bear 234	Aal	*opalinum*	-	1090	56,914	belemnite		-0,49	-1,04	CPH	10,0	29	1,61	this study
B4	Aal	*opalinum*	-	1110	56,943	belemnite		0,41	-0,22					Jenkyns et al. 2002[5]
Bea 116	Aal	*opalinum*	-	1125	56,964	belemnite		0,97	-0,55	CPH		24	1,89	this study (element data Ullmann et al. 2013[4])
Bea 116	Aal	*opalinum*	-	1125	56,964	belemnite		0,93	-0,49	CPH		11	1,92	this study (element data Ullmann et al. 2013[4])
base *scissum* z	Aal	*scissum*	-	1150	57,000		approximated							this study
Bea 118	Aal	*scissum*	-	1165	57,043	belemnite		0,46	-2,27	CPH		22	1,52	this study (element data Ullmann et al. 2013[4])
Bea 118	Aal	*scissum*	-	1165	57,043	belemnite		1,05	-1,88	CPH		10	1,64	this study (element data Ullmann et al. 2013[4])
Bear 236	Aal	*scissum*	-	1370	57,629	belemnite		0,21	-0,77	CPH	11,3	9	1,68	this study
Bear 236	Aal	*scissum*	-	1370	57,629	belemnite		0,52	-0,67	CPH	9,7	39	1,63	this study
Bear 235	Aal	*scissum*	-	1470	57,914	belemnite		1,42	-0,35	CPH	9,5	13	1,79	this study
Bear 235	Aal	*scissum*	-	1470	57,914	belemnite		2,33	-0,28	CPH	9,6	45	1,89	this study
base *murchisonnae* z	Aal	*murchisonnae*	*haugi*	1500	58,000		approximated							this study
base *haugi* sz	Aal	*murchisonnae*	*haugi*	1500	58,000		approximated							this study
B5	Aal	*murchisonnae*	*haugi*	1540	58,444	belemnite		3,55	0,49					Jenkyns et al. 2002[5]
B6	Aal	*murchisonnae*	*haugi*	1550	58,556	belemnite		3,37	0,88					Jenkyns et al. 2002[5]
Bear 208	Aal	*murchisonnae*	*haugi*	1550	58,556	belemnite		2,25	0,65	CPH	9,4	17	1,70	this study
Bear 208	Aal	*murchisonnae*	*haugi*	1550	58,556	belemnite		2,23	0,44	CPH	9,7	60	1,75	this study
Bear 231	Aal	*murchisonnae*	*haugi*	1575	58,833	oyster		3,21	1,02	CPH	1,2	38	0,63	this study
base *obtusiformis* sz	Aal	*murchisonnae*	*obtusiformis*	1590	59,000									this study
B8	Aal	*murchisonnae*	*obtusiformis*	1590	59,000	belemnite		2,40	1,14					Jenkyns et al. 2002[5]
Bear 216 A	Aal	*murchisonnae*	*obtusiformis*	1605	59,167	belemnite		1,08	0,00		11,0	113	1,71	this study
Bear 216 B	Aal	*murchisonnae*	*obtusiformis*	1605	59,167	belemnite		1,94	-0,65	CPH	10,8	94	1,76	this study
Bear 216 C	Aal	*murchisonnae*	*obtusiformis*	1605	59,167	belemnite	altered	-0,15	-2,65	CPH	9,4	370	1,53	this study
Bear 210	Aal	*murchisonnae*	*obtusiformis*	1620	59,333	belemnite		2,41	2,34	CPH	8,6	4	1,57	this study
Bear 210	Aal	*murchisonnae*	*obtusiformis*	1620	59,333	belemnite		1,86	0,93	CPH	9,4	5	1,72	this study
Bear 209 A	Aal	*murchisonnae*	*obtusiformis*	1645	59,611	belemnite		1,94	0,38	CPH	9,4	28	2,01	this study
Bear 209 B	Aal	*murchisonnae*	*obtusiformis*	1645	59,611	belemnite		1,06	0,30	CPH	7,7	17	1,59	this study
Bear 209 C	Aal	*murchisonnae*	*obtusiformis*	1645	59,611	belemnite		1,04	0,01	CPH	9,1	10	1,56	this study
B10	Aal	*murchisonnae*	*obtusiformis*	1655	59,722	belemnite		2,71	0,45					Jenkyns et al. 2002[5]
Bear 211 B	Aal	*murchisonnae*	*obtusiformis*	1665	59,833	belemnite		1,03	0,28	CPH	9,3	25	1,69	this study
Bear 211 C	Aal	*murchisonnae*	*obtusiformis*	1665	59,833	belemnite		1,21	0,57	CPH	9,1	33	1,66	this study
Bear 211A	Aal	*murchisonnae*	*obtusiformis*	1665	59,833	belemnite		0,46	0,79	CPH	9,1	12	1,68	this study
base *murchisonae* sz	Aal	*murchisonnae*	*murchisonnae*	1680	60,000									this study
Bea 128	Aal	*murchisonnae*	*murchisonnae*	1680	60,000	belemnite		1,60	0,13	CPH		9	1,49	this study (element data Ullmann et al. 2013[4])
Bea 128	Aal	*murchisonnae*	*murchisonnae*	1680	60,000	belemnite		1,57	0,41	CPH		11	1,54	this study (element data Ullmann et al. 2013[4])
Bear 213 A	Aal	*murchisonnae*	*murchisonnae*	1680	60,000	belemnite		1,70	-0,14	CPH	9,1	76	1,80	this study
Bear 213 B	Aal	*murchisonnae*	*murchisonnae*	1680	60,000	belemnite		1,80	-0,65	CPH	10,2	111	1,78	this study

Bear 213 C	Aal	*murchisonnae*	*murchisonnae*	1680	60,000	belemnite	altered	0,97	-1,04	CPH	12,9	158	1,82	this study
Bea 130	Aal	*murchisonnae*	*murchisonnae*	1690	60,067	belemnite		1,43	0,21	CPH		7	1,62	this study (element data Ullmann et al. 2013[4])
Bea 130	Aal	*murchisonnae*	*murchisonnae*	1690	60,067	belemnite		2,16	0,66	CPH		6	1,64	this study (element data Ullmann et al. 2013[4])
Bea 136	Aal	*murchisonnae*	*murchisonnae*	1705	60,167	belemnite		1,83	0,92	CPH		5	1,64	this study (element data Ullmann et al. 2013[4])
Bea 136	Aal	*murchisonnae*	*murchisonnae*	1705	60,167	belemnite		2,58	0,89	CPH		9	1,73	this study (element data Ullmann et al. 2013[4])
Bear b 101	Aal	*murchisonnae*	*murchisonnae*	1710	60,200	belemnite		3,27	-0,02	CPH	9,1	24	1,71	this study
Bea 131	Aal	*murchisonnae*	*murchisonnae*	1715	60,233	belemnite		1,97	-0,06	CPH		8	1,62	this study (element data Ullmann et al. 2013[4])
Bea 131	Aal	*murchisonnae*	*murchisonnae*	1715	60,233	belemnite		2,34	0,00	CPH		11	1,68	this study (element data Ullmann et al. 2013[4])
Bear 204	Aal	*murchisonnae*	*murchisonnae*	1730	60,333	oyster	altered	1,13	-3,57	CPH	5,5	475	1,24	this study
Bear 205	Aal	*murchisonnae*	*murchisonnae*	1730	60,333	belemnite		1,71	0,48	CPH	8,2	7	1,64	this study
Bear 205	Aal	*murchisonnae*	*murchisonnae*	1730	60,333	belemnite		1,50	0,45	CPH	9,0	12	1,52	this study
Bear 215 A	Aal	*murchisonnae*	*murchisonnae*	1730	60,333	belemnite		2,07	0,08	CPH	9,5	10	1,92	this study
Bear 215 B	Aal	*murchisonnae*	*murchisonnae*	1730	60,333	belemnite		2,35	-0,09	CPH	9,7	22	1,94	this study
Bear 215 C	Aal	*murchisonnae*	*murchisonnae*	1730	60,333	belemnite		1,60	-0,48	CPH	9,3	9	1,95	this study
Bear 214	Aal	*murchisonnae*	*murchisonnae*	1755	60,500	belemnite		3,17	0,84	CPH	10,0	3	1,85	this study
Bear 214	Aal	*murchisonnae*	*murchisonnae*	1755	60,500	belemnite		2,84	0,49	CPH	9,9	7	1,82	this study
Bear b 102	Aal	*murchisonnae*	*murchisonnae*	1760	60,533	belemnite		2,46	1,11	CPH	17,4	12	1,68	this study
Bear 206 A	Aal	*murchisonnae*	*murchisonnae*	1775	60,633	belemnite		1,53	0,15	CPH	8,6	8	1,71	this study
Bear 206 B	Aal	*murchisonnae*	*murchisonnae*	1775	60,633	belemnite		1,36	0,21	CPH	8,8	10	1,73	this study
Bear 206 C	Aal	*murchisonnae*	*murchisonnae*	1775	60,633	belemnite		1,28	-0,39	CPH	10,3	15	1,88	this study
Bear 201	Aal	*murchisonnae*	*murchisonnae*	1795	60,767	belemnite		1,41	0,19	CPH	8,5	6	1,77	this study
Bear 201	Aal	*murchisonnae*	*murchisonnae*	1795	60,767	belemnite		1,04	-0,12	CPH	11,2	24	1,74	this study
Bea 132	Aal	*murchisonnae*	*murchisonnae*	1815	60,900	belemnite		0,83	0,95	CPH		12	1,26	this study (element data Ullmann et al. 2013[4])
Bea 132	Aal	*murchisonnae*	*murchisonnae*	1815	60,900	belemnite		0,60	0,88	CPH		14	1,31	this study (element data Ullmann et al. 2013[4])
base *bradfordensis* z	Aal	*bradfordensis*	*bradfordensis*	1830	61,000									this study
base *bradfordensis* sz	Aal	*bradfordensis*	*bradfordensis*	1830	61,000									this study
Bear b 103	Aal	*bradfordensis*	*bradfordensis*	1840	61,074	belemnite		3,89	1,04	CPH	9,2	5	1,82	this study
Bear 203	Aal	*bradfordensis*	*bradfordensis*	1840	61,074	belemnite		1,17	0,18	CPH	8,2	60	1,57	this study
Bear 203	Aal	*bradfordensis*	*bradfordensis*	1840	61,074	belemnite		2,15	0,34	CPH	7,8	65	1,59	this study
Bea 134	Aal	*bradfordensis*	*bradfordensis*	1845	61,111	belemnite		1,70	0,91	CPH		16	1,72	this study (element data Ullmann et al. 2013[4])
Bea 134	Aal	*bradfordensis*	*bradfordensis*	1845	61,111	belemnite		2,16	0,74	CPH		15	1,91	this study (element data Ullmann et al. 2013[4])
Bear b 104	Aal	*bradfordensis*	*bradfordensis*	1875	61,333	belemnite		3,16	0,58	CPH	7,8	18	1,60	this study
Bea 139	Aal	*bradfordensis*	*bradfordensis*	1885	61,407	belemnite		2,61	1,42	CPH		9	1,91	this study (element data Ullmann et al. 2013[4])
Bea 139	Aal	*bradfordensis*	*bradfordensis*	1885	61,407	belemnite		2,02	1,63	CPH		8	1,99	this study (element data Ullmann et al. 2013[4])
Bear b 105	Aal	*bradfordensis*	*bradfordensis*	1900	61,519	belemnite		2,10	0,56	CPH	7,7	14	1,53	this study
Bear b 105	Aal	*bradfordensis*	*bradfordensis*	1900	61,519	belemnite		1,73	0,31	CPH	8,6	37	1,41	this study
Bear b 106	Aal	*bradfordensis*	*bradfordensis*	1910	61,593	belemnite		3,25	0,49	CPH	10,2	8	1,65	this study
Bea 141	Aal	*bradfordensis*	*bradfordensis*	1920	61,667	belemnite		1,01	0,76	CPH		5	1,61	this study (element data Ullmann et al. 2013[4])
Bea 141	Aal	*bradfordensis*	*bradfordensis*	1920	61,667	belemnite		0,97	0,87	CPH		3	1,68	this study (element data Ullmann et al. 2013[4])
Bea 142	Aal	*bradfordensis*	*bradfordensis*	1920	61,667	belemnite		2,56	1,84	CPH		11	1,81	this study (element data Ullmann et al. 2013[4])
Bea 142	Aal	*bradfordensis*	*bradfordensis*	1920	61,667	belemnite		2,45	1,09	CPH		10	2,04	this study (element data Ullmann et al. 2013[4])
Bear b 107	Aal	*bradfordensis*	*bradfordensis*	1937	61,793	belemnite		2,38	-0,04	CPH	8,7	8	1,55	this study
Bear 202 A	Aal	*bradfordensis*	*bradfordensis*	1960	61,963	belemnite		1,81	-0,08	CPH	10,2	16	1,86	this study
Bear 202 B	Aal	*bradfordensis*	*bradfordensis*	1960	61,963	belemnite		1,71	0,69	CPH	9,4	36	1,75	this study
Bear 202 C	Aal	*bradfordensis*	*bradfordensis*	1960	61,963	belemnite		0,74	0,51	CPH	9,2	28	1,85	this study
base *gigantea* sz	Aal	*bradfordensis*	*gigantea*	1965	62,000									
Bea 144	Aal	*bradfordensis*	*gigantea*	1965	62,000	belemnite		2,16	0,12	CPH		7	1,82	this study (element data Ullmann et al. 2013[4])
Bea 144	Aal	*bradfordensis*	*gigantea*	1965	62,000	belemnite		1,83	-0,18	CPH		11	1,72	this study (element data Ullmann et al. 2013[4])
Bea 144	Aal	*bradfordensis*	*gigantea*	1965	62,000	oyster	altered	-0,74	-0,75	CPH		389	1,27	this study
Bea 144	Aal	*bradfordensis*	*gigantea*	1965	62,000	oyster	altered	-0,94	0,13	CPH		265	1,06	this study
B11	Aal	*bradfordensis*	*gigantea*	1965	62,000	belemnite		2,07	0,29					Jenkyns et al. 2002[5]
Bear b 108	Aal	*bradfordensis*	*gigantea*	1980	62,045	belemnite		-0,52	1,25	CPH	16,2	9	1,45	this study
Bear 246	Aal	*bradfordensis*	*gigantea*	1980	62,045	belemnite		2,24	-0,01	CPH	11,8	14	1,92	this study
Bear 246	Aal	*bradfordensis*	*gigantea*	1980	62,045	belemnite		2,63	0,19	CPH	12,0	10	2,06	this study
Bear 300	Aal	*bradfordensis*	*gigantea*	1980	62,045	belemnite		0,95	0,12	CPH	6,2	20	1,42	this study
Bear 301	Aal	*bradfordensis*	*gigantea*	2230	62,791	belemnite		0,90	-0,08	CPH	6,0	6	1,27	this study
base *concavum* z	Aal	*concavum*	*concavum*	2300	63,000									this study
base *concavum* sz	Aal	*concavum*	*concavum*	2300	63,000									this study
Bear 303	Aal	*concavum*	*concavum*	2425	63,160	belemnite		2,24	0,39	CPH	6,3	10	1,47	this study
Bear 304	Aal	*concavum*	*concavum*	2425	63,160	belemnite		1,31	0,59	CPH	8,3	0	1,64	this study
Bear 305	Aal	*concavum*	*concavum*	2460	63,205	belemnite		3,02	1,09	CPH	7,9	3	1,87	this study
Bear 306	Aal	*concavum*	*concavum*	2460	63,205	belemnite	altered	0,13	-1,17	CPH	11,1	292	1,46	this study
B18	Aal	*concavum*	*concavum*	2515	63,276	belemnite		1,77	0,52					Jenkyns et al. 2002[5]
Bear 307	Aal	*concavum*	*concavum*	2710	63,526	belemnite		3,21	0,12	CPH	9,5	5	1,80	this study
Bear 308	Aal	*concavum*	*concavum*	2710	63,526	belemnite		1,48	-0,01	CPH	8,2	7	1,65	this study
Bear 309	Aal	*concavum*	*concavum*	2710	63,526	belemnite		0,81	-0,01	CPH	6,4	71	1,35	this study
base *limitatum* sz	Aal	*concavum*	*limitatum*	3080	64,000		approximated							this study
B16	Aal	*concavum*	*limitatum*	3585	64,411	belemnite		2,17	0,31					Jenkyns et al. 2002[5]
B17b	Aal	*concavum*	*limitatum*	3690	64,496	belemnite		2,08	0,90					Jenkyns et al. 2002[5]
Bear 21 ABBconcr -595	Aal	*concavum*	*limitatum*	3840	64,618	belemnite		0,70	0,90	CPH	9,7	6	1,58	this study
Bear 21 ABBconcr -595	Aal	*concavum*	*limitatum*	3840	64,618	belemnite		0,38	0,40	CPH	9,4	15	1,52	this study
Bear 19 ABBconcr -515	Aal	*concavum*	*limitatum*	3920	64,683	belemnite		1,16	0,69	CPH	7,0	20	1,52	this study
Bear 19 ABBconcr -515	Aal	*concavum*	*limitatum*	3920	64,683	belemnite		1,26	0,63	CPH	7,1	16	1,53	this study
Bear 06 ABBconcr -155	Aal	*concavum*	*limitatum*	4280	64,976	belemnite		1,08	0,43	CPH	7,4	12	1,45	this study
Bear 06 ABBconcr -155	Aal	*concavum*	*limitatum*	4280	64,976	belemnite		0,48	0,61	CPH	8,4	18	1,40	this study
Bear 07 ABBconcr -149	Aal	*concavum*	*limitatum*	4286	64,980	belemnite		-0,38	0,68	CPH	9,2	88	1,38	this study
Bear 07 ABBconcr -149 A	Aal	*concavum*	*limitatum*	4286	64,980	belemnite		0,71	0,77	CPH	7,0	16	1,39	this study
Bear 07 ABBconcr -149 C	Aal	*concavum*	*limitatum*	4286	64,980	belemnite		0,12	0,38	CPH	8,2	12	1,37	this study
Bear 07 ABBconcr -149 B	Aal	*concavum*	*limitatum*	4286	64,980	belemnite		0,99	0,40	CPH	8,5	11	1,44	this study

Bear 13 ABBconcr -145	Aal	*concavum*	*limitatum*	4290	64,984	belemnite		1,19	0,94	CPH	6,2	5	1,41	this study
Bear 13 ABBconcr -145	Aal	*concavum*	*limitatum*	4290	64,984	belemnite		1,02	0,92	CPH	6,6	2	1,53	this study
base Bajocian	Baj	*discites*	-	4310	65,000									this study
base *discites* z	Baj	*discites*	-	4310	65,000									this study
Bear 04 ABBconcr -22	Baj	*discites*	-	4413	65,038	belemnite		0,11	0,27	CPH	10,8	4	1,68	this study
Bear 04 ABBconcr -22	Baj	*discites*	-	4413	65,038	belemnite		0,07	0,13	CPH	10,6	4	1,66	this study
Bear 01 ABBconcr -6	Baj	*discites*	-	4429	65,044	belemnite		-0,88	0,74	CPH	11,0	6	1,42	this study
Bear 01 ABBconcr -6	Baj	*discites*	-	4429	65,044	belemnite		-0,62	0,61	CPH	11,1	10	1,48	this study
Bear 02 ABBconcr +7	Baj	*discites*	-	4442	65,049	belemnite		0,90	0,59	CPH	7,2	10	1,44	this study
Bear 02 ABBconcr +7 A	Baj	*discites*	-	4442	65,049	belemnite		0,96	0,29	CPH	8,6	5	1,51	this study
Bear 02 ABBconcr +7 B	Baj	*discites*	-	4442	65,049	belemnite		1,11	0,56	CPH	8,3	22	1,49	this study
B13	Baj	*discites*	-	4510	65,074	belemnite		1,42	-0,26					Jenkyns et al. 2002[5]
Bea 7	Baj	*discites*	-	5085	65,288	belemnite		2,51	0,77	CPH		20	1,61	this study (element data Ullmann et al. 2013[4])
Bea 7	Baj	*discites*	-	5085	65,288	belemnite		2,00	0,79	CPH		55	1,92	this study (element data Ullmann et al. 2013[4])
Bea 6	Baj	*discites*	-	5125	65,303	belemnite		1,53	1,02	CPH		10	1,78	this study (element data Ullmann et al. 2013[4])
Bea 6	Baj	*discites*	-	5125	65,303	belemnite		1,59	0,93	CPH		28	2,11	this study (element data Ullmann et al. 2013[4])
Bea 1	Baj	*discites*	-	5140	65,309	belemnite		0,32	0,92	CPH		12	1,48	this study (element data Ullmann et al. 2013[4])
Bea 1	Baj	*discites*	-	5140	65,309	belemnite		0,97	0,16	CPH		101	1,95	this study (element data Ullmann et al. 2013[4])
Bea 2	Baj	*discites*	-	5160	65,316	belemnite		1,01	0,99	CPH		14	1,50	this study (element data Ullmann et al. 2013[4])
Bea 2	Baj	*discites*	-	5160	65,316	belemnite		1,03	1,09	CPH		13	1,57	this study (element data Ullmann et al. 2013[4])
Bea 3	Baj	*discites*	-	5245	65,348	belemnite		1,32	1,31	CPH		10	1,51	this study (element data Ullmann et al. 2013[4])
Bea 3	Baj	*discites*	-	5245	65,348	belemnite		1,44	1,62	CPH		13	1,49	this study (element data Ullmann et al. 2013[4])
B14a	Baj	*discites*	-	5250	65,349	belemnite		1,79	0,34					Jenkyns et al. 2002[5]
B14b	Baj	*discites*	-	5250	65,349	belemnite		1,37	0,60					Jenkyns et al. 2002[5]
Bea 4	Baj	*discites*	-	5250	65,349	belemnite		-0,06	0,99	CPH		11	1,37	this study (element data Ullmann et al. 2013[4])
Bea 4	Baj	*discites*	-	5250	65,349	belemnite		1,14	1,35	CPH		7	1,38	this study (element data Ullmann et al. 2013[4])
Bea 10	Baj	*discites*	-	5275	65,359	belemnite		1,69	-0,22	CPH		11	1,88	this study (element data Ullmann et al. 2013[4])
Bea 10	Baj	*discites*	-	5275	65,359	belemnite		0,99	-0,02	CPH		17	1,90	this study (element data Ullmann et al. 2013[4])
Bea 12	Baj	*discites*	-	5330	65,379	belemnite		-1,16	0,99	CPH		10	1,46	this study (element data Ullmann et al. 2013[4])
Bea 12	Baj	*discites*	-	5330	65,379	belemnite		-0,26	0,62	CPH		7	1,47	this study (element data Ullmann et al. 2013[4])
Bea 13	Baj	*discites*	-	5500	65,442	belemnite		-0,09	1,04	CPH		13	1,43	this study (element data Ullmann et al. 2013[4])
Bea 13	Baj	*discites*	-	5500	65,442	belemnite		-0,02	0,74	CPH		9	1,65	this study (element data Ullmann et al. 2013[4])
B15	Baj	*discites*	-	5540	65,457	belemnite		0,63	0,49					Jenkyns et al. 2002[5]
Bea 15	Baj	*discites*	-	5680	65,509	belemnite		1,15	0,90	CPH		5	1,47	this study (element data Ullmann et al. 2013[4])
Bea 15	Baj	*discites*	-	5680	65,509	belemnite		1,64	0,93	CPH		11	1,51	this study (element data Ullmann et al. 2013[4])
Bear 502	Baj	*discites*	-	5920	65,599	belemnite	altered	0,09	-0,76	CPH	7,7	26	1,28	this study
Bear 505	Baj	*discites*	-	6200	65,703	belemnite		-0,41	0,87	CPH	11,1	51	1,47	this study
Bear 508	Baj	no information	no information	6380	65,770	belemnite		0,88	1,04	CPH	6,5	2	1,33	this study
Bear 512	Baj	no information	no information	6660	65,874	belemnite		1,60	0,86	CPH	7,5	41	1,35	this study
B19	Baj	no information	no information	6716	65,894	belemnite		-0,14	1,06					Jenkyns et al. 2002[5]
B20	Baj	no information	no information	6755	65,909	belemnite		1,23	1,38					Jenkyns et al. 2002[5]
Bear 509	Baj	no information	no information	6765	65,913	belemnite		0,77	1,29	CPH	8,0	1	1,28	this study
Bear 510	Baj	no information	no information	6790	65,922	belemnite		0,57	1,15	CPH	7,7	2	1,37	this study
B21	Baj	no information	no information	6795	65,924	belemnite		1,47	1,14					Jenkyns et al. 2002[5]
Bear 511	Baj	no information	no information	6850	65,944	belemnite		0,91	1,43	CPH	7,9	1	1,34	this study
Bea 17	Baj	no information	no information	6910	65,967	belemnite		0,29	1,48	CPH		8	1,33	this study (element data Ullmann et al. 2013[4])
Bea 17	Baj	no information	no information	6910	65,967	belemnite		0,05	1,26	CPH		7	1,26	this study (element data Ullmann et al. 2013[4])
Bea 18	Baj	no information	no information	6910	65,967	belemnite		0,61	0,84	CPH		4	1,37	this study (element data Ullmann et al. 2013[4])
Bea 18	Baj	no information	no information	6910	65,967	belemnite		1,42	0,90	CPH		4	1,48	this study (element data Ullmann et al. 2013[4])
Bea 19	Baj	no information	no information	6930	65,974	belemnite		2,30	1,81	CPH		8	1,33	this study (element data Ullmann et al. 2013[4])
Bea 19	Baj	no information	no information	6930	65,974	belemnite		1,38	1,28	CPH		10	1,41	this study (element data Ullmann et al. 2013[4])
base *ovalis* z	Baj	no information	no information	7000	66,000									this study
Bear 513	Baj	no information	no information	7065	66,081	belemnite		1,39	1,57	CPH	5,9	5	1,37	this study
Bea 23	Baj	no information	no information	7185	66,231	belemnite		1,08	1,25	CPH		8	1,35	this study (element data Ullmann et al. 2013[4])
Bea 23	Baj	no information	no information	7185	66,231	belemnite		0,98	0,93	CPH		12	1,33	this study (element data Ullmann et al. 2013[4])
Bea 21	Baj	no information	no information	7195	66,244	belemnite		1,46	1,17	CPH		8	1,45	this study (element data Ullmann et al. 2013[4])
Bea 21	Baj	no information	no information	7195	66,244	belemnite		0,82	0,95	CPH		6	1,47	this study (element data Ullmann et al. 2013[4])
Bea 22	Baj	no information	no information	7195	66,244	belemnite		0,80	1,20	CPH		5	1,50	this study (element data Ullmann et al. 2013[4])
Bea 22	Baj	no information	no information	7195	66,244	belemnite		2,47	1,33	CPH		7	1,71	this study (element data Ullmann et al. 2013[4])
Bea 30	Baj	no information	no information	7355	66,444	belemnite		1,22	1,08	CPH		9	1,39	this study (element data Ullmann et al. 2013[4])
Bea 30	Baj	no information	no information	7355	66,444	belemnite		2,37	1,40	CPH		7	1,56	this study (element data Ullmann et al. 2013[4])
Bea 28	Baj	no information	no information	7375	66,469	belemnite		0,03	1,26	CPH		5	1,39	this study (element data Ullmann et al. 2013[4])
Bea 28	Baj	no information	no information	7375	66,469	belemnite		0,68	0,62	CPH		8	1,53	this study (element data Ullmann et al. 2013[4])
Bea 32	Baj	no information	no information	7490	66,613	belemnite		1,02	1,19	CPH		6	1,39	this study (element data Ullmann et al. 2013[4])
Bea 32	Baj	no information	no information	7490	66,613	belemnite		-0,13	0,81	CPH		18	1,56	this study (element data Ullmann et al. 2013[4])
base *laeviuscula* z	Baj			7800	67,000		approximated							this study
base sayni sz	Baj			7800	67,000		approximated							this study
B22	Baj	no information	no information	8300	67,909	belemnite		1,27	0,96					Jenkyns et al. 2002[5]
base *trigonalis* sz	Baj			8350	68,000		approximated							this study
Bea 35	Baj	no information	no information	8370	68,071	belemnite		2,45	0,61	CPH		5	1,64	this study (element data Ullmann et al. 2013[4])
Bea 35	Baj	no information	no information	8370	68,071	belemnite		2,91	0,30	CPH		5	1,68	this study (element data Ullmann et al. 2013[4])
Bea 40	Baj	no information	no information	8540	68,679	oyster		1,72	0,13	CPH		199	0,72	this study
Bea 40	Baj	no information	no information	8540	68,679	oyster		3,06	0,82	CPH		34	0,66	this study
Bea 40	Baj	no information	no information	8540	68,679	oyster		3,09	0,84	CPH		19	0,60	this study
Bea 40	Baj	no information	no information	8540	68,679	oyster		2,90	0,91	CPH		27	0,61	this study
Bea 38	Baj	no information	no information	8540	68,679	belemnite		-0,70	0,62	CPH		21	1,73	this study (element data Ullmann et al. 2013[4])
Bea 38	Baj	no information	no information	8540	68,679	belemnite		-1,98	0,08	CPH		33	1,97	this study (element data Ullmann et al. 2013[4])
Bear 526	Baj	no information	no information	8540	68,679	belemnite		3,50	-0,71	CPH	11,6	0	1,91	this study
Bea 36	Baj	no information	no information	8580	68,821	belemnite		2,89	0,47	CPH		10	1,77	this study (element data Ullmann et al. 2013[4])
Bea 36	Baj	no information	no information	8580	68,821	belemnite		2,49	0,13	CPH		23	1,70	this study (element data Ullmann et al. 2013[4])

base *laeviuscula* sz	Baj			8630	69,000		approximated							this study
Bear 524	Baj	*laeviuscula*	*laeviuscula*	8690	69,022	oyst		3,97	1,29	CPH	1,8	23	0,66	this study
Bear 525	Baj	*laeviuscula*	*laeviuscula*	8730	69,037	bel		1,67	1,25	CPH	10,6	1	1,30	this study
Bea 44	Baj	*laeviuscula*	*laeviuscula*	8760	69,048	belemnite		2,56	0,84	CPH		9	1,55	this study (element data Ullmann et al. 2013[4])
Bea 44	Baj	*laeviuscula*	*laeviuscula*	8760	69,048	belemnite		2,84	0,77	CPH		6	1,74	this study (element data Ullmann et al. 2013[4])
Bea 46	Baj	*laeviuscula*	*laeviuscula*	8940	69,114	oyster		3,98	1,20	CPH		10	0,66	this study
Bea 46	Baj	*laeviuscula*	*laeviuscula*	8940	69,114	oyster		3,89	1,21	CPH		11	0,65	this study
Bea 47	Baj	*laeviuscula*	*laeviuscula*	8940	69,114	oyster		3,79	1,37	CPH		25	0,65	this study
Bea 47	Baj	*laeviuscula*	*laeviuscula*	8940	69,114	oyster		3,72	1,43	CPH		18	0,67	this study
Bea 47	Baj	*laeviuscula*	*laeviuscula*	8940	69,114	oyster		3,65	1,17	CPH		19	0,60	this study
Bea 47	Baj	*laeviuscula*	*laeviuscula*	8940	69,114	oyster		3,72	1,12	CPH		10	0,58	this study
Bea 50	Baj	*laeviuscula*	*laeviuscula*	9010	69,140	belemnite		0,29	1,01	CPH		7	1,31	this study (element data Ullmann et al. 2013[4])
Bea 50	Baj	*laeviuscula*	*laeviuscula*	9010	69,140	belemnite		0,91	0,71	CPH		7	1,40	this study (element data Ullmann et al. 2013[4])
Bea 54	Baj	*laeviuscula*	*laeviuscula*	9210	69,214	belemnite		2,95	0,42	CPH		8	1,71	this study (element data Ullmann et al. 2013[4])
Bea 54	Baj	*laeviuscula*	*laeviuscula*	9210	69,214	belemnite		2,94	0,43	CPH		11	1,75	this study (element data Ullmann et al. 2013[4])
Bear 103	Baj	*laeviuscula*	*laeviuscula*	9280	69,240	belemnite		3,89	1,04	CPH	9,2	5	1,82	this study
Bear 102	Baj	*laeviuscula*	*laeviuscula*	9305	69,249	belemnite		2,46	1,11	CPH	17,4	12	1,68	this study
Bear 101	Baj	*laeviuscula*	*laeviuscula*	9310	69,251	belemnite		3,27	-0,02	CPH	9,1	24	1,71	this study
Bear 100	Baj	*laeviuscula*	*laeviuscula*	9315	69,253	belemnite		2,97	0,30	CPH	7,9	5	1,46	this study
Bear 107	Baj	*laeviuscula*	*laeviuscula*	9515	69,327	belemnite		2,38	-0,04	CPH	8,7	8	1,55	this study
Bear 106	Baj	*laeviuscula*	*laeviuscula*	9520	69,328	belemnite		3,25	0,49	CPH	10,2	8	1,65	this study
Bear 104	Baj	*laeviuscula*	*laeviuscula*	9550	69,339	belemnite		3,16	0,58	CPH	7,8	18	1,60	this study
B23	Baj	*laeviuscula*	*laeviuscula*	9845	69,448	belemnite		3,67	0,18					Jenkyns et al. 2002[5]
Bear 110	Baj	*laeviuscula*	*laeviuscula*	10190	69,576	belemnite		2,77	0,14	CPH	9,9	10	1,69	this study
Bear 109	Baj	*laeviuscula*	*laeviuscula*	10195	69,577	belemnite		1,81	0,18	CPH	9,5	8	1,42	this study
Bear 109	Baj	*laeviuscula*	*laeviuscula*	10195	69,577	belemnite		2,81	0,29	CPH	9,0	5	1,59	this study
Bear 111	Baj	*laeviuscula*	*laeviuscula*	10195	69,577	belemnite		2,32	-0,07	CPH	9,2	10	1,48	this study
Bear 112	Baj	*laeviuscula*	*laeviuscula*	10450	69,672	belemnite	altered	0,28	-16,30	CPH	19,6	901	1,50	this study
Bear 114	Baj	*laeviuscula*	*laeviuscula*	10460	69,675	belemnite		2,70	0,03	CPH	8,0	4	1,53	this study
Bear 113	Baj	*laeviuscula*	*laeviuscula*	10465	69,677	belemnite		2,89	0,13	CPH	6,9	5	1,41	this study
Bear 115	Baj	*laeviuscula*	*laeviuscula*	10540	69,705	belemnite	altered	-0,09	-11,73	CPH	15,2	500	1,04	this study
Bear 115	Baj	*laeviuscula*	*laeviuscula*	10540	69,705	belemnite	altered	-3,88	-9,67	CPH	9,7	471	0,85	this study
Bear 116	Baj	*laeviuscula*	*laeviuscula*	10545	69,707	belemnite		2,37	0,37	CPH	10,0	-1	1,56	this study
Bear 116	Baj	*laeviuscula*	*laeviuscula*	10545	69,707	belemnite		1,98	0,57	CPH	9,5	1	1,52	this study
B24	Baj	*laeviuscula*	*laeviuscula*	11030	69,886	belemnite		3,09	1,40					Jenkyns et al. 2002[5]
Bear 118	Baj	*laeviuscula*	*laeviuscula*	11245	69,965	belemnite	altered	2,21	-1,81	CPH	12,0	65	1,85	this study
Bear 119	Baj	*laeviuscula*	*laeviuscula*	11300	69,985	belemnite	altered	0,92	-4,50	CPH	11,7	210	1,36	this study
base *sauzei* z	Baj			11340	70,000		approximated							this study
Bear 123	Baj	*sauzei*	-	12000	70,892	belemnite	altered	1,82	-13,64	CPH	5,5	846	0,39	this study
base *humphriesianum* z	Baj			12080	71,000		approximated							this study
base *romani* sz	Baj			12080	71,000		approximated							this study
Bear 120	Baj	*humphriesianum*	*romani*	12080	71,000	belemnite	altered	2,87	-1,19	CPH	9,2	33	1,68	this study
Bear 121	Baj	*humphriesianum*	*romani*	12080	71,000	belemnite	altered	0,02	-9,17	CPH	7,3	42	1,07	this study
Bear 128	Baj	*humphriesianum*	*romani*	12540	71,277	belemnite	altered	1,11	-2,75	CPH	21,8	31	1,10	this study
Bear 131	Baj	*humphriesianum*	*romani*	12550	71,283	belemnite	altered	1,67	-1,55	CPH	9,0	4	1,75	this study
Bear 142 A	Baj	*humphriesianum*	*romani*	12650	71,343	belemnite	altered	-0,03	-0,14	CPH	8,5	143	1,39	this study
Bear 142 B	Baj	*humphriesianum*	*romani*	12650	71,343	belemnite		2,73	-0,73	CPH	9,3	9	1,75	this study
Bear 134	Baj	*humphriesianum*	*romani*	12660	71,349	belemnite	altered	2,27	-2,65	CPH	15,3	82	1,94	this study
Bear 143	Baj	*humphriesianum*	*romani*	12660	71,349	belemnite		0,80	-0,59	CPH	11,8	10	1,60	this study
Bear 144	Baj	*humphriesianum*	*romani*	12690	71,367	belemnite		0,79	-0,07	CPH	8,5	46	1,68	this study
Bear 141	Baj	*humphriesianum*	*romani*	12695	71,370	belemnite	altered	2,03	0,11	CPH	19,2	108	2,11	this study
Bear 145	Baj	*humphriesianum*	*romani*	12760	71,410	belemnite		0,99	0,09	CPH	8,3	2	1,66	this study
Bear 146	Baj	*humphriesianum*	*romani*	12760	71,410	belemnite		1,43	-1,22	CPH	7,7	-1	1,74	this study
Bear 148	Baj	*humphriesianum*	*romani*	12775	71,419	oyster		0,37	1,39	CPH				this study
Bear 149	Baj	*humphriesianum*	*romani*	12780	71,422	belemnite	altered	-0,65	0,49	CPH	7,5	161	1,21	this study
Bear 150	Baj	*humphriesianum*	*romani*	12830	71,452	belemnite	altered	0,93	-0,66	CPH	6,9	1	1,49	this study
Bear 151	Baj	*humphriesianum*	*romani*	12830	71,452	belemnite	altered	3,20	-0,06	CPH	11,4	19	2,23	this study
Bear 152	Baj	*humphriesianum*	*romani*	12860	71,470	belemnite		1,11	-0,86	CPH	6,7	9	1,68	this study
Bear 153	Baj	*humphriesianum*	*romani*	12860	71,470	belemnite	altered	1,58	-3,15	CPH	8,8	45	1,52	this study
Bear 155	Baj	*humphriesianum*	*romani*	12860	71,470	belemnite	altered	2,21	-2,19	CPH	9,9	18	1,47	this study
Bear 160	Baj	*humphriesianum*	*romani*	13260	71,711	belemnite		2,18	0,83	CPH	12,2	17	1,38	this study
Bear 164	Baj	*humphriesianum*	*romani*	13365	71,774	belemnite	altered	1,27	-0,18	CPH	6,8	11	1,45	this study
Bear 164	Baj	*humphriesianum*	*romani*	13365	71,774	belemnite	altered	0,13	-0,74	CPH	7,1	18	1,12	this study
Bear 162	Baj	*humphriesianum*	*romani*	13445	71,822	belemnite	altered	2,52	-10,47	CPH	6,9	33	1,15	this study
Bear 163	Baj	*humphriesianum*	*romani*	13445	71,822	belemnite		1,77	0,39	CPH	8,1	1	1,50	this study
Bear 165	Baj	*humphriesianum*	*romani*	13540	71,880	belemnite		0,92	0,33	CPH	15,9	10	1,62	this study
Bear 179	Baj	*humphriesianum*	*romani*	13540	71,880	belemnite		2,07	0,15	CPH	8,5	-2	1,69	this study
Bear 185	Baj	*humphriesianum*	*romani*	13540	71,880	belemnite	altered	-3,95	-4,50	CPH	5,2	115	0,78	this study
Bear 166	Baj	*humphriesianum*	*romani*	13570	71,898	belemnite		0,50	-0,71	CPH	11,4	44	1,60	this study
Bear 168	Baj	*humphriesianum*	*romani*	13640	71,940	belemnite	altered	2,39	-0,51	CPH	14,4	37	1,92	this study
Bear 172	Baj	*humphriesianum*	*romani*	13655	71,949	belemnite		1,30	1,00	CPH	12,5	8	1,42	this study
Bear 170	Baj	*humphriesianum*	*romani*	13720	71,988	belemnite	altered	2,51	-1,77	CPH	9,0	20	1,81	this study
base *humphriesianum* sz	Baj			13740	72,000		approximated							this study
Bear 180	Baj	*humphriesianum*	*humphriesianum*	13740	72,000	belemnite		3,43	0,24	CPH	10,6	-2	1,81	this study
Bear 171	Baj	*humphriesianum*	*humphriesianum*	13780	72,032	belemnite	altered	1,06	-6,06	CPH	6,9	5	1,44	this study
Bear 178	Baj	*humphriesianum*	*humphriesianum*	13815	72,060	belemnite		1,64	-0,46	CPH	10,2	36	1,74	this study
Bear 173	Baj	*humphriesianum*	*humphriesianum*	13830	72,071	belemnite	altered	2,17	0,45	CPH	7,8	5	1,82	this study
Bear 174	Baj	*humphriesianum*	*humphriesianum*	13850	72,087	belemnite	altered	1,98	-1,38	CPH	9,7	77	1,58	this study
Bear 177	Baj	*humphriesianum*	*humphriesianum*	13860	72,095	belemnite		-0,81	0,17	CPH	6,5	113	0,91	this study

Bear 181	Baj	*humphriesianum*	*humphriesianum*	13900	72,127	belemnite	altered	1,78	-9,66	CPH	7,8	99	1,18	this study
Bear 182	Baj	*humphriesianum*	*humphriesianum*	13900	72,127	belemnite	altered	1,34	-5,34	CPH	6,3	13	1,38	this study
Bear 184	Baj	*humphriesianum*	*humphriesianum*	14050	72,246	belemnite		0,92	-1,23	CPH	8,8	11	1,71	this study
Bear 186	Baj	*humphriesianum*	*humphriesianum*	14080	72,270	belemnite		-0,45	0,21	CPH	21,0	16	1,50	this study
Bear 190	Baj	*humphriesianum*	*humphriesianum*	14690	72,754	belemnite		2,73	0,18	CPH	5,7	27	1,31	this study
Bear 188	Baj	*humphriesianum*	*humphriesianum*	14760	72,810	belemnite	altered	0,27	-0,21	CPH	8,8	3	1,70	this study
Bear 189	Baj	*humphriesianum*	*humphriesianum*	14830	72,865	belemnite	altered	2,26	-1,74	CPH	9,1	39	1,72	this study
Bear 192	Baj	*humphriesianum*	*humphriesianum*	14840	72,873	belemnite	altered	0,90	-1,12	CPH	14,2	8	1,43	this study
base *blagdeni* sz	Baj			15000	73,000		approximated							this study

literature data

Inverarish Burn and Beinn na Leac (Raasay)

RY11a	Toa	*falciferum*	0,05	45,000	belemnite	3,01	-5,12	Price 2010[6]
RY11b	Toa	*falciferum*	0,05	45,000	belemnite	3,31	-5,22	Price 2010[6]
RY11c	Toa	*falciferum*	0,05	45,000	belemnite	2,54	-5,19	Price 2010[6]
RY11d	Toa	*falciferum*	0,05	45,000	belemnite	3,61	-4,54	Price 2010[6]
RY11e	Toa	*falciferum*	0,05	45,000	belemnite	3,43	-5,13	Price 2010[6]
RY1	Toa	*bifrons*	1,7	48,571	belemnite	0,52	-4,13	Price 2010[6]
RY3	Toa	*aalensis*	7,54	55,638	belemnite	2,46	-1,65	Price 2010[6]
RY4b	Toa	*aalensis*	10,14	55,954	belemnite	1,06	-1,73	Price 2010[6]
RY5	Toa	*aalensis*	9,84	55,904	belemnite	1,04	-1,36	Price 2010[6]
RY6x	Toa	*aalensis*	9,14	55,827	belemnite	2,66	-1,41	Price 2010[6]
RY2	Aal	*opalinum*	11	56,100	belemnite	2,24	-1,45	Price 2010[6]
RY68	Aal	*opalinum*	6,7	56,240	belemnite	1,13	-0,79	Price 2010[6]
RY63	Aal	*opalinum*	6,9	56,272	belemnite	1,15	-1,51	Price 2010[6]
RY64a	Aal	*opalinum*	6,9	56,272	belemnite	1,31	-1,42	Price 2010[6]
RY64b	Aal	*opalinum*	7,1	56,304	belemnite	-0,07	-0,70	Price 2010[6]
RY67**	Aal	*opalinum*	7,1	56,304	belemnite	1,82	-6,01	Price 2010[6]
RY61**	Aal	*opalinum*	7,7	56,384	belemnite	-0,38	-6,39	Price 2010[6]
RY65**	Aal	*opalinum*	7,7	56,384	belemnite	0,05	-4,19	Price 2010[6]
RY60a	Aal	*opalinum*	7,7	56,384	belemnite	2,12	-2,35	Price 2010[6]
RY60b	Aal	*opalinum*	7,7	56,384	belemnite	1,09	-1,38	Price 2010[6]
RY66	Aal	*opalinum*	7,7	56,384	belemnite	1,52	-1,52	Price 2010[6]
RY62	Aal	*opalinum*	7,8	56,400	belemnite	-0,08	-0,82	Price 2010[6]
RY13	Aal	*opalinum*	9	56,560	belemnite	1,32	-1,28	Price 2010[6]
RY72	Aal	*opalinum*	9	56,560	belemnite	0,94	-2,32	Price 2010[6]
RY14	Aal	*opalinum*	9,6	56,640	belemnite	0,60	-1,82	Price 2010[6]
RY15	Aal	*opalinum*	10	56,704	belemnite	1,10	-1,26	Price 2010[6]
RY69**	Aal	*opalinum*	10	56,704	belemnite	1,04	-4,68	Price 2010[6]
RY70	Aal	*opalinum*	11,3	56,880	belemnite	1,88	-0,21	Price 2010[6]
RY16	Aal	*opalinum*	11,4	56,896	belemnite	2,05	1,13	Price 2010[6]
RY17	Aal	*opalinum*	11,6	56,936	belemnite	2,84	-0,01	Price 2010[6]
RY18	Aal	*opalinum*	11,6	56,936	belemnite	2,63	0,18	Price 2010[6]
RY20	Aal	*opalinum*	11,7	56,944	belemnite	2,64	-0,43	Price 2010[6]
RY22	Aal	*opalinum*	11,7	56,944	belemnite	2,05	-0,20	Price 2010[6]
RY21	Aal	*opalinum*	11,8	56,952	belemnite	3,16	0,82	Price 2010[6]
RY24	Aal	*opalinum*	11,9	56,960	belemnite	2,96	0,35	Price 2010[6]
RY23	Aal	*opalinum*	12	56,968	belemnite	3,29	-0,07	Price 2010[6]
RY26a	Aal	*opalinum*	12,1	56,984	belemnite	2,52	1,24	Price 2010[6]
RY26b	Aal	*opalinum*	12,1	56,984	belemnite	2,80	1,25	Price 2010[6]
RY26c	Aal	*opalinum*	12,1	56,984	belemnite	1,34	0,75	Price 2010[6]
RY58	Aal	*opalinum*	12,2	56,992	belemnite	2,97	0,04	Price 2010[6]
RY29	Aal	*scissum*	12,4	57,128	belemnite	2,95	0,39	Price 2010[6]
RY30	Aal	*scissum*	12,4	57,128	belemnite	2,38	-0,23	Price 2010[6]
RY25	Aal	*scissum*	12,8	57,255	belemnite	3,26	-0,26	Price 2010[6]
RY31	Aal	*scissum*	12,95	57,277	belemnite	2,41	-0,79	Price 2010[6]
RY27a	Aal	*scissum*	13	57,298	belemnite	3,71	0,23	Price 2010[6]
RY27b	Aal	*scissum*	13	57,298	belemnite	4,08	-0,10	Price 2010[6]
RY28	Aal	*scissum*	13,1	57,351	belemnite	4,52	0,21	Price 2010[6]
RY33	Aal	*scissum*	14	57,638	belemnite	3,34	-0,16	Price 2010[6]
RY34	Aal	*scissum*	14,4	57,787	belemnite	1,14	-0,55	Price 2010[6]
RY35	Aal	*scissum*	14,4	57,787	belemnite	1,91	0,67	Price 2010[6]
RY36	Aal	*murchisonae*	14,8	58,146	belemnite	1,83	0,36	Price 2010[6]
RY59	Aal	*murchisonae*	14,8	58,146	belemnite	2,88	-0,02	Price 2010[6]
RY32	Aal	*murchisonae*	15,2	58,585	belemnite	2,71	-0,26	Price 2010[6]
RY54	Aal	*murchisonae*	15,6	58,988	belemnite	1,85	0,88	Price 2010[6]
RY55	Aal	*murchisonae*	15,9	59,463	belemnite	3,19	0,18	Price 2010[6]
RY56**	Aal	*murchisonae*	16,5	60,049	belemnite	2,61	0,64	Price 2010[6]
RY37	Aal	*murchisonae*	16,7	60,415	belemnite	2,15	0,56	Price 2010[6]
RY57a	Aal	*murchisonae*	16,7	60,415	belemnite	2,05	0,71	Price 2010[6]
RY57B	Aal	*murchisonae*	16,7	60,415	belemnite	3,29	0,64	Price 2010[6]
RY38a	Aal	*concavum*	17,4	63,276	belemnite	1,69	0,79	Price 2010[6]
RY38b	Aal	*concavum*	17,4	63,276	belemnite	1,31	0,45	Price 2010[6]
RY38c	Aal	*concavum*	17,4	63,276	belemnite	1,29	0,58	Price 2010[6]
RY75	Aal	*concavum*	18	63,966	belemnite	1,94	0,44	Price 2010[6]
RY73	Aal	*concavum*	18,1	64,172	belemnite	2,82	0,21	Price 2010[6]

RY71**	Aal	*concavum*		18,2	64,241	belemnite		2,09	-0,95				Price 2010[6]
RY47**	Aal	*concavum*		18,4	64,448	belemnite		1,10	-3,38				Price 2010[6]
RY74	Aal	*concavum*		18,6	64,655	belemnite		2,67	0,45				Price 2010[6]
RY46	Baj	*discites*		19	65,036	belemnite		3,39	-0,65				Price 2010[6]
RY48	Baj	*discites*		19	65,036	belemnite		3,45	0,52				Price 2010[6]
RY41	Baj	*discites*		19,1	65,045	belemnite		2,78	-0,79				Price 2010[6]
RY42	Baj	*discites*		19,1	65,045	belemnite		3,65	-0,37				Price 2010[6]
RY43	Baj	*discites*		19,2	65,073	belemnite		3,56	0,03				Price 2010[6]
RY39	Baj	*discites*		19,45	65,109	belemnite		1,68	-0,15				Price 2010[6]
RY40	Baj	*discites*		19,45	65,109	belemnite		2,03	0,21				Price 2010[6]
RY44	Baj	*discites*		19,65	65,145	belemnite		1,49	1,36				Price 2010[6]
RY53	Baj	*discites*		19,65	65,145	belemnite		1,00	-0,20				Price 2010[6]
RY49	Baj	*discites*		19,7	65,155	belemnite		1,76	0,30				Price 2010[6]
RY45	Baj	*discites*		19,9	65,182	belemnite		3,07	-0,15				Price 2010[6]
RY52	Baj	*discites*		20,1	65,205	belemnite		2,84	0,49				Price 2010[6]
RY50	Baj	*discites*		21,2	65,427	belemnite		3,68	1,00				Price 2010[6]
RY51	Baj	*discites*		21,5	65,427	belemnite		2,31	-0,49				Price 2010[6]

Yorkshire

<u>our data (including new and literature data)</u>

Robin Hood's Bay - Hawsker High Scar

base *davoei* z	Pli			7675	32,000								
base *maculatum* sz	Pli			7675	32,000								
Hood 297 A	Pli	*davoei*	*maculatum*	7680	32,003	pectinid		2,14	-2,52	B	468	0,66	Korte & Hesselbo 2011[2]
Hood 297 A	Pli	*davoei*	*maculatum*	7680	32,003	pectinid		3,08	-1,44	B	852	0,77	Korte & Hesselbo 2011[2]
Hood 298	Pli	*davoei*	*maculatum*	7680	32,003	belemnite		1,52	-1,01	B	194	1,41	Korte & Hesselbo 2011[2]
Hood 299	Pli	*davoei*	*maculatum*	7680	32,003	belemnite		1,00	-0,86	B	80	1,34	Korte & Hesselbo 2011[2]
Hood 299	Pli	*davoei*	*maculatum*	7680	32,003	pectinid		2,70	-1,22	B	775	0,66	Korte & Hesselbo 2011[2]
Hood 310 A	Pli	*davoei*	*maculatum*	7690	32,008	pectinid		3,92	-0,98	B	526	0,71	Korte & Hesselbo 2011[2]
Hood 310 A	Pli	*davoei*	*maculatum*	7690	32,008	pectinid		3,54	-0,90	B	579	0,66	Korte & Hesselbo 2011[2]
Hood 291	Pli	*davoei*	*maculatum*	8310	32,347	belemnite	altered	-0,68	-3,29	B	199	1,03	Korte & Hesselbo 2011[2]
Hood 287	Pli	*davoei*	*maculatum*	8565	32,486	pectinid		1,73	-1,98	B	237	0,73	Korte & Hesselbo 2011[2]
Hood 287	Pli	*davoei*	*maculatum*	8565	32,486	pectinid		1,78	-2,67	B	813	0,67	Korte & Hesselbo 2011[2]
Hood 288	Pli	*davoei*	*maculatum*	8565	32,486	pectinid		2,49	-2,58	B	668	0,91	Korte & Hesselbo 2011[2]
Hood 288	Pli	*davoei*	*maculatum*	8565	32,486	pectinid		2,35	-1,91	B	706	0,75	Korte & Hesselbo 2011[2]
Hood 286	Pli	*davoei*	*maculatum*	8670	32,544	belemnite		3,26	-1,40	B	27	1,34	Korte & Hesselbo 2011[2]
Hood 285	Pli	*davoei*	*maculatum*	8850	32,642	belemnite		0,67	-1,87	B	199	1,04	Korte & Hesselbo 2011[2]
Hood 284	Pli	*davoei*	*maculatum*	9075	32,765	belemnite		1,56	-2,31	B	36	1,52	Korte & Hesselbo 2011[2]
Hood 283	Pli	*davoei*	*maculatum*	9128	32,794	belemnite		2,54	-2,17	B	425	2,03	Korte & Hesselbo 2011[2]
Hood 280	Pli	*davoei*	*maculatum*	9350	32,915	brachiopod		1,09	-1,98	B	160	0,78	Korte & Hesselbo 2011[2]
Hood 280	Pli	*davoei*	*maculatum*	9350	32,915	oyster		2,41	-0,64	B	278	0,49	Korte & Hesselbo 2011[2]
Hood 281	Pli	*davoei*	*maculatum*	9438	32,963	belemnite		2,34	-3,46	B	14	1,75	Korte & Hesselbo 2011[2]
Hood 282	Pli	*davoei*	*maculatum*	9438	32,963	oyster		2,12	-0,88	B	187	0,65	Korte & Hesselbo 2011[2]
base *capricornus* sz	Pli			9505	33,000								
Hood 273	Pli	*davoei*	*capricornus*	9515	33,031	oyster		2,78	-2,84	B	214	0,56	Korte & Hesselbo 2011[2]
Hood 273	Pli	*davoei*	*capricornus*	9515	33,031	oyster		3,02	-2,21	B	228	0,53	Korte & Hesselbo 2011[2]
Hood 274 B	Pli	*davoei*	*capricornus*	9560	33,172	oyster		2,01	-1,95	B	234	0,51	Korte & Hesselbo 2011[2]
Hood 276 A	Pli	*davoei*	*capricornus*	9625	33,375	oyster		2,59	-1,61	B	142	0,46	Korte & Hesselbo 2011[2]
Hood 276 B	Pli	*davoei*	*capricornus*	9625	33,375	oyster		3,09	-1,04	B	343	0,60	Korte & Hesselbo 2011[2]
Hood 276 B	Pli	*davoei*	*capricornus*	9625	33,375	oyster		2,60	-1,71	B	676	0,58	Korte & Hesselbo 2011[2]
Hood 277	Pli	*davoei*	*capricornus*	9625	33,375	belemnite		1,99	-3,55	B	22	1,39	Korte & Hesselbo 2011[2]
Hood 275	Pli	*davoei*	*capricornus*	9655	33,469	oyster		2,93	-1,31	B	163	0,48	Korte & Hesselbo 2011[2]
Hood 279	Pli	*davoei*	*capricornus*	9695	33,594	oyster		3,06	-1,35	B	71	0,44	Korte & Hesselbo 2011[2]
base *figulinum* sz	Pli			9825	34,000								
Hood 270	Pli	*davoei*	*figulinum*	9909	34,096	oyster		2,51	-2,61	B	396	0,50	Korte & Hesselbo 2011[2]
Hood 271	Pli	*davoei*	*figulinum*	10015	34,217	oyster		2,44	-1,50	B	208	0,54	Korte & Hesselbo 2011[2]
Hood 331 A	Pli	*davoei*	*figulinum*	10060	34,269	oyster		2,35	-1,79	B	196	0,44	Korte & Hesselbo 2011[2]
Hood 261	Pli	*davoei*	*figulinum*	10290	34,531	oyster		3,39	-1,47	B	80	0,37	Korte & Hesselbo 2011[2]
Hood 264	Pli	*davoei*	*figulinum*	10340	34,589	oyster		3,08	-1,74	B	547	0,50	Korte & Hesselbo 2011[2]
Hood 254	Pli	*davoei*	*figulinum*	10375	34,629	oyster		3,20	-1,87	B	36	0,39	Korte & Hesselbo 2011[2]
Hood 254	Pli	*davoei*	*figulinum*	10375	34,629	oyster		3,70	-1,76	B	44	0,42	Korte & Hesselbo 2011[2]
Hood 255	Pli	*davoei*	*figulinum*	10375	34,629	oyster		1,37	-2,57	B	163	0,55	Korte & Hesselbo 2011[2]
Hood 256	Pli	*davoei*	*figulinum*	10375	34,629	oyster		2,65	-2,31	B	160	0,44	Korte & Hesselbo 2011[2]
Hood 256	Pli	*davoei*	*figulinum*	10375	34,629	oyster		2,51	-1,70	B	168	0,33	Korte & Hesselbo 2011[2]
Hood 259	Pli	*davoei*	*figulinum*	10395	34,651	belemnite		1,86	-2,15	B	46	1,78	Korte & Hesselbo 2011[2]
Hood 260	Pli	*davoei*	*figulinum*	10395	34,651	oyster		2,47	-2,09	B	155	0,51	Korte & Hesselbo 2011[2]
Hood 251	Pli	*davoei*	*figulinum*	10475	34,743	brachiopod		2,02	-2,68	B	371	0,80	Korte & Hesselbo 2011[2]
Hood 251	Pli	*davoei*	*figulinum*	10475	34,743	brachiopod	altered	1,86	-3,18	B	669	0,85	Korte & Hesselbo 2011[2]
Hood 251	Pli	*davoei*	*figulinum*	10475	34,743	oyster		3,24	-2,23	B	238	0,46	Korte & Hesselbo 2011[2]
Hood 300	Pli	*davoei*	*figulinum*	10475	34,743	oyster		3,05	-1,59	B	135	0,53	Korte & Hesselbo 2011[2]
Hood 300	Pli	*davoei*	*figulinum*	10475	34,743	oyster		3,35	-1,31	B	156	0,67	Korte & Hesselbo 2011[2]
Hood 248	Pli	*davoei*	*figulinum*	10515	34,789	oyster		2,49	-2,32	B	302	0,50	Korte & Hesselbo 2011[2]
Hood 307	Pli	*davoei*	*figulinum*	10695	34,994	oyster	altered	2,72	-2,20	B	1664	0,47	Korte & Hesselbo 2011[2]

base *margaritatus* z	Pli				35,000								
base *stokesi* sz	Pli			10700	35,000								
Hood 246	Pli	*margaritatus*	*stokesi*	10705	35,003	oyster		3,06	-1,37	B	172	0,54	Korte & Hesselbo 2011[2]
Hood 250	Pli	*margaritatus*	*stokesi*	10745	35,027	bivalve unident.		2,87	-1,15	B	178	0,61	Korte & Hesselbo 2011[2]
Hood 301	Pli	*margaritatus*	*stokesi*	10755	35,034	oyster		2,73	-1,57	B	97	0,38	Korte & Hesselbo 2011[2]
Hood 302	Pli	*margaritatus*	*stokesi*	10755	35,034	oyster		3,43	-2,21	B	97	0,54	Korte & Hesselbo 2011[2]
Hood 303	Pli	*margaritatus*	*stokesi*	10755	35,034	belemnite		3,01	-2,38	B	30	1,28	Korte & Hesselbo 2011[2]
Hood 304	Pli	*margaritatus*	*stokesi*	10755	35,034	belemnite		1,06	-1,78	B	31	1,35	Korte & Hesselbo 2011[2]
Hood 305	Pli	*margaritatus*	*stokesi*	10755	35,034	oyster		2,67	-1,17	B	244	0,38	Korte & Hesselbo 2011[2]
Hood 305	Pli	*margaritatus*	*stokesi*	10755	35,034	oyster		3,46	-1,47	B	103	0,47	Korte & Hesselbo 2011[2]
Hood 305	Pli	*margaritatus*	*stokesi*	10755	35,034	oyster	altered	1,28	-2,76	B	178	0,61	Korte & Hesselbo 2011[2]
Hood 333	Pli	*margaritatus*	*stokesi*	10962	35,160	oyster		2,58	-1,19	B	181	0,45	Korte & Hesselbo 2011[2]
Hood 334	Pli	*margaritatus*	*stokesi*	11035	35,204	belemnite		2,51	-2,83	B	295	2,04	Korte & Hesselbo 2011[2]
Hood 334	Pli	*margaritatus*	*stokesi*	11035	35,204	brachiopod		2,10	-1,84	B	297	0,84	Korte & Hesselbo 2011[2]
Hood 334	Pli	*margaritatus*	*stokesi*	11035	35,204	oyster		3,16	-1,48	B	101	0,50	Korte & Hesselbo 2011[2]
Hood 338	Pli	*margaritatus*	*stokesi*	11085	35,235	oyster		3,08	-1,24	B	105	0,50	Korte & Hesselbo 2011[2]
Hood 339	Pli	*margaritatus*	*stokesi*	11085	35,235	belemnite		3,47	-2,53	B	109	1,83	Korte & Hesselbo 2011[2]
Hood 342	Pli	*margaritatus*	*stokesi*	11225	35,320	belemnite		2,84	-1,13	B	59	1,62	Korte & Hesselbo 2011[2]
Hood 345 A	Pli	*margaritatus*	*stokesi*	11340	35,390	oyster		1,68	-2,04	B	182	0,41	Korte & Hesselbo 2011[2]
Hood 348	Pli	*margaritatus*	*stokesi*	11820	35,683	belemnite		2,75	-1,23	B	96	1,99	Korte & Hesselbo 2011[2]
Hood 350	Pli	*margaritatus*	*stokesi*	11910	35,738	oyster		1,98	-2,05	B	195	0,50	Korte & Hesselbo 2011[2]
Hood 352	Pli	*margaritatus*	*stokesi*	11920	35,744	oyster		2,82	-1,46	B	139	0,44	Korte & Hesselbo 2011[2]
Hood 353	Pli	*margaritatus*	*stokesi*	11920	35,744	oyster		2,23	-1,37	B	147	0,40	Korte & Hesselbo 2011[2]
Hood 354	Pli	*margaritatus*	*stokesi*	11960	35,768	belemnite		2,14	-2,88	B	60	1,77	Korte & Hesselbo 2011[2]
Hood 356	Pli	*margaritatus*	*stokesi*	12072	35,837	belemnite		2,47	-2,07	B	46	1,50	Korte & Hesselbo 2011[2]
Hood 358	Pli	*margaritatus*	*stokesi*	12072	35,837	oyster		2,02	-2,05	B	125	0,54	Korte & Hesselbo 2011[2]
Hood 363	Pli	*margaritatus*	*stokesi*	12140	35,878	belemnite		3,77	-2,92	B	150	1,67	Korte & Hesselbo 2011[2]
Hood 364	Pli	*margaritatus*	*stokesi*	12150	35,884	belemnite		3,36	-2,55	B	23	1,41	Korte & Hesselbo 2011[2]
Hood 365	Pli	*margaritatus*	*stokesi*	12250	35,945	belemnite		2,37	-0,52	B	17	1,67	Korte & Hesselbo 2011[2]
Hood 369	Pli	*margaritatus*	*stokesi*	12330	35,994	pectinid		3,16	-1,46	B	1473	0,79	Korte & Hesselbo 2011[2]
Hood 370	Pli	*margaritatus*	*stokesi*	12330	35,994	oyster		3,10	-1,00	B	135	0,50	Korte & Hesselbo 2011[2]
Hood 370 A	Pli	*margaritatus*	*stokesi*	12330	35,994	belemnite		1,52	-1,78	B	25	1,60	Korte & Hesselbo 2011[2]
base *subnodosus* sz	Pli			12340	36,000								
Hood 375	Pli	*margaritatus*	*subnodosus*	12410	36,122	pectinid		2,64	-0,91	B	432	0,76	Korte & Hesselbo 2011[2]
Hood 377	Pli	*margaritatus*	*subnodosus*	12410	36,122	belemnite		1,86	-1,55	B	28	1,71	Korte & Hesselbo 2011[2]
Hood 378	Pli	*margaritatus*	*subnodosus*	12472	36,230	belemnite		2,09	-0,80	B	347	1,95	Korte & Hesselbo 2011[2]
Hood 381	Pli	*margaritatus*	*subnodosus*	12830	36,852	belemnite		0,42	-3,98	B	120	1,22	Korte & Hesselbo 2011[2]
Hood 382	Pli	*margaritatus*	*subnodosus*	12900	36,974	oyster		3,38	-0,77	B	211	0,64	Korte & Hesselbo 2011[2]
Hood 383	Pli	*margaritatus*	*subnodosus*	12900	36,974	belemnite		2,87	-0,90	B	73	1,72	Korte & Hesselbo 2011[2]
Hood 384	Pli	*margaritatus*	*subnodosus*	12900	36,974	belemnite		2,17	-1,74	B	46	1,62	Korte & Hesselbo 2011[2]
base *gibbosus* sz	Pli				37,000								
Hood 385	Pli	*margaritatus*	*gibbosus*	12920	37,008	pectinid		3,26	-1,51	B	470	0,75	Korte & Hesselbo 2011[2]
Hood 390	Pli	*margaritatus*	*gibbosus*	13495	37,906	pectinid		3,75	-1,14	B	613	0,63	Korte & Hesselbo 2011[2]
Hood 389	Pli	*margaritatus*	*gibbosus*	13507	37,925	bivalve unident.		4,31	-1,31	B	416	0,71	Korte & Hesselbo 2011[2]
Hood 396	Pli	*margaritatus*	*gibbosus*	13547	37,988	belemnite		2,23	-0,09	B	190	1,88	Korte & Hesselbo 2011[2]
base *spinatum* z	Pli			13555	38,000								
base *apyrenum* sz	Pli			13555	38,000								
Hood 394	Pli	*spinatum*	*apyrenum*	13557	38,003	oyster		2,58	0,01	B	106	0,84	Korte & Hesselbo 2011[2]
Hood 398	Pli	*spinatum*	*apyrenum*	13587	38,054	pectinid		3,47	-1,15	B	183	0,52	Korte & Hesselbo 2011[2]
Hood 399	Pli	*spinatum*	*apyrenum*	13587	38,054	belemnite		2,41	-0,09	B	99	1,99	Korte & Hesselbo 2011[2]
Hood 400 B	Pli	*spinatum*	*apyrenum*	13634	38,133	belemnite		2,48	-0,34	B	15	2,00	Korte & Hesselbo 2011[2]
Hood 400 C	Pli	*spinatum*	*apyrenum*	13634	38,133	oyster		2,10	-1,55	B	112	0,50	Korte & Hesselbo 2011[2]
Hood 400 D	Pli	*spinatum*	*apyrenum*	13634	38,133	pectinid		1,40	-2,42	B	374	0,88	Korte & Hesselbo 2011[2]
Hood 404 A	Pli	*spinatum*	*apyrenum*	13717	38,272	oyster		3,68	-0,41	B	31	0,47	Korte & Hesselbo 2011[2]
Hood 406	Pli	*spinatum*	*apyrenum*	13759	38,343	oyster		3,24	-0,11	B	75	0,44	Korte & Hesselbo 2011[2]
Hood 409	Pli	*spinatum*	*apyrenum*	13851	38,497	pectinid		1,60	-1,38	B	1837	1,08	Korte & Hesselbo 2011[2]
Hood 410	Pli	*spinatum*	*apyrenum*	14028	38,795	belemnite		1,07	-2,25	B	21	1,70	Korte & Hesselbo 2011[2]
Hood 410	Pli	*spinatum*	*apyrenum*	14028	38,795	pectinid		1,97	-2,22	B	257	0,77	Korte & Hesselbo 2011[2]
Hood 414	Pli	*spinatum*	*apyrenum*	14092	38,903	pectinid		1,48	-1,69	B	445	0,84	Korte & Hesselbo 2011[2]
Hood 415	Pli	*spinatum*	*apyrenum*	14092	38,903	bivalve unident.		1,10	-1,79	B	115	0,72	Korte & Hesselbo 2011[2]
base *hawskerense* sz	Pli			14150	39,000								
Hood 418	Pli	*spinatum*	*hawskerense*	14566	39,876	brachiopod	altered	0,04	-2,87	B	680	0,69	Korte & Hesselbo 2011[2]
Hood 418	Pli	*spinatum*	*hawskerense*	14566	39,876	oyster	altered	1,08	-1,46	B	782	0,48	Korte & Hesselbo 2011[2]
Hood 420	Pli	*spinatum*	*hawskerense*	14566	39,876	brachiopod		1,04	-2,07	B	306	0,73	Korte & Hesselbo 2011[2]
Hood 420	Pli	*spinatum*	*hawskerense*	14566	39,876	brachiopod	altered	0,48	-2,51	B	489	0,73	Korte & Hesselbo 2011[2]
Hood 421	Pli	*spinatum*	*hawskerense*	14566	39,876	pectinid		1,37	-1,60	B	500	0,81	Korte & Hesselbo 2011[2]
Hood 423	Pli	*spinatum*	*hawskerense*	14602	39,952	belemnite		-0,30	-2,29	B	54	1,60	Korte & Hesselbo 2011[2]
Hood 424	Pli	*spinatum*	*hawskerense*	14609	39,966	belemnite		1,17	-3,03	B	59	2,23	Korte & Hesselbo 2011[2]
base *tenuicostatum* z	Toa			14625	40,000								
base *paltum* sz	Toa			14625	40,000								
Hood 433	Toa	*tenuicostatum*	*paltum*	14670	40,098	belemnite		1,65	-1,26	B	56	1,18	Korte & Hesselbo 2011[2]
Hood 434 B	Toa	*tenuicostatum*	*paltum*	14696	40,154	belemnite		1,28	-0,36	B	17	1,12	Korte & Hesselbo 2011[2]
Hood 434 B	Toa	*tenuicostatum*	*paltum*	14696	40,154	belemnite		1,33	-0,42	B	49	1,27	Korte & Hesselbo 2011[2]
Hood 436	Toa	*tenuicostatum*	*paltum*	14696	40,154	belemnite		1,92	-0,54	B	24	1,15	Korte & Hesselbo 2011[2]
Hood 436	Toa	*tenuicostatum*	*paltum*	14696	40,154	belemnite		1,15	-0,87	B	66	1,12	Korte & Hesselbo 2011[2]
Hood 435	Toa	*tenuicostatum*	*paltum*	14704	40,172	belemnite		1,43	-0,63	B	59	1,33	Korte & Hesselbo 2011[2]
Hood 435	Toa	*tenuicostatum*	*paltum*	14704	40,172	belemnite		0,91	-1,12	B	66	1,35	Korte & Hesselbo 2011[2]
Hood 435	Toa	*tenuicostatum*	*paltum*	14704	40,172	belemnite		1,28	-0,94	B	105	1,32	Korte & Hesselbo 2011[2]
Hood 434	Toa	*tenuicostatum*	*paltum*	14748	40,267	belemnite		0,85	-0,92	B	39	1,50	Korte & Hesselbo 2011[2]

Hood 437	Toa	*tenuicostatum*	*paltum*	15065	40,957	belemnite		1,95	-1,35	B		56	1,50	Korte & Hesselbo 2011[2]
base *clevelandicum* sz	Toa			15085	41,000									
Hood 438	Toa	*tenuicostatum*	*clevelandicum*	15106	41,136	belemnite		3,31	0,09	B		76	1,92	Korte & Hesselbo 2011[2]
base *semicelatum* sz	Toa			15452	43,000									
HOOD 439	Toa	*tenuicostatum*	*semicelatum*	15545	43,165	belemnite		2,57	-0,21	B		30	1,49	Korte & Hesselbo 2011[2]
HOOD 440	Toa	*tenuicostatum*	*semicelatum*	15550	43,174	belemnite		3,27	0,16	B		30	1,51	Korte & Hesselbo 2011[2]
HOOD 441	Toa	*tenuicostatum*	*semicelatum*	15562	43,195	belemnite		2,58	-0,53	B		22	1,35	Korte & Hesselbo 2011[2]
HOOD 442	Toa	*tenuicostatum*	*semicelatum*	15632	43,319	belemnite		3,00	-0,53	B		27	1,49	Korte & Hesselbo 2011[2]
HOOD 417	Toa	*tenuicostatum*	*semicelatum*	16648	44,931	pectinid		1,90	-2,56	B		585	0,71	Korte & Hesselbo 2011[2]
HOOD 417 B	Toa	*tenuicostatum*	*semicelatum*	16648	44,931	pectinid		1,78	-2,01	B		544	0,88	Korte & Hesselbo 2011[2]

Staithes

base *margaritatus* z	Pli			-1612	35,000									
base *stokesi* sz	Pli			-1612	35,000									
Stai 115	Pli	*margaritatus*	*stokesi*	-112	35,789	belemnite		1,25	-2,27	CPH		138	1,29	Korte & Hesselbo 2011[2]
Stai 104	Pli	*margaritatus*	*stokesi*	4	35,850	belemnite		1,02	0,25	CPH		165	1,39	Korte & Hesselbo 2011[2]
Stai 113	Pli	*margaritatus*	*stokesi*	8	35,852	pectinid	altered	2,02	-2,96	CPH		2323	0,73	Korte & Hesselbo 2011[2]
Stai 112	Pli	*margaritatus*	*stokesi*	8	35,852	oyster		3,14	-0,93	CPH		555	0,84	Korte & Hesselbo 2011[2]
Stai 112	Pli	*margaritatus*	*stokesi*	8	35,852	oyster		3,93	-0,43	CPH		760	0,72	Korte & Hesselbo 2011[2]
Stai 111	Pli	*margaritatus*	*stokesi*	8	35,852	belemnite		3,05	-0,90	CPH		318	1,64	Korte & Hesselbo 2011[2]
Stai 105	Pli	*margaritatus*	*stokesi*	10	35,853	belemnite		2,50	-1,78	CPH		340	1,64	Korte & Hesselbo 2011[2]
Stai 106	Pli	*margaritatus*	*stokesi*	60	35,879	belemnite		3,48	-2,56	CPH		209	1,58	Korte & Hesselbo 2011[2]
Stai 99	Pli	*margaritatus*	*stokesi*	231	35,969	pectinid		3,01	-0,92	CPH		1034	0,77	Korte & Hesselbo 2011[2]
Stai 100	Pli	*margaritatus*	*stokesi*	233	35,970	pectinid		3,01	-1,09	CPH		582	0,94	Korte & Hesselbo 2011[2]
base *subnodosus* sz	Pli			290	36,000									
Stai 96	Pli	*margaritatus*	*subnodosus*	294	36,006	belemnite	altered	2,87	-2,51	CPH		471	2,28	Korte & Hesselbo 2011[2]
Stai 95	Pli	*margaritatus*	*subnodosus*	294	36,006	pectinid		2,47	-1,84	CPH		454	1,06	Korte & Hesselbo 2011[2]
Stai 97	Pli	*margaritatus*	*subnodosus*	297	36,010	belemnite		1,77	-2,67	CPH		577	2,18	Korte & Hesselbo 2011[2]
Stai 90	Pli	*margaritatus*	*subnodosus*	297	36,010	pectinid		2,74	-0,48	CPH		832	1,27	Korte & Hesselbo 2011[2]
Stai 93	Pli	*margaritatus*	*subnodosus*	355	36,090	bivalve unident.		2,70	-1,14	CPH		859	1,25	Korte & Hesselbo 2011[2]
Stai 83	Pli	*margaritatus*	*subnodosus*	355	36,090	pectinid		2,02	-2,60	CPH		830	1,07	Korte & Hesselbo 2011[2]
Stai 82	Pli	*margaritatus*	*subnodosus*	355	36,090	oyster		3,29	-0,26	CPH		158	1,22	Korte & Hesselbo 2011[2]
Stai 81	Pli	*margaritatus*	*subnodosus*	355	36,090	belemnite		1,88	-2,31	CPH		85	1,82	Korte & Hesselbo 2011[2]
Stai 86	Pli	*margaritatus*	*subnodosus*	371	36,113	belemnite		3,06	-2,01	CPH		59	1,87	Korte & Hesselbo 2011[2]
Stai 84	Pli	*margaritatus*	*subnodosus*	371	36,113	belemnite		2,49	-1,06	CPH		77	1,90	Korte & Hesselbo 2011[2]
Stai 88	Pli	*margaritatus*	*subnodosus*	588	36,414	belemnite		2,42	-2,48	CPH		172	2,08	Korte & Hesselbo 2011[2]
Stai 89	Pli	*margaritatus*	*subnodosus*	770	36,667	bivalve unident.		2,46	-1,70	CPH		1445	1,08	Korte & Hesselbo 2011[2]
Stai 75	Pli	*margaritatus*	*subnodosus*	770	36,667	belemnite		2,46	-1,78	CPH		68	2,01	Korte & Hesselbo 2011[2]
Stai 77	Pli	*margaritatus*	*subnodosus*	790	36,694	belemnite		2,56	-1,69	CPH		102	1,95	Korte & Hesselbo 2011[2]
Stai 73	Pli	*margaritatus*	*subnodosus*	860	36,792	bivalve unident.		2,93	-1,46	CPH		1110	1,31	Korte & Hesselbo 2011[2]
Stai 72	Pli	*margaritatus*	*subnodosus*	860	36,792	oyster		2,66	-1,02	CPH		256	1,26	Korte & Hesselbo 2011[2]
Stai 72	Pli	*margaritatus*	*subnodosus*	860	36,792	oyster		2,53	-1,31	CPH		269	1,11	Korte & Hesselbo 2011[2]
base Avicula Seam	Pli	*margaritatus*	*subnodosus*	890	36,833									
Stai 71	Pli	*margaritatus*	*subnodosus*	890	36,833	bivalve unident.		3,24	-1,41	CPH		455	0,67	Korte & Hesselbo 2011[2]
Stai 71	Pli	*margaritatus*	*subnodosus*	890	36,833	brachiopod		1,93	-1,79	CPH		810	0,85	Korte & Hesselbo 2011[2]
Stai 67	Pli	*margaritatus*	*subnodosus*	890	36,833	pectinid	altered	1,98	-3,09	CPH		2255	0,96	Korte & Hesselbo 2011[2]
Stai 69	Pli	*margaritatus*	*subnodosus*	893	36,838	oyster		3,95	-0,44	CPH		472	1,19	Korte & Hesselbo 2011[2]
Stai 70	Pli	*margaritatus*	*subnodosus*	893	36,838	belemnite		2,82	-1,75	CPH		80	2,09	Korte & Hesselbo 2011[2]
Stai 70	Pli	*margaritatus*	*subnodosus*	893	36,838	pectinid		2,78	-1,31	CPH		1269	1,23	Korte & Hesselbo 2011[2]
Stai 64	Pli	*margaritatus*	*subnodosus*	950	36,917	oyster		2,69	-0,37	CPH		409	1,07	Korte & Hesselbo 2011[2]
Stai 65	Pli	*margaritatus*	*subnodosus*	988	36,969	oyster		3,07	-0,70	CPH		344	1,10	Korte & Hesselbo 2011[2]
Stai 61	Pli	*margaritatus*	*subnodosus*	988	36,969	oyster		3,65	0,09	CPH		136	1,04	Korte & Hesselbo 2011[2]
Stai 61	Pli	*margaritatus*	*subnodosus*	988	36,969	oyster		3,62	-0,06	CPH		253	1,11	Korte & Hesselbo 2011[2]
Stai 62	Pli	*margaritatus*	*subnodosus*	990	36,972	belemnite		2,38	-0,81	CPH		87	2,12	Korte & Hesselbo 2011[2]
Stai 62	Pli	*margaritatus*	*subnodosus*	990	36,972	oyster		3,51	-0,11	CPH		188	1,10	Korte & Hesselbo 2011[2]
Stai 62	Pli	*margaritatus*	*subnodosus*	990	36,972	oyster		3,33	-0,24	CPH		312	0,99	Korte & Hesselbo 2011[2]
Stai 60	Pli	*margaritatus*	*subnodosus*	1005	36,993	oyster		3,76	-0,32	CPH		577	1,16	Korte & Hesselbo 2011[2]
Stai 60	Pli	*margaritatus*	*subnodosus*	1005	36,993	oyster	altered	3,65	-0,41	CPH		1390	0,98	Korte & Hesselbo 2011[2]
base *gibbosus* sz	Pli			1010	37,000									
Stai 57	Pli	*margaritatus*	*gibbosus*	1027	37,018	oyster	altered	2,72	0,03	CPH		2168	1,36	Korte & Hesselbo 2011[2]
Stai 57	Pli	*margaritatus*	*gibbosus*	1027	37,018	oyster	altered	2,67	-0,16	CPH		2803	1,62	Korte & Hesselbo 2011[2]
Stai 54	Pli	*margaritatus*	*gibbosus*	1027	37,018	belemnite		4,33	-1,93	CPH		44	1,92	Korte & Hesselbo 2011[2]
Stai 53	Pli	*margaritatus*	*gibbosus*	1027	37,018	pectinid		3,92	0,02	CPH		1045	1,24	Korte & Hesselbo 2011[2]
Stai 55	Pli	*margaritatus*	*gibbosus*	1027	37,018	pectinid		3,92	-0,33	CPH		952	1,03	Korte & Hesselbo 2011[2]
Stai 58	Pli	*margaritatus*	*gibbosus*	1060	37,054	pectinid		2,58	-1,38	CPH		848	1,09	Korte & Hesselbo 2011[2]
Stai 52	Pli	*margaritatus*	*gibbosus*	1080	37,075	pectinid	altered	3,35	-2,65	CPH		1758	1,35	Korte & Hesselbo 2011[2]
Stai 49	Pli	*margaritatus*	*gibbosus*	1095	37,091	belemnite		1,52	-0,72	CPH		39	1,55	Korte & Hesselbo 2011[2]
STS 229	Pli	*margaritatus*	*gibbosus*	1198	37,202	belemnite		1,46	-2,59	CPH	12,4	81	1,87	this study
Stai 46	Pli	*margaritatus*	*gibbosus*	1364	37,381	pectinid		3,59	-1,97	CPH		789	1,03	Korte & Hesselbo 2011[2]
Stai 44	Pli	*margaritatus*	*gibbosus*	1468	37,492	oyster		3,15	-2,60	CPH		159	1,11	Korte & Hesselbo 2011[2]
Stai 44	Pli	*margaritatus*	*gibbosus*	1468	37,492	oyster		2,81	-2,99	CPH		304	1,67	Korte & Hesselbo 2011[2]
Stai 44	Pli	*margaritatus*	*gibbosus*	1468	37,492	oyster	altered	3,24	-2,20	CPH		803	1,35	Korte & Hesselbo 2011[2]
Stai 44	Pli	*margaritatus*	*gibbosus*	1468	37,492	pectinid		2,95	-1,34	CPH		790	1,39	Korte & Hesselbo 2011[2]
Stai 44	Pli	*margaritatus*	*gibbosus*	1468	37,492	pectinid		2,74	-1,86	CPH		1019	1,37	Korte & Hesselbo 2011[2]
Stai 44	Pli	*margaritatus*	*gibbosus*	1468	37,492	pectinid		2,56	-1,70	CPH		1413	1,83	Korte & Hesselbo 2011[2]
Stai 43	Pli	*margaritatus*	*gibbosus*	1470	37,495	belemnite		3,21	-3,34	CPH		106	2,14	Korte & Hesselbo 2011[2]
Stai 40	Pli	*margaritatus*	*gibbosus*	1470	37,495	pectinid		2,01	-2,85	CPH		333	1,18	Korte & Hesselbo 2011[2]

Stai 40	Pli	*margaritatus*	*gibbosus*	1470	37,495	pectinid			2,19	-2,75	CPH		442	1,16	Korte & Hesselbo 2011[2]
Stai 40	Pli	*margaritatus*	*gibbosus*	1470	37,495	pectinid			2,17	-2,65	CPH		501	1,37	Korte & Hesselbo 2011[2]
Stai 38	Pli	*margaritatus*	*gibbosus*	1482	37,508	belemnite			3,01	-2,11	CPH		154	2,05	Korte & Hesselbo 2011[2]
Stai 38	Pli	*margaritatus*	*gibbosus*	1482	37,508	pectinid			2,42	-1,97	CPH		603	1,38	Korte & Hesselbo 2011[2]
Stai 42	Pli	*margaritatus*	*gibbosus*	1485	37,511	pectinid			2,09	-1,68	CPH		458	1,73	Korte & Hesselbo 2011[2]
Stai 41	Pli	*margaritatus*	*gibbosus*	1485	37,511	oyster			3,92	-1,09	CPH		150	1,10	Korte & Hesselbo 2011[2]
Stai 41	Pli	*margaritatus*	*gibbosus*	1485	37,511	oyster			3,49	-1,28	CPH		165	1,11	Korte & Hesselbo 2011[2]
Stai 36	Pli	*margaritatus*	*gibbosus*	1500	37,527	pectinid			3,03	-1,30	CPH		185	0,98	Korte & Hesselbo 2011[2]
Stai 36	Pli	*margaritatus*	*gibbosus*	1500	37,527	pectinid			2,72	-2,23	CPH		216	1,04	Korte & Hesselbo 2011[2]
base Raisdale Seam	Pli	*margaritatus*	*gibbosus*	1510	37,538										
Stai 35	Pli	*margaritatus*	*gibbosus*	1510	37,538	oyster			4,17	0,53	CPH		511	1,79	Korte & Hesselbo 2011[2]
Stai 35	Pli	*margaritatus*	*gibbosus*	1510	37,538	oyster			4,52	0,04	CPH		626	1,65	Korte & Hesselbo 2011[2]
Stai 34	Pli	*margaritatus*	*gibbosus*	1515	37,543	belemnite			3,37	-1,98	CPH		92	2,02	Korte & Hesselbo 2011[2]
Stai 33	Pli	*margaritatus*	*gibbosus*	1517	37,545	oyster			3,44	-1,05	CPH		480	1,36	Korte & Hesselbo 2011[2]
Stai 33	Pli	*margaritatus*	*gibbosus*	1517	37,545	oyster			3,32	-1,03	CPH		490	1,54	Korte & Hesselbo 2011[2]
Stai 25	Pli	*margaritatus*	*gibbosus*	1519	37,547	pectinid			1,85	-1,55	CPH		1122	1,21	Korte & Hesselbo 2011[2]
Stai 32	Pli	*margaritatus*	*gibbosus*	1538	37,568	belemnite			3,06	-2,41	CPH		249	2,01	Korte & Hesselbo 2011[2]
Stai 26	Pli	*margaritatus*	*gibbosus*	1543	37,573	belemnite			2,13	-2,76	CPH		166	2,04	Korte & Hesselbo 2011[2]
STS 217	Pli	*margaritatus*	*gibbosus*	1584	37,617	belemnite			1,63	-1,36	CPH	11,3	56	1,74	this study
STS 215	Pli	*margaritatus*	*gibbosus*	1604	37,638	belemnite			0,96	-0,86	CPH	10,6	33	1,84	this study
STS 220	Pli	*margaritatus*	*gibbosus*	1617	37,652	belemnite			3,04	-1,98	CPH	13,2	59	1,89	this study
STS 212	Pli	*margaritatus*	*gibbosus*	1631	37,668	belemnite			1,93	-2,04	CPH	11,7	142	1,70	this study
STS 212	Pli	*margaritatus*	*gibbosus*	1631	37,668	belemnite			1,91	-2,00	CPH	12,8	131	1,75	this study
Stai 39	Pli	*margaritatus*	*gibbosus*	1640	37,677	belemnite			1,36	-3,66	CPH		262	2,31	Korte & Hesselbo 2011[2]
Stai 24	Pli	*margaritatus*	*gibbosus*	1646	37,684	belemnite			1,88	-0,71	CPH		104	1,65	Korte & Hesselbo 2011[2]
STS 210	Pli	*margaritatus*	*gibbosus*	1725	37,768	pectinid		altered	0,56	-3,70	CPH	3,3	1184	1,03	this study
base Two-Foot Seam	Pli	*margaritatus*	*gibbosus*	1828	37,880										
Stai 20	Pli	*margaritatus*	*gibbosus*	1828	37,880	oyster			3,25	-0,50	CPH		450	0,88	Korte & Hesselbo 2011[2]
Stai 19	Pli	*margaritatus*	*gibbosus*	1830	37,882	belemnite			2,46	-1,76	CPH		169	1,47	Korte & Hesselbo 2011[2]
Stai 18	Pli	*margaritatus*	*gibbosus*	1830	37,882	bivalve unident.			4,09	-1,15	CPH		1232	1,68	Korte & Hesselbo 2011[2]
Stai 16	Pli	*margaritatus*	*gibbosus*	1830	37,882	bivalve unident.			3,43	-1,00	CPH		1025	1,81	Korte & Hesselbo 2011[2]
Stai 15	Pli	*margaritatus*	*gibbosus*	1837	37,889	belemnite			2,40	-1,73	CPH		79	1,55	Korte & Hesselbo 2011[2]
Stai 12	Pli	*margaritatus*	*gibbosus*	1852	37,905	belemnite			1,66	-2,01	CPH		77	2,01	Korte & Hesselbo 2011[2]
Stai 14	Pli	*margaritatus*	*gibbosus*	1852	37,905	Plagiostoma			4,63	-0,24	CPH		381	1,31	Korte & Hesselbo 2011[2]
Stai 13	Pli	*margaritatus*	*gibbosus*	1868	37,923	belemnite			1,74	-2,54	CPH		105	1,64	Korte & Hesselbo 2011[2]
Stai 9	Pli	*margaritatus*	*gibbosus*	1875	37,930	pectinid			2,72	-1,55	CPH		349	1,08	Korte & Hesselbo 2011[2]
Stai 1	Pli	*margaritatus*	*gibbosus*	1915	37,973	belemnite			0,39	0,44	CPH		105	0,93	Korte & Hesselbo 2011[2]
Stai 2	Pli	*margaritatus*	*gibbosus*	1919	37,977	brachiopod			2,75	-0,99	CPH		294	1,10	Korte & Hesselbo 2011[2]
Stai 3	Pli	*margaritatus*	*gibbosus*	1920	37,978	pectinid			1,95	-1,22	CPH		945	1,40	Korte & Hesselbo 2011[2]
base Pecten Seam	Pli			1940	38,000										
base *spinatum* z	Pli			1940	38,000										
base *apyrenum* sz	Pli			1940	38,000										Korte & Hesselbo 2011[2]
Stai 5	Pli	*spinatum*	*apyrenum*	1955	38,036	belemnite			2,44	-1,89	CPH		159	1,79	Korte & Hesselbo 2011[2]
Stai 5	Pli	*spinatum*	*apyrenum*	1955	38,036	bivalve unident.			3,97	-1,95	CPH		614	1,10	Korte & Hesselbo 2011[2]
Stai 6	Pli	*spinatum*	*apyrenum*	1970	38,073	belemnite			2,53	-1,19	CPH		67	1,60	Korte & Hesselbo 2011[2]
Stai 10	Pli	*spinatum*	*apyrenum*	1970	38,073	bivalve unident.			2,49	-1,34	CPH		449	1,18	Korte & Hesselbo 2011[2]
STS 205	Pli	*spinatum*	*apyrenum*	2048	38,262	pectinid			3,29	-0,73	CPH	3,1	1266	1,03	this study
STS 205	Pli	*spinatum*	*apyrenum*	2048	38,262	pectinid			3,23	-1,03	CPH	0,5	1245	1,00	this study
STS 205	Pli	*spinatum*	*apyrenum*	2048	38,262	pectinid			3,10	-1,23	CPH	3,5	1392	1,07	this study
STS 205	Pli	*spinatum*	*apyrenum*	2048	38,262	belemnite			2,66	-0,89	CPH	17,7	473	1,71	this study
STS 207	Pli	*spinatum*	*apyrenum*	2063	38,297	belemnite			2,22	-1,35	CPH	15,7	66	1,92	this study
STS 206	Pli	*spinatum*	*apyrenum*	2063	38,297	belemnite			3,35	-1,29	CPH	8,6	81	1,62	this study
STS 204	Pli	*spinatum*	*apyrenum*	2179	38,578	belemnite		altered	-0,29	-0,50	CPH	9,8	387	1,33	this study
STS 204	Pli	*spinatum*	*apyrenum*	2179	38,578	belemnite		altered	-0,57	-1,19	CPH	7,0	479	1,32	this study
STS 203	Pli	*spinatum*	*apyrenum*	2238	38,722	belemnite		altered	1,22	-2,54	CPH	15,9	444	1,83	this study
STS 202	Pli	*spinatum*	*apyrenum*	2321	38,923	belemnite			1,11	-2,11	CPH	12,2	85	1,89	this study
STS 202	Pli	*spinatum*	*apyrenum*	2321	38,923	belemnite			1,20	-2,28	CPH	17,1	115	1,88	this study
STS 201	Pli	*spinatum*	*apyrenum*	2352	38,998	oyster			1,41	-1,70	CPH	1,9	562	0,70	this study
STS 201	Pli	*spinatum*	*apyrenum*	2352	38,998	oyster			1,62	-1,59	CPH	0,3	520	0,70	this study
STS 201	Pli	*spinatum*	*apyrenum*	2352	38,998	pectinid			2,81	-1,17	CPH	4,2	549	0,98	this study
STS 201	Pli	*spinatum*	*apyrenum*	2352	38,998	belemnite			1,13	-2,11	CPH	12,9	126	1,65	this study
STS 201	Pli	*spinatum*	*apyrenum*	2352	38,998	belemnite			1,18	-2,06	CPH	12,6	72	1,63	this study
base *hawskerense* sz	Pli			2353	39,000										
ST bel B	Pli	*spinatum*	*hawskerense*	2450	39,397	belemnite			2,38	-2,92	CPH	13,3	79	2,18	this study
ST bel A	Pli	*spinatum*	*hawskerense*	2568	39,876	belemnite			1,63	-1,64	CPH	11,1	393	1,82	this study
base *tenuicostatum* z	Toa			2598											
base *paltum* sz	Toa			2598											

Hawsker Bottoms

base Toarcian	Toa			-53											
base *tenuicostatum* z	Toa			-53											
base *paltum* sz	Toa			-53											
base Grey Shales Mb	Toa			4											
Haws_MR_001	Toa	*tenuicostatum*	*paltum*	151	40,432	belemnite	*Passaloteuthis*		1,14	-1,14	OX	10,0	23	1,57	Ullmann et al. 2014[3]
Haws_MR_001	Toa	*tenuicostatum*	*paltum*	151	40,432	belemnite	*Passaloteuthis*		1,24	-1,03	OX	8,5	44	1,42	Ullmann et al. 2014[3]
Haws_MR_001	Toa	*tenuicostatum*	*paltum*	151	40,432	belemnite	*Passaloteuthis*		1,64	-0,84	OX	8,7	58	1,46	Ullmann et al. 2014[3]
Haws_CK_011	Toa	*tenuicostatum*	*paltum*	228	40,595	belemnite	*Passaloteuthis*		1,07	-1,00	OX	9,7	18	1,39	Ullmann et al. 2014[3]

Haws_CK_011	Toa	*tenuicostatum*	*paltum*	228	40,595	belemnite	*Passaloteuthis*	0,53	-0,87	OX	9,5	39	1,32	Ullmann et al. 2014[3]
Haws_CK_011	Toa	*tenuicostatum*	*paltum*	228	40,595	belemnite	*Passaloteuthis*	1,79	-0,71	OX	9,2	44	1,34	Ullmann et al. 2014[3]
Haws_CK_012	Toa	*tenuicostatum*	*paltum*	275	40,695	belemnite	*Passaloteuthis*	1,76	-1,01	OX	9,6	26	1,43	Ullmann et al. 2014[3]
Haws_CK_012	Toa	*tenuicostatum*	*paltum*	275	40,695	belemnite	*Passaloteuthis*	1,68	-0,87	OX	8,7	23	1,44	Ullmann et al. 2014[3]
Haws_CK_012	Toa	*tenuicostatum*	*paltum*	275	40,695	belemnite	*Passaloteuthis*	1,09	-1,10	OX	8,7	49	1,53	Ullmann et al. 2014[3]
Haws_CK_008	Toa	*tenuicostatum*	*paltum*	352	40,858	belemnite	*Passaloteuthis*	2,84	-0,71	OX	7,9	10	1,37	Ullmann et al. 2014[3]
Haws_CK_008	Toa	*tenuicostatum*	*paltum*	352	40,858	belemnite	*Passaloteuthis*	2,82	-0,53	OX	8,4	5	1,37	Ullmann et al. 2014[3]
Haws_CK_008	Toa	*tenuicostatum*	*paltum*	352	40,858	belemnite	*Passaloteuthis*	1,42	-0,84	OX	9,0	46	1,47	Ullmann et al. 2014[3]
Haws_CK_007	Toa	*tenuicostatum*	*paltum*	359	40,873	belemnite	*Passaloteuthis*	2,94	-0,92	OX	13,6	31	1,56	Ullmann et al. 2014[3]
Haws_CK_007	Toa	*tenuicostatum*	*paltum*	359	40,873	belemnite	*Passaloteuthis*	2,44	-0,59	OX	9,9	10	1,32	Ullmann et al. 2014[3]
Haws_CK_007	Toa	*tenuicostatum*	*paltum*	359	40,873	belemnite	*Passaloteuthis*	1,55	-1,31	OX	9,3	68	1,39	Ullmann et al. 2014[3]
Haws_CK_003	Toa	*tenuicostatum*	*paltum*	395	40,949	belemnite	*Passaloteuthis*	2,58	-0,92	OX	10,9	32	1,44	Ullmann et al. 2014[3]
Haws_CK_003	Toa	*tenuicostatum*	*paltum*	395	40,949	belemnite	*Passaloteuthis*	2,19	-0,88	OX	10,0	39	1,37	Ullmann et al. 2014[3]
Haws_CK_003	Toa	*tenuicostatum*	*paltum*	395	40,949	belemnite	*Passaloteuthis*	3,40	-0,61	OX	10,3	51	1,37	Ullmann et al. 2014[3]
Haws_CK_014	Toa	*tenuicostatum*	*paltum*	395	40,949	belemnite	*Passaloteuthis*	1,94	-1,40	OX	10,8	32	1,85	Ullmann et al. 2014[3]
Haws_CK_014	Toa	*tenuicostatum*	*paltum*	395	40,949	belemnite	*Passaloteuthis*	2,88	-0,55	OX	10,5	14	1,94	Ullmann et al. 2014[3]
Haws_CK_002	Toa	*tenuicostatum*	*paltum*	416	40,994	belemnite	*Passaloteuthis*	2,32	-0,70	OX	9,4	22	1,37	Ullmann et al. 2014[3]
Haws_CK_002	Toa	*tenuicostatum*	*paltum*	416	40,994	belemnite	*Passaloteuthis*	1,41	-0,88	OX	9,5	30	1,25	Ullmann et al. 2014[3]
Haws_CK_002	Toa	*tenuicostatum*	*paltum*	416	40,994	belemnite	*Passaloteuthis*	2,59	-0,73	OX	9,3	29	1,31	Ullmann et al. 2014[3]
base clevelandicum sz	Toa			419										
Haws_CK_001	Toa	*tenuicostatum*	*clevelandicum*	447	41,292	belemnite	*Passaloteuthis*	1,62	-0,98	OX	9,4	11	1,27	Ullmann et al. 2014[3]
Haws_CK_001	Toa	*tenuicostatum*	*clevelandicum*	447	41,292	belemnite	*Passaloteuthis*	1,99	-1,15	OX	10,3	11	1,23	Ullmann et al. 2014[3]
Haws_CK_001	Toa	*tenuicostatum*	*clevelandicum*	447	41,292	belemnite	*Passaloteuthis*	1,77	-1,34	OX	9,2	29	1,24	Ullmann et al. 2014[3]
Haws_CK_004	Toa	*tenuicostatum*	*clevelandicum*	459	41,417	belemnite	*Passaloteuthis*	2,16	-0,94	OX	10,1	16	1,52	Ullmann et al. 2014[3]
Haws_CK_004	Toa	*tenuicostatum*	*clevelandicum*	459	41,417	belemnite	*Passaloteuthis*	2,62	-0,77	OX	9,5	18	1,51	Ullmann et al. 2014[3]
Haws_CK_004	Toa	*tenuicostatum*	*clevelandicum*	459	41,417	belemnite	*Passaloteuthis*	1,80	-0,82	OX	11,3	10	1,55	Ullmann et al. 2014[3]
Haws_CK_015	Toa	*tenuicostatum*	*clevelandicum*	461	41,438	belemnite	*Passaloteuthis*	1,74	-1,09	OX	8,6	11	1,45	Ullmann et al. 2014[3]
Haws_CK_015	Toa	*tenuicostatum*	*clevelandicum*	461	41,438	belemnite	*Passaloteuthis*	2,13	-1,05	OX	7,7	20	1,45	Ullmann et al. 2014[3]
Haws_CK_015	Toa	*tenuicostatum*	*clevelandicum*	461	41,438	belemnite	*Passaloteuthis*	2,21	-1,14	OX	8,3	22	1,52	Ullmann et al. 2014[3]
Haws_CK_028	Toa	*tenuicostatum*	*clevelandicum*	475	41,583	belemnite	*Passaloteuthis*	3,09	-1,04	OX	10,4	5	1,66	Ullmann et al. 2014[3]
Haws_CK_028	Toa	*tenuicostatum*	*clevelandicum*	475	41,583	belemnite	*Passaloteuthis*	1,71	-0,91	OX	10,4	5	1,62	Ullmann et al. 2014[3]
Haws_CK_028	Toa	*tenuicostatum*	*clevelandicum*	475	41,583	belemnite	*Passaloteuthis*	1,87	-0,77	OX	11,1	7	1,77	Ullmann et al. 2014[3]
Haws_CK_027	Toa	*tenuicostatum*	*clevelandicum*	478	41,615	belemnite	*Passaloteuthis*	2,55	-0,88	OX	10,6	11	1,50	Ullmann et al. 2014[3]
Haws_CK_027	Toa	*tenuicostatum*	*clevelandicum*	478	41,615	belemnite	*Passaloteuthis*	2,20	-0,87	OX	10,6	23	1,36	Ullmann et al. 2014[3]
Haws_CK_027	Toa	*tenuicostatum*	*clevelandicum*	478	41,615	belemnite	*Passaloteuthis*	2,49	-0,72	OX	10,5	19	1,43	Ullmann et al. 2014[3]
Haws_CK_017	Toa	*tenuicostatum*	*clevelandicum*	488	41,719	belemnite	*Passaloteuthis*	3,16	-0,40	OX	9,1	10	1,50	Ullmann et al. 2014[3]
Haws_CK_017	Toa	*tenuicostatum*	*clevelandicum*	488	41,719	belemnite	*Passaloteuthis*	3,58	-0,59	OX	8,4	6	1,43	Ullmann et al. 2014[3]
Haws_CK_017	Toa	*tenuicostatum*	*clevelandicum*	488	41,719	belemnite	*Passaloteuthis*	3,57	-0,10	OX	7,7	45	1,40	Ullmann et al. 2014[3]
Haws_CK_005	Toa	*tenuicostatum*	*clevelandicum*	510	41,948	belemnite	*Passaloteuthis*	3,30	-1,49	OX	9,6	16	1,44	Ullmann et al. 2014[3]
Haws_CK_005	Toa	*tenuicostatum*	*clevelandicum*	510	41,948	belemnite	*Passaloteuthis*	3,04	-1,24	OX	10,4	22	1,46	Ullmann et al. 2014[3]
Haws_CK_005	Toa	*tenuicostatum*	*clevelandicum*	510	41,948	belemnite	*Passaloteuthis*	1,82	-1,34	OX	14,1	52	1,60	Ullmann et al. 2014[3]
base *tenuicostatum* sz	Toa			515										
Haws_MEJ_008	Toa	*tenuicostatum*	*tenuicostatum*	516	42,004	belemnite	*Passaloteuthis*	2,64	-0,75	OX	9,6	20	1,46	Ullmann et al. 2014[3]
Haws_MEJ_008	Toa	*tenuicostatum*	*tenuicostatum*	516	42,004	belemnite	*Passaloteuthis*	2,80	-0,92	OX	9,8	18	1,37	Ullmann et al. 2014[3]
Haws_MEJ_007	Toa	*tenuicostatum*	*tenuicostatum*	516	42,004	belemnite	*Passaloteuthis*	2,23	-0,99	OX	9,7	19	1,27	Ullmann et al. 2014[3]
Haws_MEJ_007	Toa	*tenuicostatum*	*tenuicostatum*	516	42,004	belemnite	*Passaloteuthis*	2,23	-0,61	OX	12,8	34	1,27	Ullmann et al. 2014[3]
Haws_MEJ_007	Toa	*tenuicostatum*	*tenuicostatum*	516	42,004	belemnite	*Passaloteuthis*	2,57	-1,17	OX	9,6	31	1,31	Ullmann et al. 2014[3]
Haws_MEJ_005	Toa	*tenuicostatum*	*tenuicostatum*	516	42,004	belemnite	*Passaloteuthis*	2,44	-0,89	OX	10,4	14	1,53	Ullmann et al. 2014[3]
Haws_MEJ_005	Toa	*tenuicostatum*	*tenuicostatum*	516	42,004	belemnite	*Passaloteuthis*	1,71	-0,84	OX	9,5	10	1,42	Ullmann et al. 2014[3]
Haws_MEJ_005	Toa	*tenuicostatum*	*tenuicostatum*	516	42,004	belemnite	*Passaloteuthis*	2,49	-0,94	OX	9,9	33	1,65	Ullmann et al. 2014[3]
Haws_MEJ_003	Toa	*tenuicostatum*	*tenuicostatum*	516	42,004	belemnite	*Passaloteuthis*	2,76	-0,75	OX	9,5	15	1,56	Ullmann et al. 2014[3]
Haws_MEJ_003	Toa	*tenuicostatum*	*tenuicostatum*	516	42,004	belemnite	*Passaloteuthis*	2,79	-0,75	OX	9,2	22	1,48	Ullmann et al. 2014[3]
Haws_MEJ_003	Toa	*tenuicostatum*	*tenuicostatum*	516	42,004	belemnite	*Passaloteuthis*	2,03	-0,85	OX	9,4	13	1,48	Ullmann et al. 2014[3]
Haws_CK_016	Toa	*tenuicostatum*	*tenuicostatum*	516	42,004	belemnite	*Passaloteuthis*	2,55	-0,66	OX	7,5	14	1,39	Ullmann et al. 2014[3]
Haws_CK_016	Toa	*tenuicostatum*	*tenuicostatum*	516	42,004	belemnite	*Passaloteuthis*	2,40	-0,55	OX	8,9	80	1,43	Ullmann et al. 2014[3]
Haws_CK_006	Toa	*tenuicostatum*	*tenuicostatum*	518	42,012	belemnite	*Passaloteuthis*	2,81	-1,24	OX	10,3	25	1,62	Ullmann et al. 2014[3]
Haws_CK_006	Toa	*tenuicostatum*	*tenuicostatum*	518	42,012	belemnite	*Passaloteuthis*	3,03	-0,96	OX	9,1	27	1,46	Ullmann et al. 2014[3]
Haws_CK_006	Toa	*tenuicostatum*	*tenuicostatum*	518	42,012	belemnite	*Passaloteuthis*	1,89	-0,94	OX	11,1	29	1,59	Ullmann et al. 2014[3]
Haws_CK_025	Toa	*tenuicostatum*	*tenuicostatum*	538	42,089	belemnite	*Passaloteuthis*	3,04	-0,63	OX	12,5	14	1,34	Ullmann et al. 2014[3]
Haws_CK_025	Toa	*tenuicostatum*	*tenuicostatum*	538	42,089	belemnite	*Passaloteuthis*	2,48	-0,66	OX	9,9	16	1,30	Ullmann et al. 2014[3]
Haws_CK_025	Toa	*tenuicostatum*	*tenuicostatum*	538	42,089	belemnite	*Passaloteuthis*	2,97	-0,78	OX	10,2	14	1,39	Ullmann et al. 2014[3]
Haws_CK_026	Toa	*tenuicostatum*	*tenuicostatum*	582	42,259	belemnite	*Passaloteuthis*	1,35	-1,53	OX	10,7	45	1,48	Ullmann et al. 2014[3]
Haws_CK_026	Toa	*tenuicostatum*	*tenuicostatum*	582	42,259	belemnite	*Passaloteuthis*	1,51	-1,20	OX	9,2	54	1,49	Ullmann et al. 2014[3]
Haws_CK_026	Toa	*tenuicostatum*	*tenuicostatum*	582	42,259	belemnite	*Passaloteuthis*	2,45	-1,23	OX	10,1	71	1,73	Ullmann et al. 2014[3]
Haws_CK_030	Toa	*tenuicostatum*	*tenuicostatum*	597	42,317	belemnite	*Passaloteuthis*	4,35	-2,10	OX	10,0	8	2,09	Ullmann et al. 2014[3]
base *semicelatum* sz	Toa			774	43,000									
Haws_NTX	Toa	*tenuicostatum*	*semicelatum*	774	43,000	belemnite	*Passaloteuthis*	1,49	-1,01	OX	9,1	12	1,36	Ullmann et al. 2014[3]
Haws_NTX	Toa	*tenuicostatum*	*semicelatum*	774	43,000	belemnite	*Passaloteuthis*	1,98	-0,94	OX	10,4	20	1,44	Ullmann et al. 2014[3]
Haws_NTX	Toa	*tenuicostatum*	*semicelatum*	774	43,000	belemnite	*Passaloteuthis*	1,65	-0,81	OX	8,7	15	1,40	Ullmann et al. 2014[3]
Haws_CK_037	Toa	*tenuicostatum*	*semicelatum*	789	43,027	belemnite	*Passaloteuthis*	1,59	-2,67	OX	10,4	28	1,37	Ullmann et al. 2014[3]
Haws_CK_037	Toa	*tenuicostatum*	*semicelatum*	789	43,027	belemnite	*Passaloteuthis*	1,61	-1,47	OX	10,1	15	1,33	Ullmann et al. 2014[3]
Haws_CK_037	Toa	*tenuicostatum*	*semicelatum*	789	43,027	belemnite	*Passaloteuthis*	1,71	-1,78	OX	10,1	16	1,37	Ullmann et al. 2014[3]
Haws_CK_036	Toa	*tenuicostatum*	*semicelatum*	789	43,027	belemnite	*Passaloteuthis*	1,68	-1,11	OX	9,7	21	1,56	Ullmann et al. 2014[3]
Haws_CK_036	Toa	*tenuicostatum*	*semicelatum*	789	43,027	belemnite	*Passaloteuthis*	2,35	-0,64	OX	10,1	22	1,46	Ullmann et al. 2014[3]
Haws_CK_036	Toa	*tenuicostatum*	*semicelatum*	789	43,027	belemnite	*Passaloteuthis*	1,97	-0,57	OX	9,7	43	1,36	Ullmann et al. 2014[3]
Haws_CK_039	Toa	*tenuicostatum*	*semicelatum*	855	43,144	belemnite	*Passaloteuthis*	3,05	-0,62	OX	8,9	54	1,74	Ullmann et al. 2014[3]
Haws_CK_039	Toa	*tenuicostatum*	*semicelatum*	855	43,144	belemnite	*Passaloteuthis*	3,30	-0,36	OX	8,6	51	1,76	Ullmann et al. 2014[3]
Haws_CK_039	Toa	*tenuicostatum*	*semicelatum*	855	43,144	belemnite	*Passaloteuthis*	3,08	-0,36	OX	11,2	24	1,91	Ullmann et al. 2014[3]
Haws_CK_043	Toa	*tenuicostatum*	*semicelatum*	885	43,197	belemnite	*Passaloteuthis*	2,34	-0,91	OX	8,2	11	1,65	Ullmann et al. 2014[3]

Haws_CK_043	Toa	*tenuicostatum*	*semicelatum*	885	43,197	belemnite	*Passaloteuthis*	1,98	-1,35	OX	10,4	46	1,73	Ullmann et al. 2014[3]
Haws_CK_043	Toa	*tenuicostatum*	*semicelatum*	885	43,197	belemnite	*Passaloteuthis*	1,60	-1,26	OX	10,3	25	1,72	Ullmann et al. 2014[3]
Haws_CK_042	Toa	*tenuicostatum*	*semicelatum*	890	43,206	belemnite	*Passaloteuthis*	2,84	-0,58	OX	7,6	13	1,26	Ullmann et al. 2014[3]
Haws_CK_042	Toa	*tenuicostatum*	*semicelatum*	890	43,206	belemnite	*Passaloteuthis*	3,04	-0,68	OX	7,9	24	1,29	Ullmann et al. 2014[3]
Haws_CK_044	Toa	*tenuicostatum*	*semicelatum*	893	43,211	belemnite	*Passaloteuthis*	2,56	-0,91	OX	11,5	14	1,89	Ullmann et al. 2014[3]
Haws_CK_044	Toa	*tenuicostatum*	*semicelatum*	893	43,211	belemnite	*Passaloteuthis*	3,25	-0,83	OX	10,6	29	1,99	Ullmann et al. 2014[3]
Haws_CK_041	Toa	*tenuicostatum*	*semicelatum*	895	43,215	belemnite	*Pseudohastites* (?)	2,84	-0,68	OX	11,4	24	1,28	Ullmann et al. 2014[3]
Haws_CK_041	Toa	*tenuicostatum*	*semicelatum*	895	43,215	belemnite	*Pseudohastites* (?)	2,08	-0,94	OX	9,5	36	1,39	Ullmann et al. 2014[3]
Haws_CK_041	Toa	*tenuicostatum*	*semicelatum*	895	43,215	belemnite	*Pseudohastites* (?)	1,51	-1,43	OX	10,1	69	1,48	Ullmann et al. 2014[3]
Haws_CK_045	Toa	*tenuicostatum*	*semicelatum*	983	43,371	belemnite	*Passaloteuthis*	2,15	-1,48	OX	10,3	19	1,62	Ullmann et al. 2014[3]
Haws_CK_045	Toa	*tenuicostatum*	*semicelatum*	983	43,371	belemnite	*Passaloteuthis*	1,77	-1,36	OX	10,1	34	1,61	Ullmann et al. 2014[3]
Haws_CK_045	Toa	*tenuicostatum*	*semicelatum*	983	43,371	belemnite	*Passaloteuthis*	1,79	-0,89	OX	8,9	10	1,55	Ullmann et al. 2014[3]
Haws_CK_046	Toa	*tenuicostatum*	*semicelatum*	1172	43,706	belemnite	*Passaloteuthis* (?)	3,08	-1,12	OX	8,5	53	1,36	Ullmann et al. 2014[3]
Haws_CK_046	Toa	*tenuicostatum*	*semicelatum*	1172	43,706	belemnite	*Passaloteuthis* (?)	2,98	-1,11	OX	8,8	31	1,45	Ullmann et al. 2014[3]
base Jet Rock	Toa			1338										
base *falciferum* z	Toa			1338										
base *exaratum* sz	Toa			1338										
Haws_CK_035	Toa	*falciferum*	*exaratum*	1565	44,334	belemnite	*Acrocoelites*	1,76	-3,89	OX	17,1	38	1,76	Ullmann et al. 2014[3]
Haws_CK_035	Toa	*falciferum*	*exaratum*	1565	44,334	belemnite	*Acrocoelites*	1,40	-3,50	OX	15,0	19	1,82	Ullmann et al. 2014[3]
Haws_CK_035	Toa	*falciferum*	*exaratum*	1565	44,334	belemnite	*Acrocoelites*	1,98	-3,89	OX	13,6	13	2,04	Ullmann et al. 2014[3]
Haws_CK_052	Toa	*falciferum*	*exaratum*	1591	44,373	belemnite	*Acrocoelites*	1,66	-3,83	OX	15,8	22	2,03	Ullmann et al. 2014[3]
Haws_CK_052	Toa	*falciferum*	*exaratum*	1591	44,373	belemnite	*Acrocoelites*	1,44	-3,51	OX	16,2	12	2,15	Ullmann et al. 2014[3]
Haws_CK_052	Toa	*falciferum*	*exaratum*	1591	44,373	belemnite	*Acrocoelites*	1,33	-3,92	OX	17,8	14	2,34	Ullmann et al. 2014[3]
Haws_NT_002	Toa	*falciferum*	*exaratum*	1594	44,377	belemnite	*Acrocoelites*	1,15	-4,45	OX	22,7	44	2,22	Ullmann et al. 2014[3]
Haws_NT_002	Toa	*falciferum*	*exaratum*	1594	44,377	belemnite	*Acrocoelites*	1,07	-3,99	OX	18,3	18	2,08	Ullmann et al. 2014[3]
Haws_NT_002	Toa	*falciferum*	*exaratum*	1594	44,377	belemnite	*Acrocoelites*	1,27	-3,79	OX	15,1	20	2,03	Ullmann et al. 2014[3]
Haws_NT_001	Toa	*falciferum*	*exaratum*	1594	44,377	belemnite	*Acrocoelites* (?)	1,94	-4,56	OX	20,0	29	2,31	Ullmann et al. 2014[3]
Haws_NT_001	Toa	*falciferum*	*exaratum*	1594	44,377	belemnite	*Acrocoelites* (?)	1,89	-4,89	OX	14,8	23	2,07	Ullmann et al. 2014[3]
Haws_NT_001	Toa	*falciferum*	*exaratum*	1594	44,377	belemnite	*Acrocoelites* (?)	1,72	-5,23	OX	15,4	29	1,90	Ullmann et al. 2014[3]
Haws_MEJ_002	Toa	*falciferum*	*exaratum*	1601	44,387	belemnite	*Acrocoelites*	1,14	-4,90	OX	23,3	50	2,21	Ullmann et al. 2014[3]
Haws_MEJ_002	Toa	*falciferum*	*exaratum*	1601	44,387	belemnite	*Acrocoelites*	1,28	-4,65	OX	15,1	60	1,94	Ullmann et al. 2014[3]
Haws_NT_003	Toa	*falciferum*	*exaratum*	1606	44,395	belemnite	*Acrocoelites* (?)	-0,24	-5,78	OX	23,3	64	2,21	Ullmann et al. 2014[3]
Haws_NT_003	Toa	*falciferum*	*exaratum*	1606	44,395	belemnite	*Acrocoelites* (?)	-0,28	-5,63	OX	19,1	22	2,14	Ullmann et al. 2014[3]
Haws_NT_003	Toa	*falciferum*	*exaratum*	1606	44,395	belemnite	*Acrocoelites* (?)	-0,31	-5,59	OX	17,2	30	1,88	Ullmann et al. 2014[3]
Haws_CK_058	Toa	*falciferum*	*exaratum*	1639	44,443	belemnite	*Acrocoelites*	0,54	-4,28	OX	22,8	13	2,45	Ullmann et al. 2014[3]
Haws_CK_058	Toa	*falciferum*	*exaratum*	1639	44,443	belemnite	*Acrocoelites*	0,58	-4,11	OX	21,2	15	2,49	Ullmann et al. 2014[3]
Haws_CK_058	Toa	*falciferum*	*exaratum*	1639	44,443	belemnite	*Acrocoelites*	1,02	-4,07	OX	17,5	28	2,40	Ullmann et al. 2014[3]
Haws_CK_059	Toa	*falciferum*	*exaratum*	1639	44,443	belemnite	*Acrocoelites*	1,48	-4,18	OX	15,8	13	2,24	Ullmann et al. 2014[3]
Haws_CK_059	Toa	*falciferum*	*exaratum*	1639	44,443	belemnite	*Acrocoelites*	1,59	-4,02	OX	14,3	9	2,24	Ullmann et al. 2014[3]
Haws_CK_059	Toa	*falciferum*	*exaratum*	1639	44,443	belemnite	*Acrocoelites*	0,49	-4,81	OX	14,2	17	2,14	Ullmann et al. 2014[3]
Haws_CK_047	Toa	*falciferum*	*exaratum*	1657	44,470	belemnite	*Acrocoelites*	1,14	-4,26	OX	17,1	13	2,27	Ullmann et al. 2014[3]
Haws_CK_047	Toa	*falciferum*	*exaratum*	1657	44,470	belemnite	*Acrocoelites*	1,14	-3,69	OX	14,3	11	2,20	Ullmann et al. 2014[3]
Haws_CK_047	Toa	*falciferum*	*exaratum*	1657	44,470	belemnite	*Acrocoelites*	0,51	-3,43	OX	15,4	12	2,19	Ullmann et al. 2014[3]
Haws_CK_049	Toa	*falciferum*	*exaratum*	1695	44,526	belemnite	*Acrocoelites*	0,93	-5,76	OX	22,6	26	2,24	Ullmann et al. 2014[3]
Haws_CK_049	Toa	*falciferum*	*exaratum*	1695	44,526	belemnite	*Acrocoelites*	1,05	-5,67	OX	19,0	55	2,05	Ullmann et al. 2014[3]
Haws_CK_049	Toa	*falciferum*	*exaratum*	1695	44,526	belemnite	*Acrocoelites*	1,53	-4,95	OX	16,0	35	2,13	Ullmann et al. 2014[3]
Haws_CK_050	Toa	*falciferum*	*exaratum*	1720	44,563	belemnite	*Acrocoelites*	2,48	-4,10	OX	17,0	40	2,16	Ullmann et al. 2014[3]
Haws_CK_050	Toa	*falciferum*	*exaratum*	1720	44,563	belemnite	*Acrocoelites*	2,27	-3,95	OX	16,0	18	2,20	Ullmann et al. 2014[3]
Haws_CK_050	Toa	*falciferum*	*exaratum*	1720	44,563	belemnite	*Acrocoelites*	2,29	-3,25	OX	13,5	20	2,15	Ullmann et al. 2014[3]
Haws_CK_060	Toa	*falciferum*	*exaratum*	1774	44,642	belemnite	*Acrocoelites*	2,97	-4,59	OX	18,9	17	2,15	Ullmann et al. 2014[3]
Haws_CK_060	Toa	*falciferum*	*exaratum*	1774	44,642	belemnite	*Acrocoelites*	1,40	-5,09	OX	18,6	15	2,13	Ullmann et al. 2014[3]
Haws_CK_060	Toa	*falciferum*	*exaratum*	1774	44,642	belemnite	*Acrocoelites*	2,18	-4,05	OX	17,9	45	2,32	Ullmann et al. 2014[3]
Haws_CK_063	Toa	*falciferum*	*exaratum*	1845	44,747	belemnite	*Acrocoelites*	3,99	-2,36	OX	15,6	11	2,15	Ullmann et al. 2014[3]
Haws_CK_063	Toa	*falciferum*	*exaratum*	1845	44,747	belemnite	*Acrocoelites*	3,84	-3,25	OX	16,1	25	2,28	Ullmann et al. 2014[3]
Haws_CK_063	Toa	*falciferum*	*exaratum*	1845	44,747	belemnite	*Acrocoelites*	3,31	-2,48	OX	13,9	16	1,99	Ullmann et al. 2014[3]
Haws_CK_062	Toa	*falciferum*	*exaratum*	1845	44,747	belemnite	*Acrocoelites*	4,14	-4,00	OX	15,4	12	2,13	Ullmann et al. 2014[3]
Haws_CK_062	Toa	*falciferum*	*exaratum*	1845	44,747	belemnite	*Acrocoelites*	3,75	-2,22	OX	12,8	12	1,85	Ullmann et al. 2014[3]
Haws_CK_062	Toa	*falciferum*	*exaratum*	1845	44,747	belemnite	*Acrocoelites*	3,72	-2,75	OX	12,4	16	1,90	Ullmann et al. 2014[3]
Haws_CK_064	Toa	*falciferum*	*exaratum*	1845	44,747	belemnite	*Acrocoelites*	3,29	-2,92	OX	18,1	13	1,73	Ullmann et al. 2014[3]
Haws_CK_064	Toa	*falciferum*	*exaratum*	1845	44,747	belemnite	*Acrocoelites*	3,35	-2,87	OX	16,5	7	2,02	Ullmann et al. 2014[3]
Haws_CK_064	Toa	*falciferum*	*exaratum*	1845	44,747	belemnite	*Acrocoelites*	3,74	-3,29	OX	15,6	7	2,18	Ullmann et al. 2014[3]
Haws_CK_057	Toa	*falciferum*	*exaratum*	1847	44,750	belemnite	*Acrocoelites*	4,56	-3,42	OX	19,9	15	1,99	Ullmann et al. 2014[3]
Haws_CK_057	Toa	*falciferum*	*exaratum*	1847	44,750	belemnite	*Acrocoelites*	4,23	-4,50	OX	17,6	24	2,29	Ullmann et al. 2014[3]
Haws_CK_057	Toa	*falciferum*	*exaratum*	1847	44,750	belemnite	*Acrocoelites*	3,54	-4,97	OX	16,6	45	2,16	Ullmann et al. 2014[3]
Haws_CK_056	Toa	*falciferum*	*exaratum*	1858	44,766	belemnite	*Acrocoelites*	4,24	-3,07	OX	17,6	15	2,28	Ullmann et al. 2014[3]
Haws_CK_056	Toa	*falciferum*	*exaratum*	1858	44,766	belemnite	*Acrocoelites*	4,22	-3,48	OX	17,0	31	2,28	Ullmann et al. 2014[3]
Haws_CK_061	Toa	*falciferum*	*exaratum*	1911	44,844	belemnite	*Acrocoelites*	2,71	-6,34	OX	17,3	24	1,63	Ullmann et al. 2014[3]
Haws_CK_061	Toa	*falciferum*	*exaratum*	1911	44,844	belemnite	*Acrocoelites*	3,36	-5,11	OX	14,5	28	1,83	Ullmann et al. 2014[3]
Haws_CK_061	Toa	*falciferum*	*exaratum*	1911	44,844	belemnite	*Acrocoelites*	2,71	-5,50	OX	18,8	41	1,87	Ullmann et al. 2014[3]
Haws_CK_054	Toa	*falciferum*	*exaratum*	1920	44,857	belemnite	*Acrocoelites*	5,96	-4,19	OX	16,0	5	2,27	Ullmann et al. 2014[3]
Haws_CK_054	Toa	*falciferum*	*exaratum*	1920	44,857	belemnite	*Acrocoelites*	5,59	-4,47	OX	16,7	8	2,33	Ullmann et al. 2014[3]
Haws_CK_053	Toa	*falciferum*	*exaratum*	1954	44,907	belemnite	*Acrocoelites*	3,65	-2,30	OX	17,0	14	1,59	Ullmann et al. 2014[3]
Haws_CK_053	Toa	*falciferum*	*exaratum*	1954	44,907	belemnite	*Acrocoelites*	3,72	-2,13	OX	14,6	14	1,60	Ullmann et al. 2014[3]
Haws_CK_055	Toa	*falciferum*	*exaratum*	1985	44,953	belemnite	*Acrocoelites*	3,97	-1,28	OX	14,5	7	1,71	Ullmann et al. 2014[3]
Haws_CK_055	Toa	*falciferum*	*exaratum*	1985	44,953	belemnite	*Acrocoelites*	3,33	-1,84	OX	15,7	7	1,82	Ullmann et al. 2014[3]
base *falciferum* sz	Toa			2017										
base Bituminous Shales	Toa			2017										
HB-227	Toa	*falciferum*	*falciferum*	3405	45,622	belemnite	*Acrocoelites*	4,00	-4,01	OX	16,8	10	2,08	Ullmann et al. 2014[3]
HB-227	Toa	*falciferum*	*falciferum*	3405	45,622	belemnite	*Acrocoelites*	4,46	-3,91	OX	14,6	9	2,06	Ullmann et al. 2014[3]

HB-227	Toa	*falciferum*	*falciferum*	3405	45,622	belemnite	*Acrocoelites*	3,94	-4,92	OX	15,0	16	1,89	Ullmann et al. 2014[3]
base *bifrons* z	Toa			4250										
base *commune* sz	Toa			4250										
Top *ovatum* band	Toa			4250										

Saltwick Nab

Sal_N_4 a	Toa	*bifrons*	*commune*	2634	45,198	belemnite	*Acrocoelites*	3,10	-1,85	CPH	16,5	30	1,82	Ullmann et al. 2014[3]
Sal_N_4 b	Toa	*bifrons*	*commune*	2634	45,198	belemnite	*Acrocoelites*	1,86	-2,00	CPH	17,9	33	1,68	Ullmann et al. 2014[3]
Sal_N_4 c	Toa	*bifrons*	*commune*	2634	45,198	belemnite	*Acrocoelites*	2,37	-1,41	CPH	16,3	10	1,62	Ullmann et al. 2014[3]
Sal_N_8 a	Toa	*bifrons*	*commune*	2754	45,258	belemnite	*Acrocoelites*	1,22	-1,61	CPH	13,7	37	1,78	Ullmann et al. 2014[3]
Sal_N_8 b	Toa	*bifrons*	*commune*	2754	45,258	belemnite	*Acrocoelites*	0,87	-3,20	CPH	14,4	24	1,62	Ullmann et al. 2014[3]
Sal_N_8 c	Toa	*bifrons*	*commune*	2754	45,258	belemnite	*Acrocoelites*	1,73	-1,38	CPH	15,3	18	1,72	Ullmann et al. 2014[3]
Sal_N_12 a	Toa	*bifrons*	*commune*	2949	45,354	belemnite	*Youngibelus*	3,39	-3,01	CPH	12,1	12	1,66	Ullmann et al. 2014[3]
Sal_N_12 b	Toa	*bifrons*	*commune*	2949	45,354	belemnite	*Youngibelus*	1,56	-1,66	CPH	13,6	11	1,67	Ullmann et al. 2014[3]
Sal_N_12 c	Toa	*bifrons*	*commune*	2949	45,354	belemnite	*Youngibelus*	2,16	-1,40	CPH	16,1	14	2,01	Ullmann et al. 2014[3]
Sal_N_14 a	Toa	*bifrons*	*commune*	2764	45,263	belemnite	*Acrocoelites*	4,03	-3,34	CPH	10,9	5	1,97	Ullmann et al. 2014[3]
Sal_N_14 b	Toa	*bifrons*	*commune*	2764	45,263	belemnite	*Acrocoelites*	4,25	-3,55	CPH	11,2	4	1,95	Ullmann et al. 2014[3]
Sal_N_14 c	Toa	*bifrons*	*commune*	2764	45,263	belemnite	*Acrocoelites*	4,37	-3,06	CPH	12,4	5	2,11	Ullmann et al. 2014[3]
Sal_N_16 a	Toa	*bifrons*	*commune*	2999	45,379	belemnite	*Youngibelus*	2,37	-5,61	CPH	13,2	28	1,88	Ullmann et al. 2014[3]
Sal_N_16 b	Toa	*bifrons*	*commune*	2999	45,379	belemnite	*Youngibelus*	2,84	-2,81	CPH	16,4	8	1,81	Ullmann et al. 2014[3]
Sal_N_16 c	Toa	*bifrons*	*commune*	2999	45,379	belemnite	*Youngibelus*	2,07	-3,06	CPH	16,7	13	1,68	Ullmann et al. 2014[3]
Sal_N_17 a	Toa	*bifrons*	*commune*	2999	45,379	belemnite	*Acrocoelites* (?)	2,94	-3,14	CPH	16,1	14	1,67	Ullmann et al. 2014[3]
Sal_N_17 b	Toa	*bifrons*	*commune*	2999	45,379	belemnite	*Acrocoelites* (?)	1,25	-2,05	CPH	15,4	13	1,65	Ullmann et al. 2014[3]
Sal_N_17 c	Toa	*bifrons*	*commune*	2999	45,379	belemnite	*Acrocoelites* (?)	1,04	-1,78	CPH	18,2	20	1,88	Ullmann et al. 2014[3]
Sal_N_24 a	Toa	*bifrons*	*commune*	2949	45,354	belemnite	*Acrocoelites* (?)	2,15	-3,31	CPH	13,6	19	1,92	Ullmann et al. 2014[3]
Sal_N_24 b	Toa	*bifrons*	*commune*	2949	45,354	belemnite	*Acrocoelites* (?)	1,89	-1,47	CPH	14,1	12	2,04	Ullmann et al. 2014[3]
Sal_N_24 c	Toa	*bifrons*	*commune*	2949	45,354	belemnite	*Acrocoelites* (?)	1,51	-2,30	CPH	18,2	18	1,84	Ullmann et al. 2014[3]
Sal_N_26 a	Toa	*bifrons*	*commune*	3370	45,563	belemnite	*Acrocoelites* (?)	4,45	-4,35	CPH	14,1	8	2,01	Ullmann et al. 2014[3]
Sal_N_26 b	Toa	*bifrons*	*commune*	3370	45,563	belemnite	*Acrocoelites* (?)	4,54	-4,90	CPH	13,7	6	1,98	Ullmann et al. 2014[3]
Sal_N_26 c	Toa	*bifrons*	*commune*	3370	45,563	belemnite	*Acrocoelites* (?)	3,57	-5,32	CPH	13,3	8	1,78	Ullmann et al. 2014[3]
Sal_N_29 a	Toa	*bifrons*	*commune*	3405	45,581	belemnite	*Acrocoelites* (?)	4,62	-3,40	CPH	14,4	13	1,79	Ullmann et al. 2014[3]
Sal_N_29 b	Toa	*bifrons*	*commune*	3405	45,581	belemnite	*Acrocoelites* (?)	4,52	-4,43	CPH	14,9	6	1,86	Ullmann et al. 2014[3]
Sal_N_29 c	Toa	*bifrons*	*commune*	3405	45,581	belemnite	*Acrocoelites* (?)	3,88	-4,93	CPH	13,5	8	1,73	Ullmann et al. 2014[3]

Ravenscar

base *falciferum* sz	Toa			1873										
Peak Stones (midline)	Toa			2625										
Rave 3a	Toa	*falciferum*	*falciferum*	4110	45,941	belemnite	*Acrocoelites* (?)	3,53	-2,73	CPH		9	1,71	Ullmann et al. 2014[3]
Rave 3b	Toa	*falciferum*	*falciferum*	4110	45,941	belemnite	*Acrocoelites* (?)	3,98	-4,56	CPH		8	1,82	Ullmann et al. 2014[3]
Rave 1b	Toa	*falciferum*	*falciferum*	4140	45,954	belemnite	*Acrocoelites*	1,46	-1,54	CPH		9	1,78	Ullmann et al. 2014[3]
Rave 1a	Toa	*falciferum*	*falciferum*	4140	45,954	belemnite	*Acrocoelites*	2,27	-1,71	CPH		5	1,89	Ullmann et al. 2014[3]
Rave 2a	Toa	*falciferum*	*falciferum*	4140	45,954	belemnite	?	3,90	-3,80	CPH		18	1,93	Ullmann et al. 2014[3]
Rave 2b	Toa	*falciferum*	*falciferum*	4140	45,954	belemnite	?	4,27	-4,42	CPH		10	2,05	Ullmann et al. 2014[3]
Rave 4b	Toa	*falciferum*	*falciferum*	4143	45,955	belemnite	*Acrocoelites*	1,89	-1,05	CPH		16	1,62	Ullmann et al. 2014[3]
Rave 4a	Toa	*falciferum*	*falciferum*	4143	45,955	belemnite	*Acrocoelites*	2,47	-1,28	CPH		13	1,79	Ullmann et al. 2014[3]
Rave 6b	Toa	*falciferum*	*falciferum*	4210	45,983	belemnite	?	1,99	-1,42	CPH		19	1,66	Ullmann et al. 2014[3]
Rave 6a	Toa	*falciferum*	*falciferum*	4210	45,983	belemnite	?	3,66	-2,30	CPH		11	1,74	Ullmann et al. 2014[3]
base commune sz	Toa			4250										
base Hard Shales	Toa			4250										
top *ovatum* band	Toa			4250										
Rave 7b	Toa	*bifrons*	*commune*	4280	46,015	belemnite	*Acrocoelites* (?)	3,26	-2,48	CPH		13	1,80	Ullmann et al. 2014[3]
Rave 7a	Toa	*bifrons*	*commune*	4280	46,015	belemnite	*Acrocoelites* (?)	3,46	-2,48	CPH		11	1,91	Ullmann et al. 2014[3]
Rave 8a	Toa	*bifrons*	*commune*	4325	46,038	belemnite	*Acrocoelites*	3,80	-3,47	CPH		12	2,10	Ullmann et al. 2014[3]
Rave 8b	Toa	*bifrons*	*commune*	4325	46,038	belemnite	*Acrocoelites*	4,42	-3,86	CPH		16	2,18	Ullmann et al. 2014[3]
Rave 9b	Toa	*bifrons*	*commune*	4345	46,049	belemnite	*Acrocoelites* (?)	2,87	-2,10	CPH		11	1,98	Ullmann et al. 2014[3]
Rave 9a	Toa	*bifrons*	*commune*	4345	46,049	belemnite	*Acrocoelites* (?)	4,17	-3,28	CPH		8	2,01	Ullmann et al. 2014[3]
Rave 10b	Toa	*bifrons*	*commune*	4585	46,171	belemnite	*Acrocoelites* (?)	2,22	-0,98	CPH		22	1,71	Ullmann et al. 2014[3]
Rave 10a	Toa	*bifrons*	*commune*	4585	46,171	belemnite	*Acrocoelites* (?)	2,23	-1,04	CPH		14	1,61	Ullmann et al. 2014[3]
Rave 11b	Toa	*bifrons*	*commune*	4595	46,176	belemnite	*Acrocoelites*	1,71	-1,37	CPH		35	1,73	Ullmann et al. 2014[3]
Rave 11a	Toa	*bifrons*	*commune*	4595	46,176	belemnite	*Acrocoelites*	3,39	-3,02	CPH		10	1,91	Ullmann et al. 2014[3]
Rave 12a	Toa	*bifrons*	*commune*	4615	46,187	belemnite	*Acrocoelites* (?)	4,38	-3,04	CPH		6	2,16	Ullmann et al. 2014[3]
Rave 12b	Toa	*bifrons*	*commune*	4615	46,187	belemnite	*Acrocoelites* (?)	4,89	-3,41	CPH		7	2,13	Ullmann et al. 2014[3]
base Alum Shale	Toa	*bifrons*	*commune*	4640										
Rave 13b	Toa	*bifrons*	*commune*	4643	46,201	belemnite		1,92	-1,35	CPH		13	1,59	Ullmann et al. 2014[3]
Rave 13a	Toa	*bifrons*	*commune*	4643	46,201	belemnite		2,59	-3,35	CPH		23	1,80	Ullmann et al. 2014[3]
Rave 14	Toa	*bifrons*	*commune*	5295	46,535	belemnite		3,24	-3,29	CPH		26	1,90	this study (element data Ullmann et al. 2013[4])
Rave 14	Toa	*bifrons*	*commune*	5295	46,535	belemnite		4,07	-3,35	CPH		17	2,10	this study (element data Ullmann et al. 2013[4])
Rave 15	Toa	*bifrons*	*commune*	5330	46,552	belemnite		1,23	-1,50	CPH		21	1,72	this study (element data Ullmann et al. 2013[4])
Rave 15	Toa	*bifrons*	*commune*	5330	46,552	belemnite		2,18	-2,37	CPH		25	1,90	this study (element data Ullmann et al. 2013[4])
Rave 16	Toa	*bifrons*	*commune*	5335	46,555	belemnite		3,09	-3,27	CPH		18	2,06	this study (element data Ullmann et al. 2013[4])
Rave 16	Toa	*bifrons*	*commune*	5335	46,555	belemnite		3,18	-2,76	CPH		13	1,72	this study (element data Ullmann et al. 2013[4])
Rave 18	Toa	*bifrons*	*commune*	5475	46,627	belemnite		2,04	-1,69	CPH		19	1,77	this study (element data Ullmann et al. 2013[4])
Rave 18	Toa	*bifrons*	*commune*	5475	46,627	belemnite		2,90	-2,87	CPH		13	1,91	this study (element data Ullmann et al. 2013[4])
Rave 19	Toa	*bifrons*	*commune*	5515	46,647	belemnite		3,93	-3,32	CPH		8	2,12	this study (element data Ullmann et al. 2013[4])

Rave 19	Toa	*bifrons*	*commune*	5515	46,647	belemnite		4,31	-3,65	CPH	9	2,11	this study (element data Ullmann et al. 2013[4])
Rave 28	Toa	*bifrons*	*commune*	5565	46,673	belemnite		3,35	-3,33	CPH	7	2,06	this study (element data Ullmann et al. 2013[4])
Rave 28	Toa	*bifrons*	*commune*	5565	46,673	belemnite		3,38	-2,84	CPH	18	2,09	this study (element data Ullmann et al. 2013[4])
Rave 23	Toa	*bifrons*	*commune*	5605	46,693	belemnite		3,69	-1,70	CPH	10	2,13	this study (element data Ullmann et al. 2013[4])
Rave 23	Toa	*bifrons*	*commune*	5605	46,693	belemnite		3,79	-2,19	CPH	6	2,07	this study (element data Ullmann et al. 2013[4])
Rave 25	Toa	*bifrons*	*commune*	5670	46,726	belemnite		2,81	-3,27	CPH	9	2,09	this study (element data Ullmann et al. 2013[4])
Rave 25	Toa	*bifrons*	*commune*	5670	46,726	belemnite		3,70	-3,99	CPH	8	2,24	this study (element data Ullmann et al. 2013[4])
Rave 29	Toa	*bifrons*	*commune*	5730	46,757	belemnite		2,93	-3,35	CPH	12	2,01	this study (element data Ullmann et al. 2013[4])
Rave 29	Toa	*bifrons*	*commune*	5730	46,757	belemnite		3,47	-3,09	CPH	17	2,09	this study (element data Ullmann et al. 2013[4])
Rave 34	Toa	*bifrons*	*commune*	5730	46,757	belemnite		3,67	-3,05	CPH	8	1,99	this study (element data Ullmann et al. 2013[4])
Rave 34	Toa	*bifrons*	*commune*	5730	46,757	belemnite		4,30	-2,54	CPH	10	2,28	this study (element data Ullmann et al. 2013[4])
Rave 32	Toa	*bifrons*	*commune*	5765	46,775	belemnite		3,46	-3,04	CPH	10	1,84	this study (element data Ullmann et al. 2013[4])
Rave 32	Toa	*bifrons*	*commune*	5765	46,775	belemnite		3,85	-3,24	CPH	9	2,04	this study (element data Ullmann et al. 2013[4])
Rave 35	Toa	*bifrons*	*commune*	5925	46,857	belemnite		3,79	-3,08	CPH	7	1,94	this study (element data Ullmann et al. 2013[4])
Rave 35	Toa	*bifrons*	*commune*	5925	46,857	belemnite		4,02	-3,58	CPH	8	1,98	this study (element data Ullmann et al. 2013[4])
Rave 36	Toa	*bifrons*	*commune*	5925	46,857	belemnite		4,27	-2,55	CPH	11	1,81	this study (element data Ullmann et al. 2013[4])
Rave 36	Toa	*bifrons*	*commune*	5925	46,857	belemnite		4,59	-2,23	CPH	11	1,73	this study (element data Ullmann et al. 2013[4])
Rave 37	Toa	*bifrons*	*commune*	5975	46,882	belemnite		4,24	-3,68	CPH	9	2,10	this study (element data Ullmann et al. 2013[4])
Rave 37	Toa	*bifrons*	*commune*	5975	46,882	belemnite		4,39	-3,40	CPH	8	1,97	this study (element data Ullmann et al. 2013[4])
Rave 41	Toa	*bifrons*	*commune*	6020	46,905	belemnite		2,45	-1,46	CPH	13	1,73	this study (element data Ullmann et al. 2013[4])
Rave 38	Toa	*bifrons*	*commune*	6020	46,905	belemnite		2,63	-3,15	CPH	8	1,92	this study (element data Ullmann et al. 2013[4])
Rave 41	Toa	*bifrons*	*commune*	6020	46,905	belemnite		3,15	-0,91	CPH	7	1,78	this study (element data Ullmann et al. 2013[4])
Rave 38	Toa	*bifrons*	*commune*	6020	46,905	belemnite		3,17	-3,72	CPH	11	2,13	this study (element data Ullmann et al. 2013[4])
Rave 40	Toa	*bifrons*	*commune*	6020	46,905	belemnite		3,61	-3,15	CPH	17	1,92	this study (element data Ullmann et al. 2013[4])
Rave 40	Toa	*bifrons*	*commune*	6020	46,905	belemnite		4,20	-3,33	CPH	11	2,23	this study (element data Ullmann et al. 2013[4])
Rave 42	Toa	*bifrons*	*commune*	6045	46,918	belemnite		3,59	-3,14	CPH	13	1,76	this study (element data Ullmann et al. 2013[4])
Rave 42	Toa	*bifrons*	*commune*	6045	46,918	belemnite		3,72	-3,44	CPH	16	1,87	this study (element data Ullmann et al. 2013[4])
Rave 43	Toa	*bifrons*	*commune*	6070	46,931	belemnite		2,92	-2,63	CPH	19	1,79	this study (element data Ullmann et al. 2013[4])
Rave 43	Toa	*bifrons*	*commune*	6070	46,931	belemnite		2,95	-2,12	CPH	18	1,75	this study (element data Ullmann et al. 2013[4])
Rave 47	Toa	*bifrons*	*commune*	6125	46,959	belemnite		2,80	-2,58	CPH	11	2,24	this study (element data Ullmann et al. 2013[4])
Rave 47	Toa	*bifrons*	*commune*	6125	46,959	belemnite		4,10	-1,99	CPH	17	2,06	this study (element data Ullmann et al. 2013[4])
base *fibulatum* sz	Toa			6205									
Rave 44	Toa	*bifrons*	*fibulatum*	6235	47,029	belemnite		3,21	-3,35	CPH	14	1,96	this study (element data Ullmann et al. 2013[4])
Rave 44	Toa	*bifrons*	*fibulatum*	6235	47,029	belemnite		3,52	-2,76	CPH	10	1,90	this study (element data Ullmann et al. 2013[4])
Rave 51	Toa	*bifrons*	*fibulatum*	6375	47,163	belemnite		3,68	-3,19	CPH	12	1,90	this study (element data Ullmann et al. 2013[4])
Rave 51	Toa	*bifrons*	*fibulatum*	6375	47,163	belemnite		4,27	-3,22	CPH	10	2,32	this study (element data Ullmann et al. 2013[4])
Rave 49	Toa	*bifrons*	*fibulatum*	6425	47,211	belemnite		3,04	-4,47	CPH	24	1,35	this study (element data Ullmann et al. 2013[4])
Rave 49	Toa	*bifrons*	*fibulatum*	6425	47,211	belemnite		3,29	-2,43	CPH	16	1,81	this study (element data Ullmann et al. 2013[4])
Rave 48	Toa	*bifrons*	*fibulatum*	6510	47,292	belemnite	altered	-4,36	-12,51	CPH	118	0,66	this study
Rave 48	Toa	*bifrons*	*fibulatum*	6510	47,292	belemnite	altered	1,28	-6,44	CPH	51	1,40	this study (element data Ullmann et al. 2013[4])
Rave 52	Toa	*bifrons*	*fibulatum*	6535	47,316	belemnite		3,90	-3,43	CPH	8	2,33	this study (element data Ullmann et al. 2013[4])
Rave 52	Toa	*bifrons*	*fibulatum*	6535	47,316	belemnite		4,46	-2,52	CPH	8	2,51	this study (element data Ullmann et al. 2013[4])
Rave 54	Toa	*bifrons*	*fibulatum*	6565	47,344	belemnite		3,72	-3,77	CPH	8	2,10	this study (element data Ullmann et al. 2013[4])
Rave 54	Toa	*bifrons*	*fibulatum*	6565	47,344	belemnite		4,69	-2,96	CPH	11	2,39	this study (element data Ullmann et al. 2013[4])
Rave 56	Toa	*bifrons*	*fibulatum*	6700	47,474	belemnite		3,65	-3,31	CPH	9	1,96	this study (element data Ullmann et al. 2013[4])
Rave 56	Toa	*bifrons*	*fibulatum*	6700	47,474	belemnite		3,72	-4,16	CPH	10	1,91	this study (element data Ullmann et al. 2013[4])
base Cement Shales	Toa	*bifrons*	*fibulatum*	6740									
Rave 57	Toa	*bifrons*	*fibulatum*	6810	47,579	belemnite		3,08	-3,26	CPH	12	1,96	this study (element data Ullmann et al. 2013[4])
Rave 57	Toa	*bifrons*	*fibulatum*	6810	47,579	belemnite		3,40	-2,67	CPH	12	1,96	this study (element data Ullmann et al. 2013[4])
Rave 59	Toa	*bifrons*	*fibulatum*	6815	47,584	belemnite		3,76	-4,68	CPH	11	1,86	this study (element data Ullmann et al. 2013[4])
Rave 59	Toa	*bifrons*	*fibulatum*	6815	47,584	belemnite		3,85	-3,80	CPH	4	2,03	this study (element data Ullmann et al. 2013[4])
Rave 58	Toa	*bifrons*	*fibulatum*	6885	47,651	belemnite		4,02	-3,76	CPH	10	1,97	this study (element data Ullmann et al. 2013[4])
Rave 58	Toa	*bifrons*	*fibulatum*	6885	47,651	belemnite		4,38	-3,08	CPH	6	2,03	this study (element data Ullmann et al. 2013[4])
Dean's bed 21 centre line	Toa	*bifrons*	*fibulatum*	6985									
Rave 60	Toa	*bifrons*	*fibulatum*	7040	47,799	belemnite		2,83	-2,74	CPH	15	1,64	this study (element data Ullmann et al. 2013[4])
Rave 60	Toa	*bifrons*	*fibulatum*	7040	47,799	belemnite		3,50	-2,11	CPH	0	1,63	this study (element data Ullmann et al. 2013[4])
Rave 61	Toa	*bifrons*	*fibulatum*	7160	47,914	belemnite		4,08	-2,65	CPH	7	1,89	this study (element data Ullmann et al. 2013[4])
Rave 61	Toa	*bifrons*	*fibulatum*	7160	47,914	belemnite		4,16	-2,38	CPH	6	1,97	this study (element data Ullmann et al. 2013[4])
base *crassum* sz	Toa			7250									
Rave 62	Toa	*bifrons*	*crassum*	7315	48,067	belemnite		1,55	-2,75	CPH	10	2,13	this study (element data Ullmann et al. 2013[4])
Rave 63	Toa	*bifrons*	*crassum*	7315	48,067	belemnite		1,99	-2,97	CPH	20	2,10	this study (element data Ullmann et al. 2013[4])
Rave 63	Toa	*bifrons*	*crassum*	7315	48,067	belemnite		2,12	-2,56	CPH	19	2,05	this study (element data Ullmann et al. 2013[4])
Rave 62	Toa	*bifrons*	*crassum*	7315	48,067	belemnite		2,85	-3,08	CPH	13	2,13	this study (element data Ullmann et al. 2013[4])
Rave 64	Toa	*bifrons*	*crassum*	7425	48,181	belemnite		3,60	-2,36	CPH	9	1,97	this study (element data Ullmann et al. 2013[4])
Rave 64	Toa	*bifrons*	*crassum*	7425	48,181	belemnite		4,39	-2,29	CPH	46	1,84	this study (element data Ullmann et al. 2013[4])
Rave 65	Toa	*bifrons*	*crassum*	7500	48,259	belemnite		4,02	-2,74	CPH	10	2,00	this study (element data Ullmann et al. 2013[4])
Rave 65	Toa	*bifrons*	*crassum*	7500	48,259	belemnite		4,21	-2,74	CPH	10	2,19	this study (element data Ullmann et al. 2013[4])
Rave 66	Toa	*bifrons*	*crassum*	7595	48,358	belemnite		3,02	-3,22	CPH	10	1,73	this study (element data Ullmann et al. 2013[4])
Rave 66	Toa	*bifrons*	*crassum*	7595	48,358	belemnite		3,75	-3,54	CPH	16	2,00	this study (element data Ullmann et al. 2013[4])
Rave 68	Toa	*bifrons*	*crassum*	7665	48,430	belemnite		3,68	-3,79	CPH	5	2,27	this study (element data Ullmann et al. 2013[4])
Rave 68	Toa	*bifrons*	*crassum*	7665	48,430	belemnite		3,86	-3,90	CPH	7	2,09	this study (element data Ullmann et al. 2013[4])
Rave 73	Toa	*bifrons*	*crassum*	7710	48,477	belemnite	altered	1,13	-5,08	CPH	104	1,73	this study (element data Ullmann et al. 2013[4])
Rave 73	Toa	*bifrons*	*crassum*	7710	48,477	belemnite	altered	1,89	-4,68	CPH	61	2,03	this study (element data Ullmann et al. 2013[4])
Rave 69	Toa	*bifrons*	*crassum*	7740	48,508	belemnite		3,17	-3,11	CPH	28	1,83	this study (element data Ullmann et al. 2013[4])
Rave 69	Toa	*bifrons*	*crassum*	7740	48,508	belemnite		3,40	-2,23	CPH	18	1,73	this study (element data Ullmann et al. 2013[4])
Rave 72	Toa	*bifrons*	*crassum*	7790	48,560	belemnite		1,99	-1,08	CPH	14	1,96	this study (element data Ullmann et al. 2013[4])
Rave 72	Toa	*bifrons*	*crassum*	7790	48,560	belemnite		2,31	-2,53	CPH	10	1,85	this study (element data Ullmann et al. 2013[4])
Rave 71	Toa	*bifrons*	*crassum*	7890	48,663	belemnite		2,00	-1,44	CPH	25	1,65	this study (element data Ullmann et al. 2013[4])
Rave 71	Toa	*bifrons*	*crassum*	7890	48,663	belemnite		3,46	-2,82	CPH	11	1,71	this study (element data Ullmann et al. 2013[4])

base *variabilis* z	Toa			8215									
Rave 84	Toa	*variabilis*	-	8365	49,097	belemnite		2,28	-4,03	CPH	20	2,05	this study (element data Ullmann et al. 2013[4])
Rave 84	Toa	*variabilis*	-	8365	49,097	belemnite		3,35	-2,73	CPH	8	2,13	this study (element data Ullmann et al. 2013[4])
Rave 81	Toa	*variabilis*	-	8420	49,133	belemnite		3,68	-2,93	CPH	6	2,15	this study (element data Ullmann et al. 2013[4])
Rave 81	Toa	*variabilis*	-	8420	49,133	belemnite		3,70	-3,56	CPH	7	2,04	this study (element data Ullmann et al. 2013[4])
Rave 82	Toa	*variabilis*	-	8425	49,136	belemnite	altered	1,25	-2,84	CPH	399	1,18	this study
Rave 82	Toa	*variabilis*	-	8425	49,136	belemnite	altered	-1,73	-2,89	CPH	862	1,22	this study
Rave 80	Toa	*variabilis*	-	8445	49,149	belemnite		3,58	-3,06	CPH	9	1,94	this study (element data Ullmann et al. 2013[4])
Rave 80	Toa	*variabilis*	-	8445	49,149	belemnite		4,08	-2,89	CPH	14	2,05	this study (element data Ullmann et al. 2013[4])
Rave 74	Toa	*variabilis*	-	8465	49,162	belemnite		2,99	-4,31	CPH	8	2,15	this study (element data Ullmann et al. 2013[4])
Rave 74	Toa	*variabilis*	-	8465	49,162	belemnite		3,44	-4,15	CPH	21	2,14	this study (element data Ullmann et al. 2013[4])
Rave 78	Toa	*variabilis*	-	8495	49,182	belemnite		2,19	-3,61	CPH	16	1,87	this study (element data Ullmann et al. 2013[4])
Rave 79	Toa	*variabilis*	-	8495	49,182	belemnite		2,45	-3,25	CPH	12	1,95	this study (element data Ullmann et al. 2013[4])
Rave 78	Toa	*variabilis*	-	8495	49,182	belemnite		2,85	-3,78	CPH	15	2,05	this study (element data Ullmann et al. 2013[4])
Rave 79	Toa	*variabilis*	-	8495	49,182	belemnite		3,40	-3,25	CPH	11	2,35	this study (element data Ullmann et al. 2013[4])
Rave 86	Toa	*variabilis*	-	8525	49,201	belemnite		3,19	-3,04	CPH	24	1,95	this study (element data Ullmann et al. 2013[4])
Rave 86	Toa	*variabilis*	-	8525	49,201	belemnite		4,76	-2,53	CPH	11	2,12	this study (element data Ullmann et al. 2013[4])
Rave 76	Toa	*variabilis*	-	8610	49,256	belemnite	altered	0,87	-5,53	CPH	68	1,46	this study (element data Ullmann et al. 2013[4])
Rave 76	Toa	*variabilis*	-	8610	49,256	belemnite		2,68	-2,94	CPH	7	1,78	this study (element data Ullmann et al. 2013[4])
Rave 77	Toa	*variabilis*	-	8620	49,263	belemnite		3,06	-2,22	CPH	10	1,81	this study (element data Ullmann et al. 2013[4])
Rave 77	Toa	*variabilis*	-	8620	49,263	belemnite		3,61	-2,40	CPH	9	1,98	this study (element data Ullmann et al. 2013[4])
Rave 89	Toa	*variabilis*	-	8630	49,269	belemnite	altered	-0,38	-10,78	CPH	85	0,66	this study
Rave 89	Toa	*variabilis*	-	8630	49,269	belemnite		3,06	-1,69	CPH	14	2,28	this study (element data Ullmann et al. 2013[4])
Rave 94	Toa	*variabilis*	-	8685	49,305	belemnite		2,62	-3,04	CPH	11	1,98	this study (element data Ullmann et al. 2013[4])
Rave 94	Toa	*variabilis*	-	8685	49,305	belemnite		4,13	-3,63	CPH	9	2,20	this study (element data Ullmann et al. 2013[4])
Rave 93	Toa	*variabilis*	-	8695	49,312	belemnite		3,11	-2,98	CPH	24	1,86	this study (element data Ullmann et al. 2013[4])
Rave 93	Toa	*variabilis*	-	8695	49,312	belemnite		3,51	-2,98	CPH	9	2,24	this study (element data Ullmann et al. 2013[4])
Rave 92	Toa	*variabilis*	-	8695	49,312	belemnite	altered	4,74	-3,56	CPH	54	0,85	this study
Rave 92	Toa	*variabilis*	-	8695	49,312	belemnite	altered	4,67	-3,38	CPH	135	0,80	this study
TV_002	Toa	*variabilis*	-	8720	49,328	belemnite		4,48	-3,88	CPH	3	2,11	this study
TV_002	Toa	*variabilis*	-	8720	49,328	belemnite		4,28	-4,02	CPH	3	2,14	this study
TV_002	Toa	*variabilis*	-	8720	49,328	belemnite		3,67	-4,35	CPH	13	2,09	this study
Rave 95	Toa	*variabilis*	-	8745	49,344	belemnite		2,92	-2,80	CPH	12	1,94	this study (element data Ullmann et al. 2013[4])
Rave 95	Toa	*variabilis*	-	8745	49,344	belemnite		3,47	-2,80	CPH	11	1,77	this study (element data Ullmann et al. 2013[4])
TV_004	Toa	*variabilis*	-	8815	49,390	belemnite		1,51	-1,33	CPH	19	1,67	this study
TV_004	Toa	*variabilis*	-	8815	49,390	belemnite		1,95	-2,35	CPH	15	1,84	this study
TV_004	Toa	*variabilis*	-	8815	49,390	belemnite		2,39	-3,61	CPH	14	1,84	this study
TV_004b	Toa	*variabilis*	-	8815	49,390	belemnite		3,29	-3,40	CPH	12	1,58	this study
TV_004b	Toa	*variabilis*	-	8815	49,390	belemnite		2,78	-2,99	CPH	7	1,52	this study
TV_004b	Toa	*variabilis*	-	8815	49,390	belemnite		2,53	-3,03	CPH	9	1,62	this study
base Peak Mudstone	Toa	*variabilis*	-	8835									
Rave 99	Toa	*variabilis*	-	8975	49,494	belemnite		2,14	-2,91	CPH	7	1,72	this study (element data Ullmann et al. 2013[4])
Rave 99	Toa	*variabilis*	-	8975	49,494	belemnite		3,22	-3,08	CPH	11	1,85	this study (element data Ullmann et al. 2013[4])
TV_009	Toa	*variabilis*	-	8995	49,506	belemnite		3,82	-3,32	CPH	9	1,86	this study
TV_009	Toa	*variabilis*	-	8995	49,506	belemnite		3,76	-2,44	CPH	6	1,84	this study
TV_009	Toa	*variabilis*	-	8995	49,506	belemnite		3,02	-2,81	CPH	8	1,69	this study
Rave 114	Toa	*variabilis*	-	9005	49,513	belemnite		3,14	-2,50	CPH	15	1,82	this study (element data Ullmann et al. 2013[4])
Rave 114	Toa	*variabilis*	-	9005	49,513	belemnite		3,21	-2,19	CPH	17	1,74	this study (element data Ullmann et al. 2013[4])
Rave 114	Toa	*variabilis*	-	9005	49,513	belemnite	altered	4,91	-3,27	CPH	60	0,77	this study
Rave 114	Toa	*variabilis*	-	9005	49,513	belemnite	altered	4,69	-3,40	CPH	69	0,74	this study
Rave 101	Toa	*variabilis*	-	9055	49,545	belemnite		2,54	-1,92	CPH	10	2,06	this study (element data Ullmann et al. 2013[4])
Rave 101	Toa	*variabilis*	-	9055	49,545	belemnite		2,91	-2,09	CPH	15	1,79	this study (element data Ullmann et al. 2013[4])
Rave 100	Toa	*variabilis*	-	9075	49,558	belemnite		2,63	-2,85	CPH	9	1,65	this study (element data Ullmann et al. 2013[4])
Rave 100	Toa	*variabilis*	-	9075	49,558	belemnite		3,39	-2,48	CPH	6	1,78	this study (element data Ullmann et al. 2013[4])
TV_011	Toa	*variabilis*	-	9080	49,562	belemnite		1,42	-3,37	CPH	11	2,00	this study
TV_011	Toa	*variabilis*	-	9080	49,562	belemnite		1,47	-2,26	CPH	20	1,80	this study
TV_011	Toa	*variabilis*	-	9080	49,562	belemnite		0,93	-1,15	CPH	16	1,56	this study
TV_012	Toa	*variabilis*	-	9135	49,597	belemnite		2,40	-4,17	CPH	22	2,26	this study
TV_012	Toa	*variabilis*	-	9135	49,597	belemnite		2,39	-3,46	CPH	6	2,17	this study
TV_012	Toa	*variabilis*	-	9135	49,597	belemnite		2,54	-3,45	CPH	10	2,07	this study
TV_012b	Toa	*variabilis*	-	9135	49,597	belemnite		2,70	-3,67	CPH	11	2,64	this study
TV_012b	Toa	*variabilis*	-	9135	49,597	belemnite		1,98	-3,46	CPH	33	2,10	this study
TV_012b	Toa	*variabilis*	-	9135	49,597	belemnite		2,35	-2,92	CPH	11	2,27	this study
TV_015	Toa	*variabilis*	-	9190	49,633	belemnite		3,55	-2,51	CPH	10	1,87	this study
TV_015	Toa	*variabilis*	-	9190	49,633	belemnite		3,25	-2,43	CPH	16	1,85	this study
TV_015	Toa	*variabilis*	-	9190	49,633	belemnite		3,45	-2,66	CPH	9	1,77	this study
Rave 103	Toa	*variabilis*	-	9195	49,636	belemnite		2,89	-2,74	CPH	13	1,86	this study (element data Ullmann et al. 2013[4])
Rave 103	Toa	*variabilis*	-	9195	49,636	belemnite		3,08	-2,68	CPH	14	1,66	this study (element data Ullmann et al. 2013[4])
Rave 104	Toa	*variabilis*	-	9205	49,643	belemnite		3,15	-3,35	CPH	8	2,20	this study (element data Ullmann et al. 2013[4])
Rave 104	Toa	*variabilis*	-	9205	49,643	belemnite		3,34	-3,25	CPH	12	2,05	this study (element data Ullmann et al. 2013[4])
Rave 106	Toa	*variabilis*	-	9255	49,675	belemnite	altered	2,35	-4,45	CPH			this study
Rave 106	Toa	*variabilis*	-	9255	49,675	belemnite		2,64	-3,71	CPH	36	2,12	this study (element data Ullmann et al. 2013[4])
Rave 105	Toa	*variabilis*	-	9265	49,682	belemnite		2,57	-1,51	CPH	17	1,99	this study (element data Ullmann et al. 2013[4])
Rave 105	Toa	*variabilis*	-	9265	49,682	belemnite		2,60	-1,83	CPH	8	1,73	this study (element data Ullmann et al. 2013[4])
Rave 108	Toa	*variabilis*	-	9295	49,701	belemnite		2,74	-3,59	CPH	37	2,36	this study (element data Ullmann et al. 2013[4])
Rave 108	Toa	*variabilis*	-	9295	49,701	belemnite		3,68	-3,27	CPH	16	2,40	this study (element data Ullmann et al. 2013[4])
Rave 111	Toa	*variabilis*	-	9345	49,734	belemnite		2,57	-1,93	CPH	26	1,82	this study (element data Ullmann et al. 2013[4])
Rave 111	Toa	*variabilis*	-	9345	49,734	belemnite		2,88	-2,93	CPH	32	2,07	this study (element data Ullmann et al. 2013[4])
Rave 109	Toa	*variabilis*	-	9350	49,737	belemnite		3,43	-3,47	CPH	10	2,10	this study (element data Ullmann et al. 2013[4])

Rave 109	Toa	*variabilis*	-	9350	49,737	belemnite		3,88	-3,65	CPH		11	2,06	this study (element data Ullmann et al. 2013[4])
TV_018 a	Toa	*variabilis*	-	9375	49,753	belemnite	no element data	1,40	-2,95	CPH				this study
TV_018 b	Toa	*variabilis*	-	9375	49,753	belemnite	no element data	2,36	-2,84	CPH				this study
TV_018 c	Toa	*variabilis*	-	9375	49,753	belemnite	no element data	2,36	-3,04	CPH				this study
Rave 112	Toa	*variabilis*	-	9415	49,779	belemnite		2,35	-2,55	CPH		17	2,02	this study (element data Ullmann et al. 2013[4])
Rave 112	Toa	*variabilis*	-	9415	49,779	belemnite		2,78	-3,07	CPH		20	1,65	this study (element data Ullmann et al. 2013[4])
Rave 115	Toa	*variabilis*	-	9595	49,896	belemnite		2,40	-4,48	CPH		97	2,44	this study (element data Ullmann et al. 2013[4])
Rave 115	Toa	*variabilis*	-	9595	49,896	belemnite		2,85	-3,20	CPH		21	2,17	this study (element data Ullmann et al. 2013[4])
base *thouarsense* z	Toa			9755										
base *striatulum* sz	Toa			9755										
base Fox Cliff Siltstone	Toa			10185										
base Bed 57	Toa			10185										
base *fallaciosum* sz	Toa			10185										
Rav_ALE_26	Toa	*thouarsense*	*fallaciosum*	10195	51,012	belemnite		1,53	-2,57	CPH	7,8	294	1,86	this study
Rav_ALE_26	Toa	*thouarsense*	*fallaciosum*	10195	51,012	belemnite		2,61	-1,75	CPH	8,1	13	1,94	this study
Rav_ALE_26	Toa	*thouarsense*	*fallaciosum*	10195	51,012	belemnite		3,13	-1,74	CPH	8,1	12	1,82	this study
base Bed 58	Toa	*thouarsense*	*fallaciosum*	10200	51,019									
base Bed 59	Toa	*thouarsense*	*fallaciosum*	10540	51,438									
base Bed 60	Toa	*thouarsense*	*fallaciosum*	10555	51,457									
base Bed 61	Toa	*thouarsense*	*fallaciosum*	10685	51,617									
base Bed 62	Toa	*thouarsense*	*fallaciosum*	10715	51,654									
Rav_ALE_28	Toa	*thouarsense*	*fallaciosum*	10800	51,759	belemnite		3,18	-3,96	CPH		5	2,01	this study
Rav_ALE_28	Toa	*thouarsense*	*fallaciosum*	10800	51,759	belemnite		3,00	-3,47	CPH		98	2,02	this study
Rav_ALE_28	Toa	*thouarsense*	*fallaciosum*	10800	51,759	belemnite		2,28	-3,39	CPH		14	2,00	this study
Rav_CK_69	Toa	*thouarsense*	*fallaciosum*	10815	51,778	belemnite		1,45	-2,55	CPH		35	1,90	this study
Rav_CK_69	Toa	*thouarsense*	*fallaciosum*	10815	51,778	belemnite		2,36	-1,39	CPH		6	1,76	this study
Rav_CK_69	Toa	*thouarsense*	*fallaciosum*	10815	51,778	belemnite		1,83	-3,93	CPH		26	1,90	this study
Rav_CK_67	Toa	*thouarsense*	*fallaciosum*	10915	51,901	belemnite		3,44	-2,58	CPH		3	2,15	this study
Rav_CK_67	Toa	*thouarsense*	*fallaciosum*	10915	51,901	belemnite		2,85	-3,11	CPH		2	2,21	this study
Rav_CK_67	Toa	*thouarsense*	*fallaciosum*	10915	51,901	belemnite		3,09	-3,34	CPH		4	2,19	this study
Rav_CK_60	Toa	*thouarsense*	*fallaciosum*	10925	51,914	belemnite		2,02	-3,51	CPH	9,8	8	1,72	this study
Rav_CK_60	Toa	*thouarsense*	*fallaciosum*	10925	51,914	belemnite		2,52	-3,51	CPH	9,7	26	1,76	this study
Rav_CK_60	Toa	*thouarsense*	*fallaciosum*	10925	51,914	belemnite		1,51	-3,69	CPH	11,0	20	1,95	this study
Rav_ALE_29	Toa	*thouarsense*	*fallaciosum*	10950	51,944	belemnite		3,61	-3,11	CPH		2	2,10	this study
Rav_ALE_29	Toa	*thouarsense*	*fallaciosum*	10950	51,944	belemnite		3,84	-3,29	CPH		2	2,10	this study
Rav_ALE_29	Toa	*thouarsense*	*fallaciosum*	10950	51,944	belemnite		3,49	-3,20	CPH		8	2,18	this study
Rav_CK_71	Toa	*thouarsense*	*fallaciosum*	10990	51,994	belemnite		2,34	-4,13	CPH		29	1,94	this study
Rav_CK_71	Toa	*thouarsense*	*fallaciosum*	10990	51,994	belemnite		2,45	-3,95	CPH		68	2,01	this study
Rav_CK_71	Toa	*thouarsense*	*fallaciosum*	10990	51,994	belemnite		2,46	-4,01	CPH		39	2,16	this study
base Bed 64	Toa			10995										
base *levesquei* z	Toa			10995										
base *dispansum* sz	Toa			10995										
Rav_CK_73	Toa	*levesquei*	*dispansum*	11015	52,017	belemnite		1,65	-2,72	CPH	9,0	7	1,69	this study
Rav_CK_73	Toa	*levesquei*	*dispansum*	11015	52,017	belemnite		1,21	-3,01	CPH	8,6	12	1,68	this study
Rav_CK_73	Toa	*levesquei*	*dispansum*	11015	52,017	belemnite		1,55	-3,34	CPH	9,5	12	1,77	this study
base Bed 65	Toa	*levesquei*	*dispansum*	11110	52,097									
base Bed 66	Toa	*levesquei*	*dispansum*	11125	52,109									
Rav_CK_065	Toa	*levesquei*	*dispansum*	11145	52,126	belemnite		1,71	-5,24	CPH	15,1	66	1,84	this study
Rav_CK_065	Toa	*levesquei*	*dispansum*	11145	52,126	belemnite		2,08	-5,16	CPH	13,6	48	1,83	this study
Rav_CK_065	Toa	*levesquei*	*dispansum*	11145	52,126	belemnite		2,97	-3,71	CPH	10,5	14	1,79	this study
Rav_CK_76	Toa	*levesquei*	*dispansum*	11155	52,134	belemnite		0,11	-1,66	CPH	13,4	11	1,48	this study
Rav_CK_76	Toa	*levesquei*	*dispansum*	11155	52,134	belemnite		0,25	-1,82	CPH	12,1	16	1,55	this study
Rav_CK_76	Toa	*levesquei*	*dispansum*	11155	52,134	belemnite		-0,10	-1,60	CPH	11,8	15	1,50	this study
base Bed 67	Toa	*levesquei*	*dispansum*	11165	52,143									
base Bed 68	Toa	*levesquei*	*dispansum*	11185	52,160									
base Bed 69	Toa	*levesquei*	*dispansum*	11230	52,197									
base Bed 70	Toa	*levesquei*	*dispansum*	11245	52,210									
base Bed 71	Toa	*levesquei*	*dispansum*	11265	52,227									
base Grey Sandstone	Toa	*levesquei*	*dispansum*	11265	52,227									
base Bed 73	Toa	*levesquei*	*dispansum*	11340	52,290									
base Bed 74	Toa	*levesquei*	*dispansum*	11380	52,324									
Rav_MR_14	Toa	*levesquei*	*dispansum*	11525	52,445	belemnite		3,88	-2,70	CPH		28	2,04	this study
Rav_MR_14	Toa	*levesquei*	*dispansum*	11525	52,445	belemnite		2,83	-2,55	CPH		17	2,03	this study
Rav_MR_14	Toa	*levesquei*	*dispansum*	11525	52,445	belemnite		2,82	-2,25	CPH		24	1,98	this study
base Bed 75	Toa	*levesquei*	*dispansum*	11565	52,479									
Rav_NT_5	Toa	*levesquei*	*dispansum*	11570	52,483	belemnite		2,23	-2,56	CPH		57	2,05	this study
Rav_NT_5	Toa	*levesquei*	*dispansum*	11570	52,483	belemnite		1,97	-2,80	CPH		24	2,01	this study
Rav_NT_5	Toa	*levesquei*	*dispansum*	11570	52,483	belemnite		2,40	-2,72	CPH		19	1,93	this study
Rav_CVU_5	Toa	*levesquei*	*dispansum*	11575	52,487	belemnite		3,16	-3,63	CPH		3	2,00	this study
Rav_CVU_5	Toa	*levesquei*	*dispansum*	11575	52,487	belemnite		3,07	-3,64	CPH		4	1,95	this study
Rav_CVU_5	Toa	*levesquei*	*dispansum*	11575	52,487	belemnite		2,70	-3,44	CPH		7	1,87	this study
Rav_NT_4	Toa	*levesquei*	*dispansum*	11575	52,487	belemnite		3,02	-2,99	CPH	9,5	5	1,89	this study
Rav_NT_4	Toa	*levesquei*	*dispansum*	11575	52,487	belemnite		2,23	-3,44	CPH	8,5	4	1,71	this study
Rav_NT_4	Toa	*levesquei*	*dispansum*	11575	52,487	belemnite		2,92	-3,29	CPH	9,3	11	1,79	this study
base Bed 76	Toa	*levesquei*	*dispansum*	11575	52,487									
Rav_MEJ_4	Toa	*levesquei*	*dispansum*	11615	52,521	belemnite		2,72	-2,70	CPH		20	1,98	this study
Rav_MEJ_4	Toa	*levesquei*	*dispansum*	11615	52,521	belemnite		2,74	-2,83	CPH		20	1,68	this study
Rav_MEJ_4	Toa	*levesquei*	*dispansum*	11615	52,521	belemnite		2,33	-3,10	CPH		26	1,74	this study
Rav_MEJ_6	Toa	*levesquei*	*dispansum*	11625	52,529	belemnite		2,93	-2,77	CPH		21	1,70	this study

Rav_MEJ_7		Toa	*levesquei*	*dispansum*	11625	52,529	belemnite		1,19	-3,45	CPH		14	1,70	this study
Rav_MEJ_7		Toa	*levesquei*	*dispansum*	11625	52,529	belemnite		4,02	-2,85	CPH		13	1,76	this study
Rav_NT_1		Toa	*levesquei*	*dispansum*	11650	52,550	belemnite		0,50	-2,05	CPH		14	1,65	this study
Rav_NT_1		Toa	*levesquei*	*dispansum*	11650	52,550	belemnite		1,51	-3,25	CPH		16	1,65	this study
Rav_NT_1		Toa	*levesquei*	*dispansum*	11650	52,550	belemnite		1,55	-3,91	CPH		23	1,67	this study
Rav_NT_2		Toa	*levesquei*	*dispansum*	11650	52,550	belemnite		2,63	-3,33	CPH		31	1,62	this study
Rav_NT_2		Toa	*levesquei*	*dispansum*	11650	52,550	belemnite		2,77	-2,89	CPH		13	1,68	this study
Rav_NT_2		Toa	*levesquei*	*dispansum*	11650	52,550	belemnite		3,59	-3,19	CPH		31	1,67	this study
Rav_CK_14		Toa	*levesquei*	*dispansum*	11680	52,576	belemnite		3,38	-3,95	CPH		12	2,01	this study
Rav_CK_14		Toa	*levesquei*	*dispansum*	11680	52,576	belemnite		2,98	-3,69	CPH		52	1,93	this study
Rav_CK_14		Toa	*levesquei*	*dispansum*	11680	52,576	belemnite		2,86	-3,79	CPH		74	1,93	this study
	base Bed 77	Toa	*levesquei*	*dispansum*	11680	52,576									
	base Bed 78	Toa	*levesquei*	*dispansum*	11690	52,584									
Rav_CK_16		Toa	*levesquei*	*dispansum*	11730	52,618	belemnite		3,53	-3,50	CPH		3	1,95	this study
Rav_CK_16		Toa	*levesquei*	*dispansum*	11730	52,618	belemnite		3,53	-3,53	CPH		6	1,99	this study
	base Bed 79	Toa	*levesquei*	*dispansum*	11800	52,676									
	base Bed 80	Toa	*levesquei*	*dispansum*	11815	52,689									
Rav_MR_2		Toa	*levesquei*	*dispansum*	11820	52,693	belemnite		3,48	-3,34	CPH		61	2,14	this study
Rav_MR_2		Toa	*levesquei*	*dispansum*	11820	52,693	belemnite		3,47	-3,64	CPH		16	2,04	this study
Rav_MR_2		Toa	*levesquei*	*dispansum*	11820	52,693	belemnite		3,39	-3,33	CPH		10	2,01	this study
Rav_NT_10		Toa	*levesquei*	*dispansum*	11820	52,693	belemnite		2,85	-3,58	CPH		10	2,08	this study
Rav_NT_10		Toa	*levesquei*	*dispansum*	11820	52,693	belemnite		2,43	-3,68	CPH		11	2,05	this study
Rav_NT_10		Toa	*levesquei*	*dispansum*	11820	52,693	belemnite	altered	-1,80	-11,74	CPH		312	0,81	this study
Rav_NT_14		Toa	*levesquei*	*dispansum*	11825	52,697	belemnite		4,15	-3,57	CPH		8	2,13	this study
Rav_NT_14		Toa	*levesquei*	*dispansum*	11825	52,697	belemnite		3,79	-4,03	CPH		4	2,12	this study
Rav_NT_14		Toa	*levesquei*	*dispansum*	11825	52,697	belemnite		2,78	-3,72	CPH		7	2,05	this study
Rav_NT_13		Toa	*levesquei*	*dispansum*	11865	52,731	belemnite		2,69	-3,09	CPH		15	1,85	this study
Rav_NT_13		Toa	*levesquei*	*dispansum*	11865	52,731	belemnite		3,48	-2,92	CPH		15	1,90	this study
Rav_NT_13		Toa	*levesquei*	*dispansum*	11865	52,731	belemnite		3,64	-3,13	CPH		10	1,97	this study
Rav_MR_5		Toa	*levesquei*	*dispansum*	11880	52,744	belemnite		2,32	-3,72	CPH		8	1,89	this study
Rav_MR_5		Toa	*levesquei*	*dispansum*	11880	52,744	belemnite		3,25	-3,56	CPH		7	2,07	this study
Rav_MR_5		Toa	*levesquei*	*dispansum*	11880	52,744	belemnite		3,48	-3,45	CPH		97	2,11	this study
Rav_CVU_4		Toa	*levesquei*	*dispansum*	11925	52,782	belemnite		2,63	-3,94	CPH		35	1,76	this study
Rav_CVU_4		Toa	*levesquei*	*dispansum*	11925	52,782	belemnite		3,39	-3,84	CPH		27	1,92	this study
Rav_CVU_4		Toa	*levesquei*	*dispansum*	11925	52,782	belemnite		3,60	-3,20	CPH		51	1,90	this study
Rav_MR_13		Toa	*levesquei*	*dispansum*	11940	52,794	belemnite		2,58	-4,43	CPH		2	2,23	this study
Rav_MR_13		Toa	*levesquei*	*dispansum*	11940	52,794	belemnite		1,44	-2,08	CPH		2	2,01	this study
Rav_MR_13		Toa	*levesquei*	*dispansum*	11940	52,794	belemnite		2,19	-2,82	CPH		12	2,16	this study
Rav_NT_16		Toa	*levesquei*	*dispansum*	11970	52,819	belemnite		1,70	-3,43	CPH		24	1,68	this study
Rav_NT_16		Toa	*levesquei*	*dispansum*	11970	52,819	belemnite		1,95	-2,86	CPH		21	1,77	this study
Rav_NT_16		Toa	*levesquei*	*dispansum*	11970	52,819	belemnite		2,43	-2,98	CPH		8	1,92	this study
Rav_CVU_10		Toa	*levesquei*	*dispansum*	11975	52,824	belemnite		1,84	-3,10	CPH		31	1,83	this study
Rav_CVU_10		Toa	*levesquei*	*dispansum*	11975	52,824	belemnite		2,19	-3,15	CPH		25	1,92	this study
Rav_CVU_10		Toa	*levesquei*	*dispansum*	11975	52,824	belemnite		2,21	-3,78	CPH		40	1,76	this study
Rav_MR_16		Toa	*levesquei*	*dispansum*	11975	52,824	belemnite		3,04	-4,34	CPH		47	2,01	this study
Rav_MR_16		Toa	*levesquei*	*dispansum*	11975	52,824	belemnite		3,43	-4,98	CPH		35	2,05	this study
Rav_MR_16		Toa	*levesquei*	*dispansum*	11975	52,824	belemnite		3,21	-4,25	CPH		28	2,06	this study
Rav_MR_7		Toa	*levesquei*	*dispansum*	11975	52,824	belemnite		3,91	-3,69	CPH		7	2,34	this study
Rav_MR_7		Toa	*levesquei*	*dispansum*	11975	52,824	belemnite		3,28	-4,53	CPH		66	2,27	this study
Rav_MR_7		Toa	*levesquei*	*dispansum*	11975	52,824	belemnite		2,41	-4,48	CPH		34	2,14	this study
	base Bed 81	Toa	*levesquei*	*dispansum*	11985	52,832									
Rav_CK_22		Toa	*levesquei*	*dispansum*	11995	52,840	belemnite		2,17	-2,70	CPH		16	1,78	this study
Rav_CK_22		Toa	*levesquei*	*dispansum*	11995	52,840	belemnite		1,28	-3,08	CPH		23	1,55	this study
Rav_CK_22		Toa	*levesquei*	*dispansum*	11995	52,840	belemnite		2,24	-2,98	CPH		50	1,81	this study
Rav_CVU_13		Toa	*levesquei*	*dispansum*	12035	52,874	belemnite		2,42	-2,95	CPH		7	1,66	this study
Rav_CVU_13		Toa	*levesquei*	*dispansum*	12035	52,874	belemnite		2,69	-3,82	CPH		24	1,65	this study
Rav_CVU_13		Toa	*levesquei*	*dispansum*	12035	52,874	belemnite		3,54	-3,62	CPH		13	1,79	this study
Rav_ALE_006		Toa	*levesquei*	*dispansum*	12050	52,887	belemnite		3,46	-3,77	CPH		16	2,14	this study
Rav_ALE_006		Toa	*levesquei*	*dispansum*	12050	52,887	belemnite		2,69	-4,14	CPH		8	2,17	this study
Rav_ALE_006		Toa	*levesquei*	*dispansum*	12050	52,887	belemnite		2,76	-4,21	CPH		14	2,13	this study
Rav_ALE_11		Toa	*levesquei*	*dispansum*	12085	52,916	belemnite		1,45	-3,96	CPH		11	1,78	this study
Rav_ALE_11		Toa	*levesquei*	*dispansum*	12085	52,916	belemnite		1,79	-4,10	CPH		27	1,89	this study
Rav_ALE_11		Toa	*levesquei*	*dispansum*	12085	52,916	belemnite		2,72	-3,40	CPH		8	1,68	this study
Rav_ALE_12		Toa	*levesquei*	*dispansum*	12085	52,916	belemnite		3,93	-3,99	CPH		21	1,94	this study
Rav_ALE_12		Toa	*levesquei*	*dispansum*	12085	52,916	belemnite		2,69	-3,72	CPH		27	1,90	this study
Rav_ALE_12		Toa	*levesquei*	*dispansum*	12085	52,916	belemnite		2,81	-3,89	CPH		20	1,87	this study
Rav_ALE_19		Toa	*levesquei*	*dispansum*	12100	52,929	belemnite		1,92	-2,99	CPH		17	1,85	this study
Rav_ALE_19		Toa	*levesquei*	*dispansum*	12100	52,929	belemnite		2,49	-4,19	CPH		16	1,86	this study
Rav_ALE_16		Toa	*levesquei*	*dispansum*	12110	52,937	belemnite		2,49	-2,55	CPH	9,2	12	1,89	this study
Rav_ALE_16		Toa	*levesquei*	*dispansum*	12110	52,937	belemnite		2,83	-2,78	CPH	8,9	6	1,94	this study
Rav_ALE_16		Toa	*levesquei*	*dispansum*	12110	52,937	belemnite		2,55	-2,41	CPH	8,2	9	1,90	this study
Rav_ALE_2		Toa	*levesquei*	*dispansum*	12120	52,945	belemnite	altered	1,60	-4,75	CPH		300	2,10	this study
Rav_ALE_2		Toa	*levesquei*	*dispansum*	12120	52,945	belemnite		2,61	-3,66	CPH		74	2,28	this study
Rav_ALE_2		Toa	*levesquei*	*dispansum*	12120	52,945	belemnite	altered	1,37	-4,64	CPH		364	2,19	this study
Rav_CK_30		Toa	*levesquei*	*dispansum*	12120	52,945	belemnite		2,55	-2,95	CPH		5	1,95	this study
Rav_CK_30		Toa	*levesquei*	*dispansum*	12120	52,945	belemnite		2,50	-3,20	CPH		4	1,78	this study
Rav_CK_30		Toa	*levesquei*	*dispansum*	12120	52,945	belemnite		1,86	-3,32	CPH		30	1,78	this study
Rav_CK_32		Toa	*levesquei*	*dispansum*	12120	52,945	belemnite		4,23	-3,72	CPH		9	2,30	this study
Rav_CK_32		Toa	*levesquei*	*dispansum*	12120	52,945	belemnite		3,19	-3,77	CPH		9	2,24	this study

Rav_CK_32	Toa	*levesquei*	*dispansum*	12120	52,945	belemnite		3,17	-3,94	CPH		5	2,17	this study
Rav_ALE_003	Toa	*levesquei*	*dispansum*	12130	52,954	belemnite		3,27	-4,18	CPH		3	2,08	this study
Rav_ALE_003	Toa	*levesquei*	*dispansum*	12130	52,954	belemnite		2,64	-3,93	CPH		3	2,10	this study
Rav_ALE_003	Toa	*levesquei*	*dispansum*	12130	52,954	belemnite		2,88	-4,30	CPH		3	2,21	this study
base Bed 82	Toa			12185										
base *levesquei* sz	Toa			12185	53,000									
base Yellow Sandstone	Toa			12185										
base *moorei* sz	Toa			12635	54,000		approximated							
base *aalensis* sz	Toa			12850	55,000		approximated							
Rav_CK_40	Toa	*levesquei*	*aalensis*	12978	55,853	belemnite		1,84	-3,91	CPH		21	2,37	this study
Rav_CK_40	Toa	*levesquei*	*aalensis*	12978	55,853	belemnite		2,19	-4,40	CPH		5	2,36	this study
Rav_CK_40	Toa	*levesquei*	*aalensis*	12978	55,853	belemnite		2,48	-4,21	CPH		4	2,20	this study
Rav_CK_43	Toa	*levesquei*	*aalensis*	12995	55,967	belemnite		0,49	-4,46	CPH		8	1,81	this study
Rav_CK_43	Toa	*levesquei*	*aalensis*	12995	55,967	belemnite		0,16	-4,94	CPH		15	1,78	this study
Rav_CK_43	Toa	*levesquei*	*aalensis*	12995	55,967	belemnite		0,16	-4,74	CPH		10	1,72	this study
Rav_MR_19	Toa	*levesquei*	*aalensis*	13000	55,000	belemnite		2,75	-4,57	CPH		15	2,38	this study
Rav_MR_19	Toa	*levesquei*	*aalensis*	13000	55,000	belemnite		2,81	-4,46	CPH		11	2,36	this study
base opalinum z	Aal			13000										
base Dogger	Aal			13000										
base Aalenian	Aal			13000										
Rav_CK_41	Aal	*opalinum*	-	13010	56,009	belemnite		2,05	-4,11	CPH		5	1,78	this study
Rav_CK_41	Aal	*opalinum*	-	13010	56,009	belemnite		1,98	-3,11	CPH		6	1,77	this study
Rav_CK_41	Aal	*opalinum*	-	13010	56,009	belemnite		2,08	-3,39	CPH		34	1,87	this study
Rav_ALE_21	Aal	*opalinum*	-	13065	56,059	belemnite		1,03	-3,78	CPH		79	1,84	this study
Rav_ALE_21	Aal	*opalinum*	-	13065	56,059	belemnite		2,11	-3,68	CPH		68	1,83	this study
Rav_ALE_21	Aal	*opalinum*	-	13065	56,059	belemnite		1,81	-4,43	CPH		80	1,73	this study
Rav_NT_20	Aal	*opalinum*	-	13075	56,068	belemnite		2,20	-3,77	CPH		76	1,83	this study
Rav_NT_20	Aal	*opalinum*	-	13075	56,068	belemnite		1,92	-3,77	CPH		84	1,91	this study
Rav_NT_20	Aal	*opalinum*	-	13075	56,068	belemnite		1,97	-3,88	CPH		76	1,91	this study
Rav_ALE_22	Aal	*opalinum*	-	13135	56,123	belemnite		1,07	-3,17	CPH		32	1,72	this study
Rav_ALE_22	Aal	*opalinum*	-	13135	56,123	belemnite		1,03	-2,56	CPH		40	1,66	this study
Rav_ALE_22	Aal	*opalinum*	-	13135	56,123	belemnite		1,36	-2,18	CPH		13	1,84	this study
Rav_CK_46	Aal	*opalinum*	-	13150	56,136	belemnite		1,85	-2,59	CPH		6	1,67	this study
Rav_CK_46	Aal	*opalinum*	-	13150	56,136	belemnite		2,17	-2,90	CPH		6	1,82	this study
Rav_CK_46	Aal	*opalinum*	-	13150	56,136	belemnite		2,84	-4,53	CPH		78	1,91	this study
Rav_CK_47	Aal	*opalinum*	-	13155	56,141	belemnite		2,75	-4,03	CPH		45	1,93	this study
Rav_CK_47	Aal	*opalinum*	-	13155	56,141	belemnite		2,42	-4,09	CPH		16	1,95	this study
Rav_CK_47	Aal	*opalinum*	-	13155	56,141	belemnite		1,73	-4,23	CPH		60	1,96	this study
Rav_CK_48	Aal	*opalinum*	-	13160	56,145	belemnite		3,40	-3,60	CPH		6	2,07	this study
Rav_CK_48	Aal	*opalinum*	-	13160	56,145	belemnite		3,43	-3,10	CPH		4	2,06	this study
Rav_CK_48	Aal	*opalinum*	-	13160	56,145	belemnite		2,44	-3,55	CPH		4	1,94	this study
Rav_CK_49	Aal	*opalinum*	-	13165	56,150	belemnite		2,60	-3,21	CPH		25	2,04	this study
Rav_CK_49	Aal	*opalinum*	-	13165	56,150	belemnite	altered	2,45	-7,44	CPH		83	1,17	this study
Rav_CK_49	Aal	*opalinum*	-	13165	56,150	belemnite	altered	1,18	-8,17	CPH		147	1,31	this study
Rav_ALE_23	Aal	*opalinum*	-	13170	56,155	belemnite		1,12	-1,89	CPH		19	1,70	this study
Rav_ALE_23	Aal	*opalinum*	-	13170	56,155	belemnite		1,37	-1,25	CPH		18	1,68	this study
Rav_ALE_23	Aal	*opalinum*	-	13170	56,155	belemnite		1,39	-2,13	CPH		90	1,64	this study
Rav_MR_20	Aal	*opalinum*	-	13200	56,182	belemnite		2,83	-3,42	CPH	10,6	8	1,88	this study
Rav_MR_20	Aal	*opalinum*	-	13200	56,182	belemnite		2,98	-3,23	CPH	10,7	12	1,99	this study
Rav_MR_20	Aal	*opalinum*	-	13200	56,182	belemnite		2,19	-3,91	CPH	10,7	20	1,90	this study
top opalinum z	Aal			14100										
top Dogger	Aal			14100										

Hundale Point

base Aalenian				-1120			approximated							
base Dogger				-1120			approximated							
Aal-Baj boundary				5240			approximated							
base *discites* z	Baj			5240			approximated							
base *sauzei* z	Baj			11400			approximated							
Hun CK 3	Baj	*sauzei*	-	12520	70,699	bivalve unident.	altered	-0,21	-12,12	CPH	21,5	2390	1,22	this study
Hun CK 5	Baj	*sauzei*	-	12795	70,870	bivalve unident.		3,94	-0,17	CPH	2,5	192	0,78	this study
Hun CK 7	Baj	*sauzei*	-	12800	70,873	belemnite	altered	-0,10	-9,46	CPH	19,8	854	1,00	this study
Hun CK 7	Baj	*sauzei*	-	12800	70,873	bivalve unident.		3,34	-2,25	CPH	7,8	615	1,14	this study
Hun CK 35	Baj	*sauzei*	-	12975	70,983	oyster		4,35	-0,68	CPH	4,0	36	0,65	this study
Hun CK 8	Baj	*sauzei*	-	12980	70,986	oyster		3,83	-3,16	CPH	4,2	154	0,71	this study
Hun CK 36	Baj	*sauzei*	-	12985	70,989	oyster		4,14	-1,02	CPH	1,1	81	0,68	this study
Hun CK 37	Baj	*sauzei*	-	13000	70,998	pinnid	altered	-0,82	-13,34	CPH	13,0	784	0,77	this study
base *humphriesianum* z	Baj			13003			approximated							
base *romani* sz	Baj			13003			approximated							
Hun CK 18	Baj	*humphriesianum*	*romani*	13015	71,014	belemnite	altered	0,10	-15,91	CPH	5,6	47	0,75	this study
Hun CK 19	Baj	*humphriesianum*	*romani*	13020	71,019	pectinid	altered	1,96	-4,30	CPH	10,2	501	1,33	this study
Hun CK 14	Baj	*humphriesianum*	*romani*	13030	71,030	oyster		3,50	-2,96	CPH	1,7	73	0,77	this study
Hun CK 22	Baj	*humphriesianum*	*romani*	13135	71,149	pectinid		5,41	-0,23	CPH	0,5	63	0,56	this study
Hun CK 16	Baj	*humphriesianum*	*romani*	13140	71,154	oyster		4,13	-0,93	CPH	1,4	48	0,80	this study
Hun CK 16	Baj	*humphriesianum*	*romani*	13140	71,154	oyster		4,16	-0,83	CPH	1,4	42	0,75	this study
Hun CK 9	Baj	*humphriesianum*	*romani*	13150	71,166	pinnid		3,64	-2,26	CPH	9,1	58	0,85	this study
Hun CK 11	Baj	*humphriesianum*	*romani*	13150	71,166	pinnid		3,89	-2,16	CPH	7,9	50	0,90	this study

Hun MEJ 6	Baj	*humphriesianum*	*romani*	13150	71,166	oyster			3,50	-3,02	CPH	2,1	84	0,75	this study
Hun CK 24 A	Baj	*humphriesianum*	*romani*	13155	71,171	oyster			4,07	-1,32	CPH	2,5	46	0,87	this study
Hun CK 24 B	Baj	*humphriesianum*	*romani*	13155	71,171	oyster			4,32	-1,10	CPH	1,6	31	0,75	this study
Hun CK 25	Baj	*humphriesianum*	*romani*	13160	71,177	oyster			4,33	-2,53	CPH	2,2	276	0,79	this study
Hun CK 26	Baj	*humphriesianum*	*romani*	13165	71,183	oyster		altered	0,21	-9,88	CPH	20,1	561	0,77	this study
Hun CK 27	Baj	*humphriesianum*	*romani*	13260	71,290	oyster			4,32	-1,83	CPH	2,9	177	0,74	this study
Hun CK 12	Baj	*humphriesianum*	*romani*	13280	71,312	bivalve unident.			3,91	-1,88	CPH	7,7	359	1,04	this study
Hun CK 12	Baj	*humphriesianum*	*romani*	13280	71,312	bivalve unident.			3,90	-1,98	CPH	8,4	413	1,07	this study
Hun CK 28	Baj	*humphriesianum*	*romani*	13290	71,324	bivalve unident.			2,35	-2,57	CPH	6,1	285	1,19	this study
Hun CK 29	Baj	*humphriesianum*	*romani*	13290	71,324	bivalve unident.		altered	3,00	-3,38	CPH	12,1	713	1,20	this study
Hun CK 30	Baj	*humphriesianum*	*romani*	13325	71,363	bivalve unident.		good	3,21	-2,43	CPH	11,0	308	1,35	this study
Hun CK 31	Baj	*humphriesianum*	*romani*	13350	71,391	belemnite			3,20	-1,26	CPH	6,9	15	1,59	this study
Hun CK 32	Baj	*humphriesianum*	*romani*	13350	71,391	belemnite		good	3,47	-1,43	CPH	9,1	6	1,90	this study
Hun CK 32	Baj	*humphriesianum*	*romani*	13350	71,391	oyster		good	3,87	-1,40	CPH	2,3	254	0,83	this study
Hun CK 33	Baj	*humphriesianum*	*romani*	13350	71,391	belemnite		altered	-0,42	-15,14	CPH	6,8	47	0,71	this study
Hun CK 34	Baj	*humphriesianum*	*romani*	13350	71,391	bivalve unident.			3,30	-1,98	CPH	12,0	190	0,82	this study
Hun CK 34	Baj	*humphriesianum*	*romani*	13350	71,391	bivalve unident.			3,48	-1,97	CPH	6,3	143	0,82	this study
Hun NT 23	Baj	*humphriesianum*	*romani*	13375	71,419	oyster			4,40	-1,65	CPH	2,3	119	0,86	this study
Hun CVU 6	Baj	*humphriesianum*	*romani*	13720	71,808	pinnid			3,93	-0,35	CPH	6,3	238	1,07	this study
Hun ALE 6	Baj	*humphriesianum*	*romani*	13755	71,848	bivalve unident.			4,11	-1,33	CPH	8,7	375	1,09	this study
Hun MR 23	Baj	*humphriesianum*	*romani*	13765	71,859	bivalve unident.			3,80	-0,74	CPH	7,9	517	1,10	this study
Hun NT 24	Baj	*humphriesianum*	*romani*	13800	71,899	oyster			4,19	-2,07	CPH	3,1	61	0,61	this study
Hun NT 24	Baj	*humphriesianum*	*romani*	13800	71,899	oyster			3,90	-2,17	CPH	2,6	190	0,58	this study
Hun NT 24	Baj	*humphriesianum*	*romani*	13800	71,899	belemnite			2,87	-3,08	CPH	12,6	182	1,90	this study
Hun MR 24	Baj	*humphriesianum*	*romani*	13810	71,910	belemnite		altered	2,51	-8,95	CPH	4,2	60	0,91	this study
Hun MR 24	Baj	*humphriesianum*	*romani*	13810	71,910	belemnite		altered	2,63	-9,29	CPH	4,2	39	0,91	this study
Hun CVU 9	Baj	*humphriesianum*	*romani*	13870	71,977	bivalve unident.			3,39	0,47	CPH	1,4	31	0,57	this study
base *humphriesianum* sz	Baj			13890				approximated							
Hun CVU 7	Baj	*humphriesianum*	*humphriesianum*	13900	72,023	pinnid			4,35	-0,98	CPH	6,8	98	1,25	this study
Hun NT 26	Baj	*humphriesianum*	*humphriesianum*	13975	72,196	bivalve unident.		altered	4,42	-0,44	CPH	6,8	235	0,68	this study
Hun NT 26	Baj	*humphriesianum*	*humphriesianum*	13975	72,196	bivalve unident.		altered	4,55	-1,31	CPH	4,4	216	0,69	this study
Hun NT 27	Baj	*humphriesianum*	*humphriesianum*	14015	72,289	oyster			5,47	1,87	CPH	6,7	41	0,62	this study
Hun NT 27	Baj	*humphriesianum*	*humphriesianum*	14015	72,289	oyster			5,44	1,63	CPH	3,9	54	0,61	this study
Hun NT 27	Baj	*humphriesianum*	*humphriesianum*	14015	72,289	oyster			5,51	1,43	CPH	2,1	48	0,60	this study
Hun CVU 11	Baj	*humphriesianum*	*humphriesianum*	14050	72,370	bivalve unident.			3,27	-1,88	CPH	8,2	412	1,08	this study
Hun ALE 3	Baj	*humphriesianum*	*humphriesianum*	14265	72,866	bivalve unident.		altered	4,04	-2,53	CPH	12,0	186	1,25	this study
Hun CU 5	Baj	*humphriesianum*	*humphriesianum*	14265	72,866	bivalve unident.		altered	3,15	-2,85	CPH	12,2	173	1,25	this study
Hun ALE 3	Baj	*humphriesianum*	*humphriesianum*	14265	72,866	bivalve unident.		altered	3,75	-2,38	CPH	12,3	214	1,21	this study
base *blagdeni* sz	Baj			14323				approximated							

<u>literature data</u>

Saltwick Bay

base *falciferum* sz				2235						Li et al. 2012[7]
Y06 43/7	Toa	*falciferum*	*falciferum*	2671	45,216	belemnite	*A. subtenuis*	1,31	-1,29	Li et al. 2012[7]
Y06 43/200	Toa	*falciferum*	*falciferum*	2864	45,312	belemnite	*A. subtenuis*	2,15	-1,36	Li et al. 2012[7]
Y06A 43/307	Toa	*falciferum*	*falciferum*	2971	45,365	belemnite	*A. subtenuis*	2,15	-0,89	Li et al. 2012[7]
Y06A 43/310	Toa	*falciferum*	*falciferum*	2974	45,366	belemnite	*Y. simpsoni*	2,91	-2,02	Li et al. 2012[7]
Y06B 43/310	Toa	*falciferum*	*falciferum*	2974	45,366	belemnite	*Y. tubularis*	3,29	-2,41	Li et al. 2012[7]
Y06D 43/310	Toa	*falciferum*	*falciferum*	2974	45,366	belemnite	*Y. simpsoni*	3,07	-2,40	Li et al. 2012[7]
Y06 43/326	Toa	*falciferum*	*falciferum*	2990	45,374	belemnite	*Y. simpsoni*	1,69	-2,13	Li et al. 2012[7]
Y06 43/362	Toa	*falciferum*	*falciferum*	3026	45,392	belemnite	*Y. simpsoni*	2,43	-1,05	Li et al. 2012[7]
Y06 43/387	Toa	*falciferum*	*falciferum*	3051	45,404	belemnite	*Y. simpsoni*	3,35	-2,04	Li et al. 2012[7]
Y06 43/392	Toa	*falciferum*	*falciferum*	3056	45,407	belemnite	*Y. simpsoni*	3,13	-2,50	Li et al. 2012[7]
Y06A 43/510	Toa	*falciferum*	*falciferum*	3174	45,465	belemnite	*Y. tubularis*	3,17	-2,53	Li et al. 2012[7]
Y06B 43/510	Toa	*falciferum*	*falciferum*	3174	45,465	belemnite	*Y. simpsoni*	3,42	-2,94	Li et al. 2012[7]
Y06 43/515	Toa	*falciferum*	*falciferum*	3179	45,468	belemnite	*A. subtenuis*	3,03	-2,00	Li et al. 2012[7]
Y06B 43/516	Toa	*falciferum*	*falciferum*	3180	45,468	belemnite	*Y. simpsoni*	2,64	-2,38	Li et al. 2012[7]
Y06C 43/516	Toa	*falciferum*	*falciferum*	3180	45,468	belemnite	*A. subtenuis*	2,79	-1,78	Li et al. 2012[7]
Y06B 43/580	Toa	*falciferum*	*falciferum*	3244	45,500	belemnite	*A. subtenuis*	2,06	-1,27	Li et al. 2012[7]
Y06 43/641	Toa	*falciferum*	*falciferum*	3305	45,530	belemnite	*Y. simpsoni*	3,83	-3,21	Li et al. 2012[7]
Y06 43/671	Toa	*falciferum*	*falciferum*	3335	45,545	belemnite	*S. dorsalis*	4,56	-2,03	Li et al. 2012[7]
Y06A 43/691	Toa	*falciferum*	*falciferum*	3355	45,555	belemnite	*S. dorsalis*	4,54	-2,29	Li et al. 2012[7]
Y06B 43/691	Toa	*falciferum*	*falciferum*	3355	45,555	belemnite	*S. dorsalis*	4,51	-2,20	Li et al. 2012[7]
Y06 43/698	Toa	*falciferum*	*falciferum*	3362	45,558	belemnite	*S. dorsalis*	4,08	-2,17	Li et al. 2012[7]
Y06 43/718	Toa	*falciferum*	*falciferum*	3382	45,568	belemnite	*A. subtenuis*	1,75	-1,15	Li et al. 2012[7]
Y06 45/0	Toa	*falciferum*	*falciferum*	3446	45,600	belemnite	*S. dorsalis*	4,63	-2,53	Li et al. 2012[7]
Y06 47/307	Toa	*falciferum*	*falciferum*	4101	45,925	belemnite	*A. subtenuis*	1,44	-1,09	Li et al. 2012[7]
Y06 47/379	Toa	*falciferum*	*falciferum*	4173	45,960	belemnite	*A. subtenuis*	4,19	-1,96	Li et al. 2012[7]
Y06 47/416	Toa	*falciferum*	*falciferum*	4210	45,979	belemnite	*S. dorsalis*	2,92	-2,29	Li et al. 2012[7]
Y06 47/485	Toa	*falciferum*	*falciferum*	4279	45,013	belemnite	*A. vulgaris*	4,29	-3,30	Li et al. 2012[7]
Y06 47/492	Toa	*falciferum*	*falciferum*	4286	45,016	belemnite	*A. vulgaris*	4,22	-3,10	Li et al. 2012[7]
Y06A 47/520	Toa	*falciferum*	*falciferum*	4310	45,028	belemnite	*S. dorsalis*	4,64	-2,30	Li et al. 2012[7]
Y06B 47/520	Toa	*falciferum*	*falciferum*	4310	45,028	belemnite	*A. subtenuis*	2,16	-1,31	Li et al. 2012[7]
Y06 47/529	Toa	*falciferum*	*falciferum*	4320	45,033	belemnite	*A. subtenuis*	3,18	-1,38	Li et al. 2012[7]
Y06 47/526	Toa	*falciferum*	*falciferum*	4320	45,033	belemnite	*S. dorsalis*	4,60	-2,23	Li et al. 2012[7]

base *commune* sz	Toa			4253						Li et al. 2012[7]
Y06 49/25	Toa	*bifrons*	*commune*	4400	46,072	belemnite	*A. subtenuis*	2,20	-2,08	Li et al. 2012[7]
Y06 49/22	Toa	*bifrons*	*commune*	4400	46,072	belemnite	*A. subtenuis*	2,88	-2,30	Li et al. 2012[7]
Y06 49/30	Toa	*bifrons*	*commune*	4410	46,076	belemnite	*A. vulgaris*	3,62	-3,29	Li et al. 2012[7]
Y06 49/63	Toa	*bifrons*	*commune*	4440	46,091	belemnite	*A. subtenuis*	3,02	-2,31	Li et al. 2012[7]
Y06 49/420	Toa	*bifrons*	*commune*	4800	46,266	belemnite	*A. subtenuis*	2,47	-2,02	Li et al. 2012[7]
Y06 49/475	Toa	*bifrons*	*commune*	4850	46,291	belemnite	*A. subtenuis*	3,38	-2,72	Li et al. 2012[7]
Y06 49/519	Toa	*bifrons*	*commune*	4900	46,315	belemnite	*A. subtenuis*	2,92	-2,60	Li et al. 2012[7]
Y06 49/592	Toa	*bifrons*	*commune*	4970	46,349	belemnite	*A. subtenuis*	3,29	-2,65	Li et al. 2012[7]
Y06A 51 base	Toa	*bifrons*	*commune*	5000	46,364	belemnite	*A. subtenuis*	2,99	-3,04	Li et al. 2012[7]
Y06B 51 base	Toa	*bifrons*	*commune*	5000	46,364	belemnite	*A. subtenuis*	2,85	-2,67	Li et al. 2012[7]
Y06A 49/620	Toa	*bifrons*	*commune*	5000	46,364	belemnite	*A. subtenuis*	2,89	-2,51	Li et al. 2012[7]
Y06B 49/620	Toa	*bifrons*	*commune*	5000	46,364	belemnite	*A. subtricissus*	2,80	-3,26	Li et al. 2012[7]
Y06C 49/620	Toa	*bifrons*	*commune*	5000	46,364	belemnite	*A. subtenuis*	3,22	-2,56	Li et al. 2012[7]
Y06 51/60	Toa	*bifrons*	*commune*	5070	46,398	belemnite	*A. vulgaris*	3,95	-3,05	Li et al. 2012[7]
Y06 51/78	Toa	*bifrons*	*commune*	5090	46,407	belemnite	*A. subtenuis*	1,39	-1,64	Li et al. 2012[7]
Y06A 51/75	Toa	*bifrons*	*commune*	5090	46,407	belemnite	*S. dorsalis*	2,73	-0,80	Li et al. 2012[7]
Y06B 51/75	Toa	*bifrons*	*commune*	5090	46,407	belemnite	*S. dorsalis*	4,67	-2,50	Li et al. 2012[7]
Y06 51/110	Toa	*bifrons*	*commune*	5120	46,422	belemnite	*A. subtenuis*	2,66	-2,20	Li et al. 2012[7]

Staithes, Hawsker Bottoms, Kettleness, Port Mulgrave, Runswick, Saltwick Bay, Blea Wyke

base of *margaritatus* z	Pli									McArthur et al. 2000[8]
base of *stokesi* sz	Pli			-59,59						McArthur et al. 2000[8]
St 131	Pli	*margaritatus*	*stokesi*	-49,88	35,529	belemnite		2,45	-2,98	McArthur et al. 2000[8]
St 130 (1)	Pli	*margaritatus*	*stokesi*	-43,08	35,900	belemnite		2,57	-2,91	McArthur et al. 2000[8]
St 129	Pli	*margaritatus*	*stokesi*	-43,03	35,902	belemnite		2,77	-2,38	McArthur et al. 2000[8]
base of *subnodosus* sz	Pli			-41,24						McArthur et al. 2000[8]
St 128A	Pli	*margaritatus*	*subnodosus*	-40,17	36,151	belemnite		2,28	-1,08	McArthur et al. 2000[8]
St 127A	Pli	*margaritatus*	*subnodosus*	-37,18	36,572	belemnite		2,94	-1,38	McArthur et al. 2000[8]
base of *gibbosus* sz	Pli			-34,14						McArthur et al. 2000[8]
St 126B	Pli	*margaritatus*	*gibbosus*	-33,93	37,025	belemnite		3,00	-0,60	McArthur et al. 2000[8]
St 123	Pli	*margaritatus*	*gibbosus*	-30,80	37,396	belemnite		3,54	-1,44	McArthur et al. 2000[8]
St 121	Pli	*margaritatus*	*gibbosus*	-28,90	37,622	belemnite		2,04	-1,97	McArthur et al. 2000[8]
St 120	Pli	*margaritatus*	*gibbosus*	-27,38	37,802	belemnite		2,41	-0,49	McArthur et al. 2000[8]
St 119B	Pli	*margaritatus*	*gibbosus*	-26,11	37,953	belemnite		3,36	-1,98	McArthur et al. 2000[8]
base of *spinatum* z	Pli			-25,71						McArthur et al. 2000[8]
base of *apyrenum* sz	Pli			-25,71						McArthur et al. 2000[8]
St 102A	Pli	*spinatum*	*apyrenum*	-24,90	38,121	belemnite		1,50	0,10	McArthur et al. 2000[8]
St 116D	Pli	*spinatum*	*apyrenum*	-24,74	38,145	belemnite		2,42	-2,64	McArthur et al. 2000[8]
HB 17	Pli	*spinatum*	*apyrenum*	-23,98	38,259	belemnite		2,01	-1,58	McArthur et al. 2000[8]
HB 16	Pli	*spinatum*	*apyrenum*	-20,81	38,735	belemnite		0,69	-2,91	McArthur et al. 2000[8]
base of *hawskerense* sz	Pli			-19,04						McArthur et al. 2000[8]
HB 13	Pli	*spinatum*	*hawskerense*	-17,63	39,257	belemnite		1,04	-3,53	McArthur et al. 2000[8]
HB 8	Pli	*spinatum*	*hawskerense*	-15,92	39,569	belemnite		2,21	-2,89	McArthur et al. 2000[8]
HB 11	Pli	*spinatum*	*hawskerense*	-14,10	39,901	belemnite		2,21	-2,89	McArthur et al. 2000[8]
St 109	Pli	*spinatum*	*hawskerense*	-13,98	39,923	belemnite		1,64		McArthur et al. 2000[8]
base of *tenuicostatum* z	Toa				40,000					McArthur et al. 2000[8]
base of *paltum* sz	Toa			-13,56	40,000			1,29	-1,04	McArthur et al. 2000[8]
HB 1	Toa	*tenuicostatum*	*paltum*	-13,32	40,062	belemnite		1,09	-1,09	McArthur et al. 2000[8]
HB 2	Toa	*tenuicostatum*	*paltum*	-13,25	40,080	belemnite		1,46	-0,90	McArthur et al. 2000[8]
HB 4	Toa	*tenuicostatum*	*paltum*	-13,09	40,122	belemnite		1,63	-1,37	McArthur et al. 2000[8]
HB 3	Toa	*tenuicostatum*	*paltum*	-13,07	40,127	belemnite		1,59	-0,53	McArthur et al. 2000[8]
HB 7	Toa	*tenuicostatum*	*paltum*	-12,53	40,267	belemnite		2,10	-1,02	McArthur et al. 2000[8]
St 104	Toa	*tenuicostatum*	*paltum*	-11,69	40,484	belemnite		2,82	-0,57	McArthur et al. 2000[8]
K 107	Toa	*tenuicostatum*	*paltum*	-9,91	40,946	belemnite		1,96	-1,27	McArthur et al. 2000[8]
K 105A	Toa	*tenuicostatum*	*paltum*	-9,77	40,982	belemnite		3,17	0,03	McArthur et al. 2000[8]
base of *clevelandicum* sz	Toa			-9,70						McArthur et al. 2000[8]
K 108B	Toa	*clevelandicum*	*tenuicostatum*	-8,08	42,007	belemnite		2,33	-0,45	McArthur et al. 2000[8]
base of *tenuicostatum* sz	Toa			-8,10						McArthur et al. 2000[8]
K 111B	Toa	*tenuicostatum*	*tenuicostatum*	-6,07	42,741	belemnite		2,49	-1,05	McArthur et al. 2000[8]
K 111C	Toa	*tenuicostatum*	*tenuicostatum*	-6,07	42,741	belemnite		2,53	-0,65	McArthur et al. 2000[8]
base of *semicelatum* sz	Toa			-5,36						McArthur et al. 2000[8]
K 112B	Toa	*tenuicostatum*	*semicelatum*	-4,54	43,153	belemnite		3,88	0,87	McArthur et al. 2000[8]
PM 102	Toa	*tenuicostatum*	*semicelatum*	-4,06	43,243	belemnite		2,41	0,69	McArthur et al. 2000[8]
K 121	Toa	*tenuicostatum*	*semicelatum*	-3,18	43,407	belemnite		2,20	-1,55	McArthur et al. 2000[8]
PM 101	Toa	*tenuicostatum*	*semicelatum*	-2,90	43,459	belemnite		2,29	-1,31	McArthur et al. 2000[8]
K 118A	Toa	*tenuicostatum*	*semicelatum*	-2,08	43,612	belemnite		3,18	0,08	McArthur et al. 2000[8]
K 117	Toa	*tenuicostatum*	*semicelatum*	-1,68	43,687	belemnite		2,55	-1,09	McArthur et al. 2000[8]
PM 108	Toa	*tenuicostatum*	*semicelatum*	-1,00	43,813	belemnite		1,92	-1,49	McArthur et al. 2000[8]
PM 112A	Toa	*tenuicostatum*	*semicelatum*	-0,80	43,851	belemnite		3,52	-1,72	McArthur et al. 2000[8]
PM 113	Toa	*tenuicostatum*	*semicelatum*	-0,65	43,879	belemnite		2,91	-2,76	McArthur et al. 2000[8]
PM 106	Toa	*tenuicostatum*	*semicelatum*	-0,45	43,916	belemnite		1,68	-3,64	McArthur et al. 2000[8]
base of *falciferum* z	Toa			0,00	44,000					McArthur et al. 2000[8]
base of *exaratum* sz	Toa			0,00	44,000					McArthur et al. 2000[8]
PM 107	Toa	*falciferum*	*exaratum*	0,15	44,021	belemnite		2,00	-3,80	McArthur et al. 2000[8]
PM 21	Toa	*falciferum*	*exaratum*	0,90	44,125	belemnite		3,31		McArthur et al. 2000[8]
PM 109	Toa	*falciferum*	*exaratum*	1,80	44,250	belemnite		3,09		McArthur et al. 2000[8]

PM 111	Toa	*falciferum*	*exaratum*	2,25	44,313	belemnite	2,00	-3,51	McArthur et al. 2000[8]
PM 105	Toa	*falciferum*	*exaratum*	2,45	44,340	belemnite	1,82	-4,83	McArthur et al. 2000[8]
PM 3	Toa	*falciferum*	*exaratum*	3,60	44,500	belemnite	2,36	-4,31	McArthur et al. 2000[8]
S 2	Toa	*falciferum*	*exaratum*	4,70	44,653	belemnite	4,34	-3,50	McArthur et al. 2000[8]
PM 104	Toa	*falciferum*	*exaratum*	4,70	44,653	belemnite	3,29	-4,27	McArthur et al. 2000[8]
R 4B	Toa	*falciferum*	*exaratum*	4,90	44,681	belemnite	5,29	-2,80	McArthur et al. 2000[8]
S 1A	Toa	*falciferum*	*exaratum*	5,40	44,750	belemnite	3,63	-3,90	McArthur et al. 2000[8]
PM 8	Toa	*falciferum*	*exaratum*	5,70	44,792	belemnite	4,08	-4,65	McArthur et al. 2000[8]
PM 2C	Toa	*falciferum*	*exaratum*	6,55	44,910	belemnite	6,36	-3,84	McArthur et al. 2000[8]
PM 7	Toa	*falciferum*	*exaratum*	6,80	44,944	belemnite	5,97	-3,24	McArthur et al. 2000[8]
base of *falciferum* sz	Toa			7,20					McArthur et al. 2000[8]
PM 18	Toa	*falciferum*	*falciferum*	7,20	45,000	belemnite	5,63	-3,96	McArthur et al. 2000[8]
PM 13	Toa	*falciferum*	*falciferum*	7,30	45,004	belemnite	5,55	-4,01	McArthur et al. 2000[8]
PM 16	Toa	*falciferum*	*falciferum*	7,50	45,013	belemnite	3,65	-3,04	McArthur et al. 2000[8]
PM 15	Toa	*falciferum*	*falciferum*	7,80	45,026	belemnite	4,72	-4,53	McArthur et al. 2000[8]
PM 17	Toa	*falciferum*	*falciferum*	8,20	45,043	belemnite	4,38	-4,18	McArthur et al. 2000[8]
PM 20	Toa	*falciferum*	*falciferum*	8,50	45,056	belemnite	4,12	-2,51	McArthur et al. 2000[8]
PM 103	Toa	*falciferum*	*falciferum*	9,90	45,117	belemnite	5,50	-2,57	McArthur et al. 2000[8]
R 5	Toa	*falciferum*	*falciferum*	12,00	45,209	belemnite	4,69	-3,09	McArthur et al. 2000[8]
R 10	Toa	*falciferum*	*falciferum*	12,60	45,235	belemnite	4,10	-2,99	McArthur et al. 2000[8]
R 7A	Toa	*falciferum*	*falciferum*	14,00	45,296	belemnite	2,96	-2,07	McArthur et al. 2000[8]
R 6A	Toa	*falciferum*	*falciferum*	14,70	45,326	belemnite	2,43	-2,47	McArthur et al. 2000[8]
R 8	Toa	*falciferum*	*falciferum*	15,60	45,365	belemnite	2,32	-1,51	McArthur et al. 2000[8]
S 9B	Toa	*falciferum*	*falciferum*	16,10	45,387	belemnite	2,25	-1,84	McArthur et al. 2000[8]
S 11A	Toa	*falciferum*	*falciferum*	18,60	45,495	belemnite	2,47	-1,64	McArthur et al. 2000[8]
S 13	Toa	*falciferum*	*falciferum*	20,40	45,574	belemnite	4,47	-3,30	McArthur et al. 2000[8]
S 14A	Toa	*falciferum*	*falciferum*	21,00	45,600	belemnite	5,10	-2,58	McArthur et al. 2000[8]
S 306	Toa	*falciferum*	*falciferum*	21,90	45,639	belemnite	4,82	-2,69	McArthur et al. 2000[8]
S 321	Toa	*falciferum*	*falciferum*	23,20	45,695	belemnite	4,70	-2,76	McArthur et al. 2000[8]
S 319B	Toa	*falciferum*	*falciferum*	27,00	45,860	belemnite	4,58	-2,29	McArthur et al. 2000[8]
S 315	Toa	*falciferum*	*falciferum*	28,60	45,930	belemnite	2,83	-1,99	McArthur et al. 2000[8]
S 313A	Toa	*falciferum*	*falciferum*	30,00	45,991	belemnite	4,14	-3,32	McArthur et al. 2000[8]
base of the *bifrons* z	Toa			30,21	46,000				McArthur et al. 2000[8]
base of the *commune* sz	Toa			30,21	46,000				McArthur et al. 2000[8]
S 343	Toa	*bifrons*	*commune*	31,78	46,085	belemnite	3,71	-2,63	McArthur et al. 2000[8]
S 342	Toa	*bifrons*	*commune*	33,26	46,165	belemnite	3,89	-3,86	McArthur et al. 2000[8]
S 327	Toa	*bifrons*	*commune*	36,41	46,336	belemnite	2,69	-2,94	McArthur et al. 2000[8]
S 401	Toa	*bifrons*	*commune*	37,20	46,379	belemnite	4,29	-2,50	McArthur et al. 2000[8]
S 406	Toa	*bifrons*	*commune*	39,50	46,503	belemnite	3,46	-3,71	McArthur et al. 2000[8]
S 413A	Toa	*bifrons*	*commune*	42,00	46,639	belemnite	4,06	-2,78	McArthur et al. 2000[8]
S 414B	Toa	*bifrons*	*commune*	43,90	46,742	belemnite	3,42	-3,91	McArthur et al. 2000[8]
S 418C	Toa	*bifrons*	*commune*	45,60	46,834	belemnite	4,19	-2,69	McArthur et al. 2000[8]
S 421A	Toa	*bifrons*	*commune*	48,10	46,969	belemnite	4,42	-3,02	McArthur et al. 2000[8]
base of the *fibulatum* sz	Toa			48,67					McArthur et al. 2000[8]
S 422B	Toa	*bifrons*	*fibulatum*	48,72	47,004	belemnite	2,86	-2,44	McArthur et al. 2000[8]
S 423A	Toa	*bifrons*	*fibulatum*	49,52	47,070	belemnite	3,09	-3,27	McArthur et al. 2000[8]
S 428	Toa	*bifrons*	*fibulatum*	51,62	47,241	belemnite	3,30	-2,13	McArthur et al. 2000[8]
S 432A	Toa	*bifrons*	*fibulatum*	52,90	47,346	belemnite	2,73	-3,75	McArthur et al. 2000[8]
S 435A	Toa	*bifrons*	*fibulatum*	54,19	47,451	belemnite	2,67	-3,98	McArthur et al. 2000[8]
P 29	Toa	*bifrons*	*fibulatum*	55,37	47,548	belemnite	4,02	-3,19	McArthur et al. 2000[8]
P 28	Toa	*bifrons*	*fibulatum*	56,47	47,638	belemnite	3,45	-3,58	McArthur et al. 2000[8]
P 25	Toa	*bifrons*	*fibulatum*	60,00	47,926	belemnite	3,04	-3,38	McArthur et al. 2000[8]
S 436	Toa	*bifrons*	*fibulatum*	60,87	47,998	belemnite	2,40	-3,67	McArthur et al. 2000[8]
base of *crassum* sz	Toa			60,90					McArthur et al. 2000[8]
S 437A	Toa	*bifrons*	*crassum*	62,07	48,122	belemnite	2,77	-2,95	McArthur et al. 2000[8]
S 437B	Toa	*bifrons*	*crassum*	62,07	48,122	belemnite	2,64	-3,02	McArthur et al. 2000[8]
S 424	Toa	*bifrons*	*crassum*	63,27	48,247	belemnite	2,81	-3,35	McArthur et al. 2000[8]
P 24	Toa	*bifrons*	*crassum*	68,10	48,750	belemnite	4,06	-3,64	McArthur et al. 2000[8]
base of *variabilis* z	Toa			70,50					McArthur et al. 2000[8]
P 20	Toa	*variabilis*		72,55	49,130	belemnite	2,87	-3,04	McArthur et al. 2000[8]
P 17	Toa	*variabilis*		73,50	49,190	belemnite	2,63	-2,67	McArthur et al. 2000[8]
P 14	Toa	*variabilis*		76,60	49,386	belemnite	2,90	-2,62	McArthur et al. 2000[8]
P 15	Toa	*variabilis*		76,60	49,386	belemnite	3,81	-3,09	McArthur et al. 2000[8]
P 12	Toa	*variabilis*		76,80	49,399	belemnite	2,43	-2,70	McArthur et al. 2000[8]
P 11	Toa	*variabilis*		77,70	49,456	belemnite	2,80	-2,77	McArthur et al. 2000[8]
P 10	Toa	*variabilis*		78,50	49,506	belemnite	3,01	-2,15	McArthur et al. 2000[8]
P 8	Toa	*variabilis*		81,90	49,722	belemnite	1,92	-1,75	McArthur et al. 2000[8]
P 7	Toa	*variabilis*		82,30	49,747	belemnite	2,98	-3,12	McArthur et al. 2000[8]
P 5	Toa	*variabilis*		83,00	49,791	belemnite	2,91	-3,29	McArthur et al. 2000[8]
P 4	Toa	*variabilis*		84,40	49,880	belemnite	3,27	-2,93	McArthur et al. 2000[8]
P 3	Toa	*variabilis*		85,40	49,943	belemnite	2,75	-2,36	McArthur et al. 2000[8]
base of striatulum sz.	Toa			86,30					McArthur et al. 2000[8]

South Germany

our data

Aubach 37	Pli	*davoei*		32,500	brachiopod		altered	1,87	-1,95	Inn		1013	1,17	this study
Aubach 37	Pli	*davoei*		32,500	belemnite			1,88	-1,05	Inn		177	1,92	this study
Aubach 38	Pli	*davoei*		33,500	belemnite			1,77	-0,72	Inn		233	1,98	this study
Zwischenkalke	Pli	*margaritatus*	*stokesi*	35,650	brachiopod	*Rhynchonella* ?		3,20	-0,34	Inn		179	0,53	this study
Aubach 39	Pli	*margaritatus*	*stokesi*	35,980	belemnite			0,60	-1,30	Inn		213	1,94	this study
Pli Q = 3,0 m; Schwarzjura	Pli	*margaritatus*	*subnodosus*	36,500	brachiopod			3,65	-0,34	RUB		322	0,54	this study
Aubach 41	Pli	*margaritatus*	*gibbosus*	37,053	belemnite			0,70	-0,16	Inn		187	1,25	this study
Aubach 42	Pli	*margaritatus*	*gibbosus*	37,141	belemnite			1,98	-0,23	Inn		238	1,24	this study
Aubach 43	Pli	*margaritatus*	*gibbosus*	37,162	belemnite			1,32	-0,36	Inn		226	1,56	this study
Aubach 44	Pli	*margaritatus*	*gibbosus*	37,191	belemnite			3,11	-0,18	Inn		144	2,09	this study
Aubach 46	Pli	*margaritatus*	*gibbosus*	37,256	belemnite			2,27	-0,02	Inn		127	1,75	this study
Aubach 48	Pli	*margaritatus*	*gibbosus*	37,502	belemnite			1,18	0,12	Inn		153	1,95	this study
Aubach 49	Pli	*margaritatus*	*gibbosus*	37,542	belemnite			1,96	-0,20	Inn		106	1,28	this study
Aubach 50	Pli	*margaritatus*	*gibbosus*	37,614	belemnite			1,82	-0,54	Inn		166	1,74	this study
Aubach 51	Pli	*margaritatus*	*gibbosus*	37,672	belemnite			0,86	-0,02	Inn		253	1,57	this study
Costatenkalk	Pli	*spinatum*		39,000	brachiopod	*R. amalthei*		1,70	-0,73	Inn		246	0,95	this study
Aubach-Amaltheenton	Pli	*spinatum*		39,000	brachiopod	*R. variabilis*		1,93	-0,78	Inn		348	0,42	this study
31 Schw. Gmünd-Bargau	Toa	*tenuicostatum*		43,000	belemnite			2,69	-1,09	CPH	11,1	25	1,62	this study
31 Schw. Gmünd-Bargau	Toa	*tenuicostatum*		43,000	belemnite			2,64	-1,19	CPH	10,7	14	1,52	this study
31 Schw. Gmünd-Bargau	Toa	*tenuicostatum*		43,000	belemnite			2,32	-1,28	CPH	14,5	17	1,69	this study
31 Schw. Gmünd-Bargau	Toa	*tenuicostatum*		43,000	belemnite			2,28	-1,31	CPH	14,2	18	1,67	this study
33 Holzmaden	Toa	*falciferum*		44,300	brachiopod		altered	1,05	-3,93	CPH	10,5	2879	0,90	this study
33 Holzmaden	Toa	*falciferum*		44,300	brachiopod		altered	1,50	-3,57	CPH	9,6	2473	0,94	this study
35 Holzmaden	Toa	*falciferum*		45,000	pectinid			4,37	-2,43	CPH	4,5	144	1,27	this study
35 Holzmaden	Toa	*falciferum*		45,000	pectinid			4,44	-2,55	CPH	2,9	314	1,25	this study
37 Ohmden	Toa	*falciferum*		45,000	oyster			2,04	-3,58	CPH	2,1	37	0,78	this study
37 Ohmden	Toa	*falciferum*		45,000	oyster			2,05	-3,48	CPH	2,8	34	0,80	this study
37 Ohmden	Toa	*falciferum*		45,000	oyster			2,16	-3,57	CPH	2,4	28	0,85	this study
32 Göppingen-Jebenhausen	Toa	*bifrons*		47,000	belemnite			1,16	-1,67	CPH	18,5	30	1,84	this study
32 Göppingen-Jebenhausen	Toa	*bifrons*		47,000	belemnite			1,52	-1,40	CPH	16,0	58	1,84	this study
32 Göppingen-Jebenhausen	Toa	*bifrons*		47,000	belemnite			1,02	-1,56	CPH	15,2	10	1,81	this study
34 Holzmaden	Toa	*variabilis*		49,500	brachiopod			1,97	-1,55	CPH	6,5	965	0,54	this study
1 Göppingen-Jebenhausen	Toa	*thouarsense*		51,000	belemnite			0,42	-1,51	CPH	12,6	38	1,85	this study
1 Göppingen-Jebenhausen	Toa	*thouarsense*		51,000	belemnite			0,49	-1,47	CPH	12,2	15	1,86	this study
38 Holzmaden	Toa	*thourasense*		51,000	oyster		altered	2,31	-2,68	CPH	2,9	3628	0,92	this study
38 Holzmaden	Toa	*thourasense*		51,000	oyster		altered	2,50	-2,69	CPH	2,6	3524	0,86	this study
2 Heubach	Toa	*aalensis*		55,500	belemnite			-0,19	-1,51	CPH	14,3	54	1,73	this study
2 Heubach	Toa	*aalensis*		55,500	belemnite			-0,23	-1,60	CPH	15,8	132	1,72	this study
2 Heubach	Toa	*aalensis*		55,500	belemnite			-0,28	-1,49	CPH	14,5	45	1,75	this study
10 Reutlingen	Toa	*aalensis*		55,500	oyster		altered	2,66	-2,95	CPH	3,0	279	0,95	this study
10 Reutlingen	Toa	*aalensis*		55,500	oyster		altered	2,22	-2,57	CPH	3,0	322	0,88	this study
3 Aalen-Wasseralfingen	Aal	*opalinum "early"*		56,330	belemnite			0,51	-0,46	CPH	13,2	31	1,61	this study
3 Aalen-Wasseralfingen	Aal	*opalinum "early"*		56,330	belemnite			0,52	-0,35	CPH	13,1	25	1,59	this study
3 Aalen-Wasseralfingen	Aal	*opalinum "early"*		56,330	belemnite			0,47	-0,40	CPH	13,4	18	1,63	this study
4 Balingen-Zillhausen	Aal	*opalinum "late"*		56,660	belemnite			1,04	-0,54	CPH	15,3	34	1,74	this study
4 Balingen-Zillhausen	Aal	*opalinum "late"*		56,660	belemnite			0,87	-0,55	CPH	11,1	20	1,56	this study
5 Donzdorf-Grünbach	Aal	*opalinum "late"*		56,660	belemnite			1,86	-1,01	CPH	9,0	29	1,63	this study
5 Donzdorf-Grünbach	Aal	*opalinum "late"*		56,660	belemnite			2,19	-1,19	CPH	11,1	8	1,56	this study
11 Blumberg-Achdorf	Aal	*murchisonae*		60,000	pectinid			2,67	-0,64	CPH	6,5	230	1,24	this study
11 Blumberg-Achdorf	Aal	*murchisonae*		60,000	pectinid			2,36	-0,59	CPH	6,8	212	1,22	this study
12 Aalen	Aal	*murchisonae*		60,000	pectinid		altered	1,65	-3,28	CPH	6,8	1030	1,37	this study
12 Aalen	Aal	*murchisonae*		60,000	pectinid		altered	2,04	-2,46	CPH	6,6	1318	1,36	this study
13 Aalen-Wasseralfingen	Aal	*murchisonae*		60,000	pectinid		altered	1,99	-3,29	CPH	5,5	1336	1,20	this study
13 Aalen-Wasseralfingen	Aal	*murchisonae*		60,000	pectinid		altered	2,12	-3,46	CPH	5,2	1431	1,14	this study
14 Jungingen	Aal	*murchisonae*		60,000	oyster			2,94	-1,27	CPH	3,7	174	0,83	this study
14 Jungingen	Aal	*murchisonae*		60,000	oyster			2,94	-1,02	CPH	4,8	112	0,84	this study
14 Jungingen	Aal	*murchisonae*		60,000	oyster			2,96	-1,23	CPH	3,5	84	0,83	this study
23 Mössingen	Baj	*discites*		60,500	brachiopod			2,28	-0,90	CPH	4,5	241	1,23	this study
23 Mössingen	Baj	*discites*		60,500	brachiopod			2,14	-0,96	CPH	4,8	504	1,20	this study
6 Blumberg-Achdorf	Baj	*laeviuscula*		69,000	belemnite			0,99	-0,35	CPH	11,0	5	1,55	this study
6 Blumberg-Achdorf	Baj	*laeviuscula*		69,000	belemnite			1,06	-0,32	CPH	10,3	5	1,53	this study
6 Blumberg-Achdorf	Baj	*laeviuscula*		69,000	belemnite			1,05	-0,30	CPH	10,0	7	1,53	this study
15 Neidlingen	Baj	*laeviuscula*		69,000	pectinid			2,45	-0,48	CPH	6,0	902	1,12	this study
39 Laufen a. d. Eyach	Baj	*laeviuscula*		69,000	oyster			3,14	0,45	CPH	1,9	110	0,65	this study
39 Laufen a. d. Eyach	Baj	*laeviuscula*		69,000	oyster			3,13	0,21	CPH	2,5	151	0,66	this study
41 Aalen	Baj	*laeviuscula*		69,000	brachiopod			0,49	-0,93	CPH	19,0	452	1,60	this study
40 Eningen unter Achalm	Baj	*laeviuscula/sauzei*		70,000	pectinid			4,02	-0,86	CPH	6,7	326	1,29	this study
16 Eningen unter Achalm	Baj	*sauzei*		70,500	oyster			4,50	0,14	CPH	3,5	66	0,80	this study
16 Eningen unter Achalm	Baj	*sauzei*		70,500	oyster			4,52	0,05	CPH	3,2	78	0,80	this study
16 Eningen unter Achalm	Baj	*sauzei*		70,500	oyster			4,42	0,09	CPH	3,2	70	0,78	this study
17 Eningen unter Achalm	Baj	*sauzei*		70,500	pectinid			3,68	-0,16	CPH	4,6	59	1,35	this study
17 Eningen unter Achalm	Baj	*sauzei*		70,500	pectinid			3,00	-0,32	CPH	4,3	263	1,30	this study
17 Eningen unter Achalm	Baj	*sauzei*		70,500	pectinid			3,23	-0,08	CPH	6,7	196	1,31	this study
8 Talheim am Lupfen	Baj	*humphriesianum*		72,500	belemnite			0,20	0,12	CPH	9,7	3	1,38	this study
8 Talheim am Lupfen	Baj	*humphriesianum*		72,500	belemnite			-0,20	-0,02	CPH	10,5	5	1,35	this study

9 Neuffen	Baj	*humphriesianum*		72,500	belemnite		3,10	0,22	CPH	9,9	58	1,57	this study
9 Neuffen	Baj	*humphriesianum*		72,500	belemnite		3,03	0,17	CPH	9,1	77	1,47	this study
18 Talheim am Lupfen	Baj	*humphriesianum*		72,500	limide	altered	-0,13	-1,50	CPH	21,9	677	0,92	this study
19 Talheim am Lupfen	Baj	*humphriesianum*		72,500	oyster	altered	1,11	-2,08	CPH	11,1	885	0,58	this study
19 Talheim am Lupfen	Baj	*humphriesianum*		72,500	oyster		3,27	-0,32	CPH	6,8	111	0,84	this study
20 Talheim am Lupfen	Baj	*humphriesianum*		72,500	pinnid		3,46	0,42	CPH	21,8	128	0,91	this study
20 Talheim am Lupfen	Baj	*humphriesianum*		72,500	pinnid		3,85	0,39	CPH	15,6	107	0,90	this study
24 Gosheim	Baj	*humphriesianum*		72,500	brachiopod	altered	1,28	-1,56	CPH	15,5	1358	0,81	this study
25 Gosheim	Baj	*humphriesianum*		72,500	brachiopod		2,75	-0,64	CPH	3,0	43	0,97	this study
25 Gosheim	Baj	*humphriesianum*		72,500	brachiopod		2,67	-0,69	CPH	5,3	60	0,99	this study

literature data

Roadcut, D

GMB14	Pli	*davoei*	*maculatum*	32,500	belemnite	1,77	-0,53	Jenkyns et al. 2002[5]
GMB15	Pli	*davoei*	*maculatum*	32,850	belemnite	0,99	-0,89	Jenkyns et al. 2002[5]
GMB16	Pli	*davoei*	*figulinum*	34,530	belemnite	1,05	-0,57	Jenkyns et al. 2002[5]
GMB17	Pli	*margaritatus*	*stokesi*	35,090	belemnite	1,00	-0,52	Jenkyns et al. 2002[5]
GMB18	Pli	*margaritatus*	*stokesi*	35,280	belemnite	2,01	-0,86	Jenkyns et al. 2002[5]
GMB19	Pli	*margaritatus*	*stokesi*	35,400	belemnite	2,94	-0,15	Jenkyns et al. 2002[5]
GMB20	Pli	*margaritatus*	*stokesi*	35,690	belemnite	2,01	-0,09	Jenkyns et al. 2002[5]
GMB21	Pli	*margaritatus*	*stokesi*	35,790	belemnite	2,48	-0,28	Jenkyns et al. 2002[5]
GMB22	Pli	*margaritatus*	*subnodosus*	36,370	belemnite	2,12	0,30	Jenkyns et al. 2002[5]
GMB23	Pli	*margaritatus*	*subnodosus*	36,490	belemnite	1,62	-0,01	Jenkyns et al. 2002[5]
GMB24	Pli	*margaritatus*	*gibbosus*	37,200	belemnite	1,49	-0,05	Jenkyns et al. 2002[5]
GMB25	Pli	*margaritatus*	*gibbosus*	37,300	belemnite	1,61	0,07	Jenkyns et al. 2002[5]
GMB26	Pli	*margaritatus*	*gibbosus*	37,520	belemnite	0,44	-0,18	Jenkyns et al. 2002[5]
GMB28	Pli	*spinatum*	*apyrenum*	38,570	belemnite	1,90	0,11	Jenkyns et al. 2002[5]
GMB29	Pli	*spinatum*	*apyrenum*	38,710	belemnite	1,32	-0,41	Jenkyns et al. 2002[5]
GMB30	Pli	*spinatum*	*apyrenum*	38,950	belemnite	1,12	-0,51	Jenkyns et al. 2002[5]

Portugal and Spain

literature data

Carbo Mondego, Portugal

CM1	Aal		*limitatum/formosum*	64,400	belemnite	0,66	-0,39	Jenkyns et al. 2002[5]
CM3	Aal		*limitatum/formosum*	64,650	belemnite	0,64	-0,64	Jenkyns et al. 2002[5]
CM2	Aal		*limitatum/formosum*	64,900	belemnite	0,00	-0,52	Jenkyns et al. 2002[5]
CM4	Baj	*discites*		65,275	belemnite	-0,01	-0,52	Jenkyns et al. 2002[5]
CM7	Baj	*discites*		65,750	belemnite	0,12	-0,46	Jenkyns et al. 2002[5]
CM5	Baj	*ovalis*		66,070	belemnite	0,87	-0,49	Jenkyns et al. 2002[5]
CM6	Baj	*ovalis*		66,250	belemnite	0,49	-0,36	Jenkyns et al. 2002[5]
CM8a	Baj	*ovalis*		66,390	belemnite	0,74	-0,79	Jenkyns et al. 2002[5]
CM8b	Baj	*ovalis*		66,390	belemnite	0,92	-0,02	Jenkyns et al. 2002[5]
CM9a	Baj	*ovalis*		66,440	belemnite	1,17	-0,85	Jenkyns et al. 2002[5]
CM9b	Baj	*ovalis*		66,440	belemnite	0,82	-0,69	Jenkyns et al. 2002[5]
CM28	Baj	*ovalis*		66,480	belemnite	0,80	-0,49	Jenkyns et al. 2002[5]
CM27	Baj	*ovalis*		66,855	belemnite	0,76	-0,53	Jenkyns et al. 2002[5]
CM26	Baj		*sayni*	67,200	belemnite	1,78	-0,37	Jenkyns et al. 2002[5]
CM25	Baj		*sayni*	67,600	belemnite	0,68	-0,44	Jenkyns et al. 2002[5]
CM24	Baj		*trigonalis*	68,150	belemnite	1,23	-0,52	Jenkyns et al. 2002[5]
CM11a	Baj		*trigonalis*	68,150	belemnite	1,55	-0,58	Jenkyns et al. 2002[5]
CM11b	Baj		*trigonalis*	68,150	belemnite	1,64	-0,39	Jenkyns et al. 2002[5]
CM12	Baj		*trigonalis*	68,920	belemnite	1,37	-0,12	Jenkyns et al. 2002[5]
CM10a	Baj		*laeviuscula*	69,080	belemnite	0,57	-0,28	Jenkyns et al. 2002[5]
CM10b	Baj		*laeviuscula*	69,080	belemnite	0,61	-0,43	Jenkyns et al. 2002[5]
CM13	Baj		*laeviuscula*	69,250	belemnite	1,66	-0,94	Jenkyns et al. 2002[5]
CM14	Baj		*laeviuscula*	69,400	belemnite	1,01	-0,52	Jenkyns et al. 2002[5]
CM15	Baj		*laeviuscula*	69,650	belemnite	1,09	-0,23	Jenkyns et al. 2002[5]
CM16	Baj		*laeviuscula*	69,920	belemnite	1,66	-0,43	Jenkyns et al. 2002[5]
CM17	Baj	*sauzei*		70,130	belemnite	1,35	-0,25	Jenkyns et al. 2002[5]
CM18	Baj	*sauzei*		70,250	belemnite	1,05	-0,14	Jenkyns et al. 2002[5]
CM19	Baj	*sauzei*		70,375	belemnite	0,72	-0,19	Jenkyns et al. 2002[5]
CM20	Baj	*sauzei*		70,605	belemnite	0,44	-0,25	Jenkyns et al. 2002[5]
CM21a	Baj	*sauzei*		70,840	belemnite	0,54	-0,14	Jenkyns et al. 2002[5]
CM21b	Baj	*sauzei*		70,840	belemnite	0,56	-0,33	Jenkyns et al. 2002[5]
CM22c	Baj		*romani*	71,000	belemnite	0,67	-0,49	Jenkyns et al. 2002[5]

Peniche, Portugal

BB11-1	Pli	*Spinatum*	-	4,47	39,500	brachiopod	Rhynchonellid	2,54	-0,50	Suan et al. 2008[9]
BB16	Pli	*Spinatum*	-	5,61	39,630	brachiopod	Rhynchonellid	2,18	-0,89	Suan et al. 2008[9]
BB13	Pli	*Spinatum*	-	6,09	39,690	brachiopod	Terebratulid	1,63	-1,01	Suan et al. 2008[9]
B1	Pli	*Spinatum*	-	6,90	39,790	brachiopod	*Lobothyris arcta*	1,38	-0,97	Suan et al. 2008[9]
BB17-2	Pli	*Spinatum*	-	8,45	40,000	brachiopod	*Lobothyris arcta*	1,35	-1,06	Suan et al. 2008[9]
BB17-3	Pli	*Spinatum*	-	8,45	40,000	brachiopod	*Lobothyris arcta*	1,76	-1,04	Suan et al. 2008[9]
BB43bis	Toa	*Tenuicostatum*	mirabile	8,63	40,000	brachiopod	Rhynchonellid	-0,11	-2,22	Suan et al. 2008[9]
Pen06-B1	Toa	*Tenuicostatum*	mirabile	8,63	40,000	brachiopod	Rhynchonellid	2,87	-0,96	Suan et al. 2008[9]
Pen06-B2	Toa	*Tenuicostatum*	mirabile	8,63	40,000	brachiopod	Rhynchonellid	2,02	-1,48	Suan et al. 2008[9]
BB45	Toa	*Tenuicostatum*	semicelatum	9,20	40,133	brachiopod	Spiriniferid	1,07	-1,90	Suan et al. 2008[9]
B3bis	Toa	*Tenuicostatum*	semicelatum	9,68	40,400	brachiopod	*Nannirhynchia pygmoea*	2,99	-1,67	Suan et al. 2008[9]
BB46	Toa	*Tenuicostatum*	semicelatum	10,35	40,800	brachiopod	Terebratulid	2,24	-1,50	Suan et al. 2008[9]
BB50	Toa	*Tenuicostatum*	semicelatum	12,18	41,333	brachiopod	*Nannirhynchia pygmoea*	3,51	-1,30	Suan et al. 2008[9]
BB53	Toa	*Tenuicostatum*	semicelatum	13,34	41,733	brachiopod	*Nannirhynchia pygmoea*	3,82	-1,36	Suan et al. 2008[9]
B6	Toa	*Tenuicostatum*	semicelatum	15,52	42,533	brachiopod	*Nannirhynchia pygmoea*	3,72	-1,69	Suan et al. 2008[9]
BB56	Toa	*Falciferum*	*exaratum?*	22,40	44,094	brachiopod	Rhynchonellid	0,53	-2,53	Suan et al. 2008[9]
BB29	Toa	*Falciferum*	*exaratum?*	30,37	44,341	brachiopod	*S. bouchardi*	2,54	-2,76	Suan et al. 2008[9]
BB32	Toa	*Falciferum*	*exaratum?*	31,16	44,376	brachiopod	*S. bouchardi*	1,93	-2,52	Suan et al. 2008[9]
BB34	Toa	*Falciferum*	*exaratum?*	32,42	44,400	brachiopod	*S. bouchardi*	3,13	-2,36	Suan et al. 2008[9]
BB36	Toa	*Falciferum*	*falciferum?*	33,96	44,471	brachiopod	*S. bouchardi*	3,51	-2,09	Suan et al. 2008[9]
BB37	Toa	*Falciferum*	*falciferum?*	34,77	44,565	brachiopod	*S. bouchardi*	4,05	-2,20	Suan et al. 2008[9]
BB20	Toa	*Falciferum*	*falciferum?*	38,62	44,953	brachiopod	*S. bouchardi*	5,32	-1,97	Suan et al. 2008[9]
B7bis	Toa	*Falciferum*	*falciferum?*	39,62	45,059	brachiopod	*S. bouchardi*	5,45	-2,34	Suan et al. 2008[9]
B7-1	Toa	*Falciferum*	*falciferum?*	39,73	45,071	brachiopod	*S. bouchardi*	5,54	-2,23	Suan et al. 2008[9]
B7-2	Toa	*Falciferum*	*falciferum?*	39,73	45,071	brachiopod	*S. bouchardi*	5,72	-1,98	Suan et al. 2008[9]
BB25	Toa	*Falciferum*	*falciferum?*	45,62	45,647	brachiopod	*Telothyris jauberti*	4,98	-2,20	Suan et al. 2008[9]

Fuentelsaz, Spain

FZ-58	Pli		*aalensis*	55,40	55,400	brachiopod		1,30	-1,85	Cresta et al. 2001[10]
FZ-66	Pli		*aalensis*	55,51	55,510	brachiopod		1,56	-1,74	Cresta et al. 2001[10]
FZ-72	Pli		*aalensis*	55,59	55,590	brachiopod		1,40	-1,48	Cresta et al. 2001[10]
FZ-86	Pli		*aalensis*	55,71	55,710	brachiopod		0,76	-3,04	Cresta et al. 2001[10]
FZ-87	Pli		*aalensis*	55,73	55,730	brachiopod		0,54	-2,34	Cresta et al. 2001[10]
FZ-88	Pli		*aalensis*	55,75	55,750	brachiopod		0,91	-3,07	Cresta et al. 2001[10]
FZ-98b	Pli		*aalensis*	55,82	55,820	brachiopod		0,43	-3,86	Cresta et al. 2001[10]
FZ-104b	Pli		*aalensis*	55,91	55,910	brachiopod		0,65	-2,85	Cresta et al. 2001[10]
FZ-104b	Pli		*aalensis*	55,91	55,910	brachiopod		0,70	-2,53	Cresta et al. 2001[10]
FZ-110	Toa	*opalinum*	-	56,10	56,100	brachiopod		1,55	-2,20	Cresta et al. 2001[10]
FZ-122	Toa	*opalinum*	-	56,22	56,220	brachiopod		-1,43	-4,09	Cresta et al. 2001[10]
FZ-128	Toa	*opalinum*	-	56,31	56,310	brachiopod		0,96	-1,99	Cresta et al. 2001[10]
FZ-143	Toa	*opalinum*	-	56,53	56,530	brachiopod		1,30	-3,13	Cresta et al. 2001[10]

Camino, Santiurde de Reinosa, San Andrés; Spain

CAM-P-3-2	Pli	*davoei*	*maculatum*	58,40		belemnite			-3,23	Rosales et al. 2004[11]
CAM-P-3-4	Pli	*davoei*		58,40		belemnite			-1,99	Rosales et al. 2004[11]
SANA-8-1	Pli	*davoei*	*maculatum*	62,40		belemnite			-1,80	Rosales et al. 2004[11]
SANA-8-2	Pli	*davoei*	*maculatum*	62,40		belemnite			-1,13	Rosales et al. 2004[11]
CAM-P-5	Pli	*davoei*	*capricornus*	63,40		belemnite			-1,97	Rosales et al. 2004[11]
SANA-14	Pli	*davoei*	*capricornus*	63,60		belemnite			-1,49	Rosales et al. 2004[11]
SANA-18	Pli	*davoei*	*capricornus*	65,00		belemnite			-0,48	Rosales et al. 2004[11]
SANA-18 B	Pli	*davoei*	*capricornus*	65,00		belemnite			-1,59	Rosales et al. 2004[11]
SANA-20-B	Pli	*davoei*	*figulinum*	66,50		belemnite			-1,32	Rosales et al. 2004[11]
SANA-26	Pli	*stokesi*	*monestieri*	70,00		belemnite			-1,32	Rosales et al. 2004[11]
SANA-34	Pli	*stokesi*	*monestieri*	72,50		belemnite			-1,24	Rosales et al. 2004[11]
SANA-34-1	Pli	*stokesi*	*monestieri*	72,60		belemnite		1,18	-1,42	Rosales et al. 2001[12], 2004[11]
SANA-34-2b	Pli	*stokesi*	*monestieri*	72,90		belemnite			-1,28	Rosales et al. 2004[11]
SANA-34-2	Pli	*stokesi*	*monestieri*	72,90		belemnite		1,78	-1,08	Rosales et al. 2001[12], 2004[11]
CAM-P-6-1	Pli	*stokesi*	*monestieri*	73,50		belemnite			-1,90	Rosales et al. 2004[11]
SANA-40	Pli	*stokesi*	*monestieri*	74,60		belemnite			-1,93	Rosales et al. 2004[11]
SANA-43 -1	Pli	*stokesi*	*monestieri*	75,60		belemnite		-0,09	-1,25	Rosales et al. 2001[12], 2004[11]
SANA-43 -2	Pli	*stokesi*	*monestieri*	75,60		belemnite		1,03	-1,62	Rosales et al. 2001[12], 2004[11]
SANA-44A	Pli	*stokesi*	*monestieri*	77,00		belemnite		0,79	-0,96	Rosales et al. 2001[12], 2004[11]
SANA-44B	Pli	*stokesi*	*monestieri*	77,00		belemnite		1,69	-1,31	Rosales et al. 2001[12], 2004[11]
CAM-P-8	Pli	*stokesi*	*monestieri*	77,80		belemnite			-1,62	Rosales et al. 2004[11]
CAM-P-9	Pli	*stokesi*	*monestieri*	79,30		belemnite			-0,25	Rosales et al. 2004[11]
CAM-P-9 -2	Pli	*stokesi*	*monestieri*	79,30		belemnite			-0,21	Rosales et al. 2004[11]
SANA-50-1	Pli	*stokesi*	*celebratum*	81,00		belemnite		-0,31	-1,44	Rosales et al. 2001[12], 2004[11]
SANA-50-2	Pli	*stokesi*	*celebratum*	81,10		belemnite		1,10	-1,59	Rosales et al. 2001[12], 2004[11]
CAM-P-7-3	Pli	*stokesi*	*celebratum*	84,20		belemnite		0,73	-1,52	Rosales et al. 2001[12], 2004[11]
CAM-P-7-4	Pli	*stokesi*	*celebratum*	84,20		belemnite		0,79	-1,65	Rosales et al. 2001[12], 2004[11]
SANA-52-2	Pli	*margaritatus*	*subnodosus*	86,50		belemnite		1,60	-0,27	Rosales et al. 2001[12], 2004[11]
SANA-52-1	Pli	*margaritatus*	*subnodosus*	86,60		belemnite		1,54	-0,83	Rosales et al. 2001[12], 2004[11]
SANA-52-A	Pli	*margaritatus*	*subnodosus*	87,20		belemnite			-0,83	Rosales et al. 2004[11]

SANA-52 C -1	Pli	*margaritatus*	*subnodosus*	88,00	belemnite	1,01	-0,69	Rosales et al. 2001[12], 2004[11]
SANA-52 C -3	Pli	*margaritatus*	*subnodosus*	88,10	belemnite	-0,66	-0,74	Rosales et al. 2001[12], 2004[11]
SANA-52-C -2	Pli	*margaritatus*	*subnodosus*	88,10	belemnite	1,12	-1,15	Rosales et al. 2001[12], 2004[11]
SANA-52-D -2	Pli	*margaritatus*	*subnodosus*	88,60	belemnite	1,34	-0,46	Rosales et al. 2001[12], 2004[11]
SANA-52 D -1	Pli	*margaritatus*	*subnodosus*	88,70	belemnite	2,03	0,07	Rosales et al. 2001[12], 2004[11]
CAM-P-10-2	Pli	*margaritatus*	*subnodosus*	89,50	belemnite	0,79	-1,74	Rosales et al. 2001[12], 2004[11]
SANA-54	Pli	*margaritatus*	*subnodosus*	90,20	belemnite		-0,31	Rosales et al. 2004[11]
CAM-P-11-1	Pli	*margaritatus*	*subnodosus*	92,30	belemnite		-0,25	Rosales et al. 2004[11]
CAM-P-11-2	Pli	*margaritatus*	*subnodosus*	92,30	belemnite		-0,92	Rosales et al. 2004[11]
SANA-58-A	Pli	*margaritatus*	*subnodosus*	92,60	belemnite	1,75	-0,32	Rosales et al. 2001[12], 2004[11]
SANA-58-B	Pli	*margaritatus*	*subnodosus*	92,60	belemnite	1,05	-0,13	Rosales et al. 2001[12], 2004[11]
SANA-59-1	Pli	*margaritatus*	*subnodosus*	93,20	belemnite	-0,20	-0,27	Rosales et al. 2001[12], 2004[11]
SANA-59-2	Pli	*margaritatus*	*subnodosus*	93,20	belemnite	1,94	-0,03	Rosales et al. 2001[12], 2004[11]
SANA-59-4	Pli	*margaritatus*	*subnodosus*	93,20	belemnite	1,46	-0,72	Rosales et al. 2001[12], 2004[11]
SANA-59-5	Pli	*margaritatus*	*subnodosus*	93,20	belemnite	1,80	0,10	Rosales et al. 2001[12], 2004[11]
SANA-59-6	Pli	*margaritatus*	*subnodosus*	93,20	belemnite	1,16	-0,35	Rosales et al. 2001[12], 2004[11]
SANA-59-3	Pli	*margaritatus*	*subnodosus*	93,40	belemnite		-0,22	Rosales et al. 2004[11]
SANA-60-2	Pli	*margaritatus*	*subnodosus*	94,40	belemnite	1,10	-0,31	Rosales et al. 2001[12], 2004[11]
SANA-60-7	Pli	*margaritatus*	*subnodosus*	94,50	belemnite	1,27	-0,13	Rosales et al. 2001[12], 2004[11]
SANA-60-3	Pli	*margaritatus*	*subnodosus*	94,50	belemnite		-0,68	Rosales et al. 2004[11]
SANA-60-5	Pli	*margaritatus*	*subnodosus*	94,60	belemnite	1,27	-0,04	Rosales et al. 2001[12], 2004[11]
SANA-60-6	Pli	*margaritatus*	*subnodosus*	94,60	belemnite		-0,18	Rosales et al. 2004[11]
SANA-60-1	Pli	*margaritatus*	*subnodosus*	94,70	belemnite	1,20	-0,13	Rosales et al. 2001[12], 2004[11]
SANA-60-4	Pli	*margaritatus*	*subnodosus*	94,70	belemnite	0,91	-0,42	Rosales et al. 2001[12], 2004[11]
SANA-62-1	Pli	*margaritatus*	*subnodosus*	97,20	belemnite	0,43	-1,03	Rosales et al. 2001[12], 2004[11]
SANA-62-2	Pli	*margaritatus*	*subnodosus*	97,20	belemnite	0,47	-0,29	Rosales et al. 2001[12], 2004[11]
CAM-P-12-1	Pli	*margaritatus*	*subnodosus*	97,80	belemnite		-0,02	Rosales et al. 2004[11]
CAM-P-12-2	Pli	*margaritatus*	*subnodosus*	97,80	belemnite		-0,04	Rosales et al. 2004[11]
CAM-P-13	Pli	*margaritatus*	*subnodosus*	98,90	belemnite		-0,24	Rosales et al. 2004[11]
CAM-P-14-1	Pli	*margaritatus*	*subnodosus*	105,30	belemnite		-1,66	Rosales et al. 2004[11]
CAM-P-14-5	Pli	*margaritatus*	*subnodosus*	105,30	belemnite		-1,97	Rosales et al. 2004[11]
CAM-P-14-4	Pli	*margaritatus*	*subnodosus*	105,30	belemnite		-1,88	Rosales et al. 2004[11]
CAM-P-17-1	Pli	*margaritatus*	*subnodosus*	115,60	belemnite		-1,10	Rosales et al. 2004[11]
CAM-P-17-2	Pli	*margaritatus*	*subnodosus*	115,60	belemnite		-1,03	Rosales et al. 2004[11]
CAM-P-18-B	Pli	*margaritatus*	*gibbosus*	121,40	belemnite	0,43	-0,86	Rosales et al. 2001[12], 2004[11]
CAM-P-18A	Pli	*margaritatus*	*gibbosus*	121,40	belemnite	0,67	-0,71	Rosales et al. 2001[12], 2004[11]
CAM-P-19A-1	Pli	*spinatum*	*solare*	125,10	belemnite	1,03	-0,26	Rosales et al. 2001[12], 2004[11]
CAM-P-19A-4	Pli	*spinatum*	*solare*	125,10	belemnite	0,27	-0,18	Rosales et al. 2001[12], 2004[11]
CAM-P-19B-1	Pli	*spinatum*	*solare*	125,10	belemnite		-0,18	Rosales et al. 2004[11]
CAM-P-19B-2	Pli	*spinatum*	*solare*	125,10	belemnite		-1,14	Rosales et al. 2004[11]
CAM-P-20	Pli	*spinatum*	*solare*	126,90	belemnite		-0,25	Rosales et al. 2004[11]
CAM-P-20-1	Pli	*spinatum*	*solare*	126,90	belemnite		-0,35	Rosales et al. 2004[11]
CAM-P-170	Pli	*spinatum*	*solare*	127,50	belemnite		-0,02	Rosales et al. 2004[11]
CAM-P-171	Pli	*spinatum*	*solare*	127,70	belemnite		-0,49	Rosales et al. 2004[11]
CAM-P-172	Pli	*spinatum*	*solare*	127,90	belemnite		0,11	Rosales et al. 2004[11]
CAM-P-173 (C)	Pli	*spinatum*	*solare*	128,10	belemnite		-0,36	Rosales et al. 2004[11]
CAM-P-173 (D) -2	Pli	*spinatum*	*solare*	128,10	belemnite	1,69	0,42	Rosales et al. 2001[12], 2004[11]
CAM-P-173 (D) -3	Pli	*spinatum*	*solare*	128,10	belemnite		0,34	Rosales et al. 2004[11]
CAM-P-173 (D) -4	Pli	*spinatum*	*solare*	128,10	belemnite	1,62	0,33	Rosales et al. 2001[12], 2004[11]
CAM-P-173 (D) -1	Pli	*spinatum*	*solare*	128,10	belemnite	1,31	0,22	Rosales et al. 2001[12], 2004[11]
CAM-P-173	Pli	*spinatum*	*solare*	128,10	belemnite		-0,50	Rosales et al. 2004[11]
CAM-P-175 (B)	Pli	*spinatum*	*solare*	129,10	belemnite	0,90	-0,25	Rosales et al. 2001[12], 2004[11]
CAM-P-175 (C)	Pli	*spinatum*	*solare*	129,10	belemnite	0,96	0,09	Rosales et al. 2001[12], 2004[11]
CAM-P-175	Pli	*spinatum*	*solare*	129,10	belemnite		-0,49	Rosales et al. 2004[11]
CAM-P-176 (A)	Pli	*spinatum*	*solare*	129,60	belemnite		0,74	Rosales et al. 2004[11]
CAM-P-176 (B)	Pli	*spinatum*	*solare*	129,60	belemnite		-0,13	Rosales et al. 2004[11]
CAM-P-21	Pli	*spinatum*	*hawskerense*	132,20	belemnite		-0,39	Rosales et al. 2004[11]
CAM-P-22	Pli	*spinatum*	*hawskerense*	134,20	belemnite		-0,04	Rosales et al. 2004[11]
CAM-P-23	Pli	*spinatum*	*hawskerense*	137,10	belemnite		0,12	Rosales et al. 2004[11]
CAM-T-1	Toa	*tenuicostatum*	*mirabile*	141,70	belemnite		-0,90	Rosales et al. 2004[11]
CAM-T-2	Toa	*tenuicostatum*	*mirabile*	142,80	belemnite		-1,07	Rosales et al. 2004[11]
CAM-T-3-1	Toa	*tenuicostatum*	*semicelatum*	144,60	belemnite	1,47	-1,55	Rosales et al. 2001[12], 2004[11]
CAM-T-3-3	Toa	*tenuicostatum*	*semicelatum*	144,60	belemnite	1,55	-0,66	Rosales et al. 2001[12], 2004[11]
CAM-T-3-5	Toa	*tenuicostatum*	*semicelatum*	144,60	belemnite	1,74	-0,65	Rosales et al. 2001[12], 2004[11]
CAM-T-3-7	Toa	*tenuicostatum*	*semicelatum*	144,60	belemnite	0,61	-0,79	Rosales et al. 2001[12], 2004[11]
CAM-T-4-4	Toa	*tenuicostatum*	*semicelatum*	145,50	belemnite	1,62	-0,77	Rosales et al. 2001[12], 2004[11]
CAM-T-4-A	Toa	*tenuicostatum*	*semicelatum*	145,50	belemnite	1,81	-0,67	Rosales et al. 2001[12], 2004[11]
CAM-T-4	Toa	*tenuicostatum*	*semicelatum*	145,50	belemnite		-0,90	Rosales et al. 2004[11]
CAM-T-4-3	Toa	*tenuicostatum*	*semicelatum*	145,50	belemnite		-1,64	Rosales et al. 2004[11]
CAM-T-4-B	Toa	*tenuicostatum*	*semicelatum*	145,50	belemnite		-1,24	Rosales et al. 2004[11]
CAM-T-6-1	Toa	*tenuicostatum*	*semicelatum*	145,80	belemnite		-1,09	Rosales et al. 2004[11]
CAM-T-234	Toa	*tenuicostatum*	*semicelatum*	146,50	belemnite		-1,20	Rosales et al. 2004[11]
CT-236(1)	Toa	*tenuicostatum*	*semicelatum*	146,80	belemnite		-1,33	Rosales et al. 2004[11]
CT-236(2)	Toa	*tenuicostatum*	*semicelatum*	146,80	belemnite		-0,91	Rosales et al. 2004[11]
CAM-T-11-1	Toa	*serpentinus*	*strangewaysi*	151,10	belemnite	1,83	-2,52	Rosales et al. 2001[12], 2004[11]
CAM-T-11-2	Toa	*serpentinus*	*strangewaysi*	151,10	belemnite	1,66	-2,83	Rosales et al. 2001[12], 2004[11]
CAM-T-247	Toa	*serpentinus*	*strangewaysi*	151,20	belemnite		-2,57	Rosales et al. 2004[11]
CAM-T-7-1	Toa	*serpentinus*	*strangewaysi*	151,30	belemnite		-2,95	Rosales et al. 2004[11]
CAM-T-7-2	Toa	*serpentinus*	*strangewaysi*	151,30	belemnite		-3,20	Rosales et al. 2004[11]

CAM-T-8	Toa	*serpentinus*	*strangewaysi*	151,70	belemnite		-1,86	Rosales et al. 2004[11]
CAM-T-9-2	Toa	*serpentinus*	*strangewaysi*	152,40	belemnite		-2,24	Rosales et al. 2004[11]
CAM-T-9-1	Toa	*serpentinus*	*strangewaysi*	152,40	belemnite		-2,60	Rosales et al. 2004[11]
CAM-T-261	Toa	*serpentinus*	*falciferum*	153,50	belemnite		-1,89	Rosales et al. 2004[11]
CAM-T-10-1	Toa	*serpentinus*	*falciferum*	153,90	belemnite	2,12	-1,88	Rosales et al. 2001[12], 2004[11]
CAM-T-10-2	Toa	*serpentinus*	*falciferum*	153,90	belemnite	2,47	-1,81	Rosales et al. 2001[12], 2004[11]
CAM-T-10-4	Toa	*serpentinus*	*falciferum*	153,90	belemnite	2,61	-1,93	Rosales et al. 2001[12], 2004[11]
CAM-T-266/252	Toa	*serpentinus*	*falciferum*	155,30	belemnite		-2,57	Rosales et al. 2004[11]
CAM-T-258/274	Toa	*serpentinus*	*falciferum*	157,50	belemnite		-1,36	Rosales et al. 2004[11]
CAM-T-275/259	Toa	*serpentinus*	*falciferum*	157,90	belemnite	1,14	-2,20	Rosales et al. 2001[12], 2004[11]
CAM-T-275/259 (A)	Toa	*serpentinus*	*falciferum*	157,90	belemnite	1,55	-2,13	Rosales et al. 2001[12], 2004[11]
